# Creating resistance to avian influenza infection through genome editing of the ANP32 gene family

**DOI:** 10.1038/s41467-023-41476-3

**Published:** 2023-10-10

**Authors:** Alewo Idoko-Akoh, Daniel H. Goldhill, Carol M. Sheppard, Dagmara Bialy, Jessica L. Quantrill, Ksenia Sukhova, Jonathan C. Brown, Samuel Richardson, Ciara Campbell, Lorna Taylor, Adrian Sherman, Salik Nazki, Jason S. Long, Michael A. Skinner, Holly Shelton, Helen M. Sang, Wendy S. Barclay, Mike J. McGrew

**Affiliations:** 1 The Roslin Institute and Royal (Dick) School of Veterinary Studies, University of Edinburgh, Easter Bush Campus, Edinburgh, UK; 2 Department of Infectious Disease, Imperial College London, London, UK; 3 Royal Veterinary College, London, UK; 4 The Pirbright Institute, Pirbright, UK; 5 Division of Virology, National Institute for Biological Standards and Control, Potters Bar, UK

**Keywords:** Genetic engineering, Genetic engineering, Infection

## Abstract

Chickens genetically resistant to avian influenza could prevent future outbreaks. In chickens, influenza A virus (IAV) relies on host protein ANP32A. Here we use CRISPR/Cas9 to generate homozygous gene edited (GE) chickens containing two ANP32A amino acid substitutions that prevent viral polymerase interaction. After IAV challenge, 9/10 edited chickens remain uninfected. Challenge with a higher dose, however, led to breakthrough infections. Breakthrough IAV virus contained IAV polymerase gene mutations that conferred adaptation to the edited chicken ANP32A. Unexpectedly, this virus also replicated in chicken embryos edited to remove the entire ANP32A gene and instead co-opted alternative ANP32 protein family members, chicken ANP32B and ANP32E. Additional genome editing for removal of ANP32B and ANP32E eliminated all viral growth in chicken cells. Our data illustrate a first proof of concept step to generate IAV-resistant chickens and show that multiple genetic modifications will be required to curtail viral escape.

## Abstract (web summary)

In chickens, influenza A virus relies on host protein ANP32A. Here the authors use CRISPR/Cas9 to generate homozygous gene edited chickens containing two ANP32A amino acid substitutions that prevent viral polymerase interaction.

## Introduction

Influenza A viruses (IAVs) are enveloped negative-sense single-stranded RNA viruses which infect birds and mammals causing respiratory disease and significant economic losses^1–3^. Avian influenza in poultry poses a constant zoonotic threat to humans with the possibility for evolution of novel IAVs with pandemic potential^4^. At the current time, a highly pathogenic avian influenza virus H5N1 subtype clade 2.3.4.4b is geographically dispersed across Asia, Europe, Africa and the Americas, associated with wild birds die offs, devastating impacts on farmed poultry and numerous incursions into mammals including some human cases and deaths^5^. Poultry vaccination for control of avian influenza is not reliable due to the rapid antigenic drift of field viruses and is controversial due to political and economic implications^6^.

The heterotrimeric viral RNA-dependent RNA polymerase, comprised of PB1, PB2 and PA proteins, is responsible for transcription and replication of the IAV genome in the host cell nucleus, but depends on essential support from the host-encoded ANP32 family of proteins, ANP32A and ANP32B^7^. An important difference exists between avian and mammalian ANP32A, whereby the avian protein has an additional 33-amino-acid sequence between its N-terminal leucine rich region (LRR) and the C-terminal low complexity acidic region (LCAR) domains. The shorter mammalian ANP32 proteins do not efficiently support avian influenza polymerase and this accounts for a host range restriction that limits the infection of humans when exposed to infected birds^8^. However, the virus can acquire mutations, usually in PB2 or PA subunits, that adapt polymerase to use mammalian ANP32 proteins, or can acquire these segments from a mammalian-adapted virus by a process of reassortment, and this is a prelude for the emergence of pandemic influenza^1^. In human cells, ANP32A and ANP32B serve redundant roles to support influenza polymerase^9^. In chicken cells, ANP32A is solely responsible for the pro-viral function while ANP32B is inactive^10^. In both species, ANP32E is suggested to have an antiviral effect^11^. Replication of the viral genome requires the formation of a replicative platform consisting of two heterotrimeric polymerase molecules bridged by ANP32A to form an asymmetric dimer^12^. The amino acids 129N and 130D in the fifth leucine-rich repeat (LRR) of ANP32A are critical for this interaction. Chicken ANP32B contains the amino acids 129I and 130N, and does not interact with the viral polymerase, accounting for its inability to support polymerase activity^10,13^. Here, we tested the hypothesis that IAV infection and transmission will be abrogated in chickens containing the two N129I and D130N substitutions engineered into ANP32A. We used genome editing (GE) to alter these residues in the ANP32A gene and generated ANP32A-GE chickens to test for resistance to avian influenza infection.

## Results

### Generation of genome-edited primordial germ cells

**Fig. 1:**
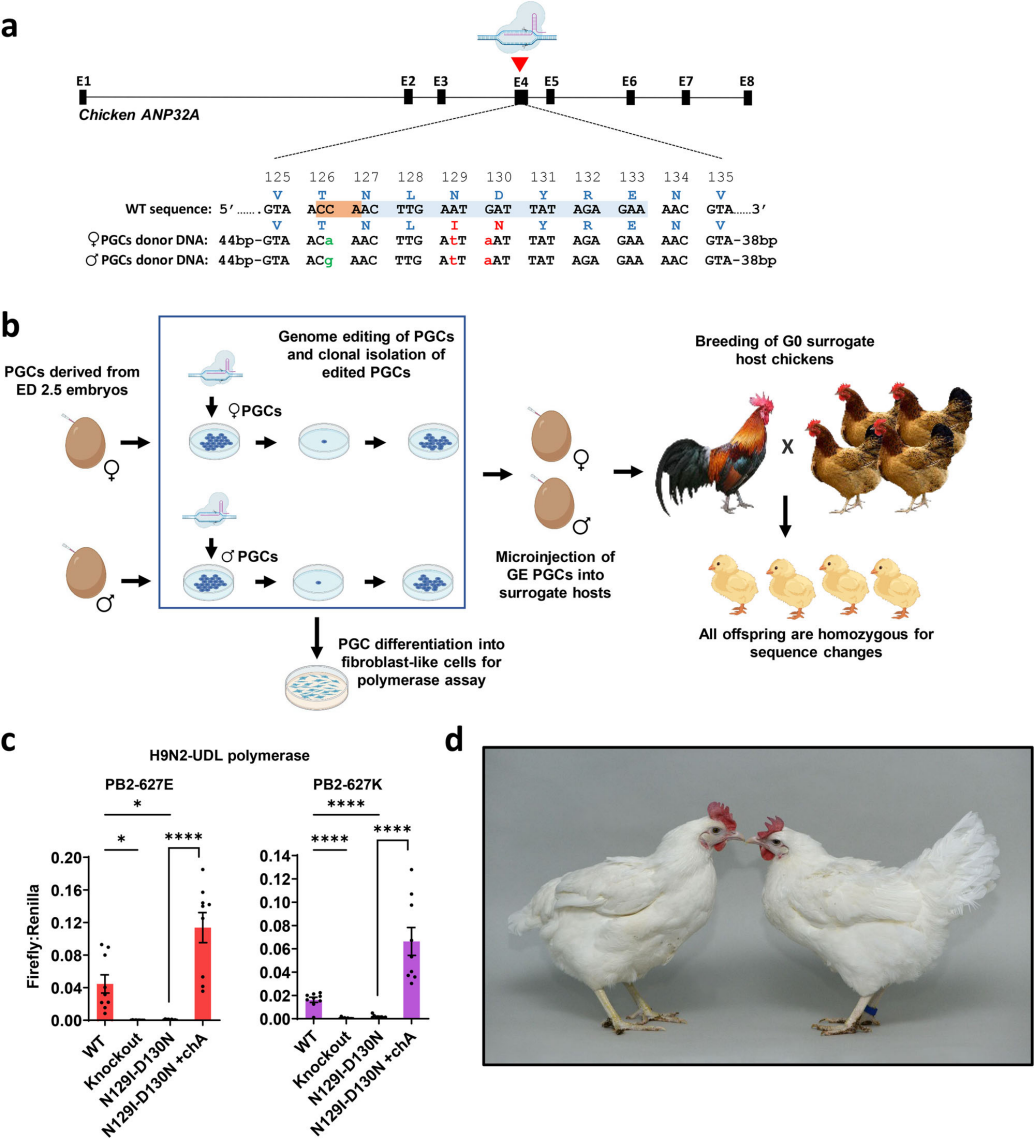
Breeding strategy for homozygous ANP32A^N129I-D130N^ chicken. **a** ANP32A editing strategy: two nucleotide changes (red letters) introduce asparagine (N) position 129 (N129I) and aspartic acid (D) position 130 (D130N) missense mutations. The third nucleotide change (green letters) is a synonymous mutation in the gRNA PAM and serves as a marker control for allelic contribution from the male and female surrogate hosts. **b** Male and female PGC cultures were derived from the blood of individual chick embryos. The PGCs were edited, and clonal lines of GE PGCs were propagated and analysed. GE PGCs were differentiated into fibroblast-like cells for IAV polymerase assays. To generate GE chicks, GE PGCs were mixed with B/B dimerisation compound (to induce cell death of host embryo germ cells) and injected into iCaspase9 host embryos, which were incubated to hatch. After hatching, the surrogate hosts were raised to sexual maturity and directly mated. All offspring from eggs laid by the surrogate hosts were biallelic for the edit and contained the parent-specific PAM nucleotide change. **c** the activity of reconstituted IAV polymerase was assessed in fibroblast-like cells derived from ANP32A^knockout^ (Knockout), ANP32A^N129I-D130N^ (N129I-D130N) or wild-type (WT) PGCs. Cells were transfected with avian IAV polymerase (PB2/627E - black bars) or human-adapted isoforms (PB2/627K - grey bars), Firefly minigenome reporter and Renilla reporter control plasmids and then incubated at 37 °C for 48 h. Wild-type chicken ANP32A (chA) cDNA was co-expressed with minigenome plasmids to rescue polymerase activity in ANP32A^N129I-D130N^ cells. Data shown are Firefly activity normalised to Renilla plotted as mean ± SEM derived from (*n* = 3) three independent experiments each consisting of three technical replicates. Error bars represent standard error of mean (SEM). One-way ANOVA and Dunnett’s multiple comparison test were used to compare polymerase activity in the GE cells with polymerase activity in WT cells. Unpaired two-tailed t-test was used to compare ANP32A^N129I-D130N^ and ANP32A^N129I-D130N^ +chA data. Statistical annotations are defined as *P ≤ 0.05, ****P ≤ 0.0001. **d** Image: wild-type (WT) hen (left) and homozygous ANP32A^N129I-D130N^GE hen (right, blue ring on right shank).

To generate ANP32A-GE (ANP32A^N129I-D130N^) cells and chickens, we applied CRISPR/Cas9 and a short single-stranded oligonucleotide (ssODN) template to introduce a 3-nucleotide base-pair change in exon 4 of ANP32A creating a two-amino-acid substitution (Fig. 1a)^14,15^. We targeted the locus in in vitro propagated male and female chicken primordial germ cells (PGCs) and then cultured single PGCs to establish clonal GE cell lines (Fig. 1b and Supplementary Fig. 1). Sanger sequencing of clonal cells identified cells containing biallelic edits. We examined the top predicted CRISPR/Cas9 off-target sites and detected no off-target mutations in selected GE clonal lines (Supplementary Fig. 2). We also confirmed that ANP32A protein expression levels were not altered in the GE PGC lines using western blot analysis (Supplementary Fig. 3). Principal component analysis (PCA) and heat maps generated from an RNA transcriptome analysis showed that the PGCs cluster by sex and cell line rather than the ANP32A genotype of the cells, indicative of no significant changes in the transcriptome of the edited cells (Supplementary Figs. 4 and 5).

### IAV polymerase activity is restricted in ANP32A^N129I-D130N^ edited chicken cells

We, and others, have previously shown that exogenously expressed chicken ANP32A variants containing either the N129I or D130N amino acid substitutions fail to complement avian influenza polymerase function in human cells or in chicken cells that lack wild-type ANP32A^10, 13^. Here, we directly assessed IAV polymerase activity in the ANP32A^N129I-D130N^ edited chicken cells. We differentiated PGC lines harbouring the ANP32A^N129I-D130N^ edit, or an edit designed to abrogate ANP32A expression by a small deletion in exon 1 (ANP32A^knockout^) or WT PGCs into fibroblast-like cells, which are permissive for IAV replication and minigenome polymerase assays^10^, and assessed the activity of the reconstituted avian IAV polymerase (PB2 627E) or the human-adapted isoform (PB2 627 K) from three avian influenza A viruses: H9N2-UDL virus (A/chicken/Pakistan/UDL-1/2008, a low pathogenic virus of the predominant G1 lineage), H5N1 50-92 virus (A/turkey/England/50-92/1991, representing a highly pathogenic avian influenza virus with no record of zoonosis) and H5N1 Tky05 virus (A/turkey/Turkey/1/2005, a highly pathogenic avian virus that has frequently infected humans). All IAV polymerases were active in wild-type cells but inactive in either ANP32A^N129I-D130N^ or ANP32A^knockout^ cells (Fig. 1c and Supplementary Fig. 6); exogenous expression of wild-type chicken ANP32A rescued polymerase activity in ANP32A^N129I-D130N^ cells. These results confirm that the residues at position 129 and 130 of chicken ANP32A are key determinants of IAV polymerase activity in chicken cells.

### Generation of ANP32A^N129I-D130N^ GE chickens using sterile surrogate hosts

We generated surrogate host chickens producing only gametes that harbour the ANP32A^N129I-D130N^ edit through microinjection of male and female GE PGCs into iCaspase9 sterile host embryos (Fig. 1a, Supplementary Fig. 1 and Supplementary Table 1)^16^. Subsequent mating of the mature surrogate host male and female chickens (Sire Dam Surrogate mating) generated homozygous ANP32A^N129I-D130N^ eggs and chicks for analysis in a single generation (Supplementary Table 2). As ANP32A function is associated with the development of bone, cartilage, brain and heart in mouse models, we monitored the growth of ANP32A^N129I-D130N^ chick embryos to identify any health or developmental defects^17,18^. We observed no developmental abnormalities in ANP32A^N129I-D130N^ embryos and proceeded to hatch a small cohort of GE chicks consisting of seven ANP32A^N129I-D130N^ chicks (generation G_1_ comprising 4 females and 3 males). In this preliminary experiment, we did not observe any differences in growth, external appearance, behaviour, or vaccination response in comparison to wild-type controls (Fig. 1d and Supplementary Figs. 7–9). All four ANP32A^N129I-D130N^ hens began laying at 20 weeks of age and subsequent egg production was comparable to wild-type hens (5.75 eggs per hen/week measured over a 2-month period).

### ANP32A^N129I-D130N^ chickens are resistant to low-dose IAV infection

**Fig. 2:**
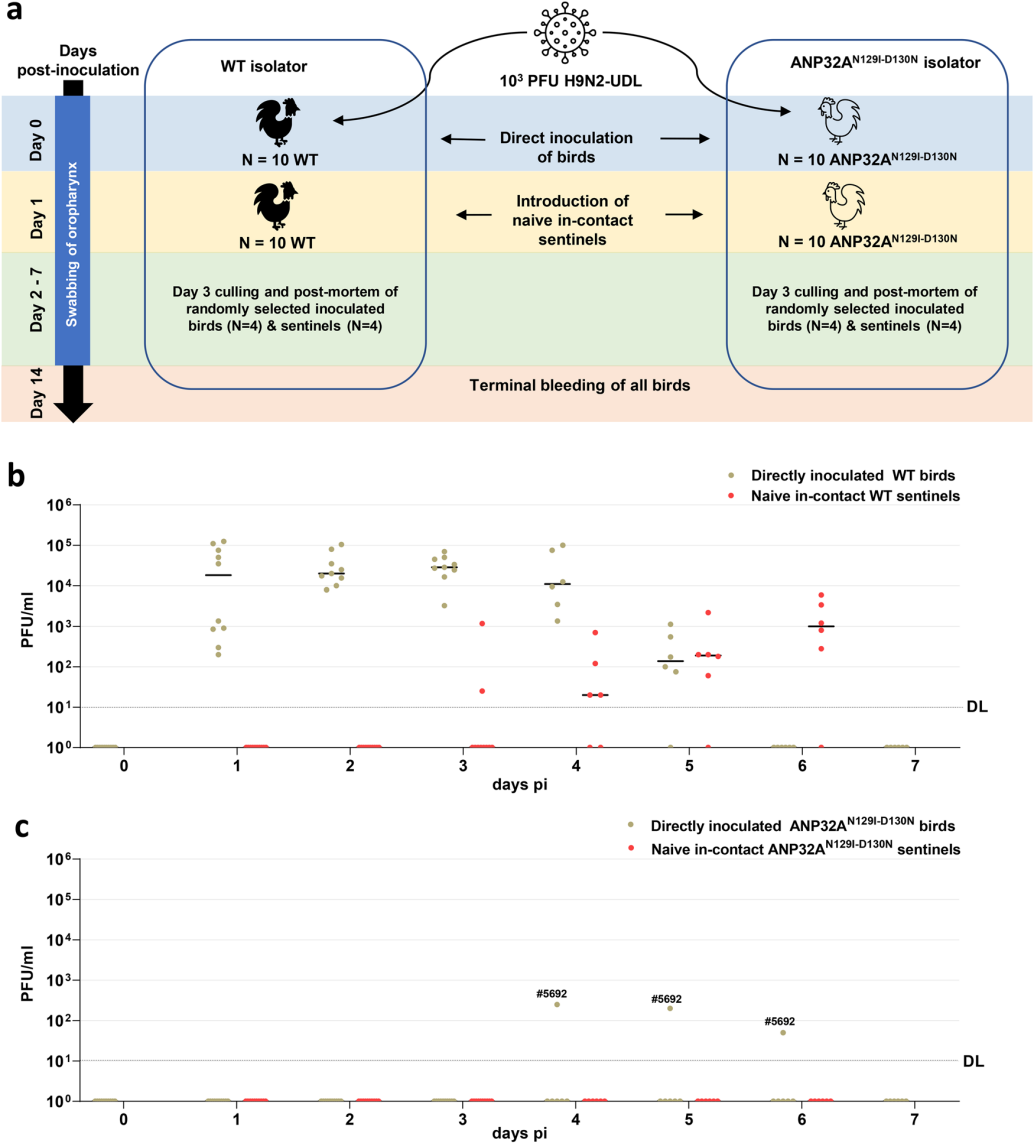
Assessment of low-dose IAV infection in ANP32A^N129I-D130N^ chickens. **a** Schematic of low-dose in vivo challenge of 2-week-old chickens with H9N2-UDL influenza A virus (A/chicken/Pakistan/UDL01/08). Chickens were housed in negative pressure poultry isolators. Prior to challenge all birds were bled from the wing vein to obtain pre-infection sera. Groups of ten WT (black) chickens or ten ANP32A^N129I-D130N^ (white) chickens were intranasally inoculated with 1 × 10^3^ PFU of H9N2-UDL virus per bird. Uninoculated sentinel chickens were introduced into the isolators 24 h post infection to assess for transmission from the directly inoculated birds. Oropharyngeal cavities of each bird were swabbed daily from the day of inoculation (D0) until day 7 (D7) post-inoculation. Infectious virus titre in swabs was measured by plaque assay on MDCK cells (**b**, **c**). **c** Bird ID number for directly inoculated ANP32A^N129I-D130N^ birds above the detection limit is indicated. DL detection limit of 10 PFU/ml for plaque assay.

To assess the susceptibility of the GE chickens to IAV infection and transmission, 10 wild-type (WT) and 10 ANP32A^N129I-D130N^ 2-week-old chickens were intranasally inoculated with 10^3^ PFU of the low pathogenic avian influenza H9N2-UDL virus in separate isolators. (Fig. 2a). In all, 24 h post-inoculation, 10 naive WT sentinel birds were introduced into the WT isolator and 10 naive GE sentinel birds were introduced into the ANP32A^N129I-D130N^ isolator to assess onward virus transmission. 4 directly inoculated birds and 4 sentinel birds were killed from each isolator on day 3 post-inoculation (pi) for post-mortem examination. Throughout the 14-day observation period, no clinical signs were observed in any birds and post-mortem analysis revealed no pathological lesions.

As the H9N2-UDL virus used in the challenge replicates predominantly in the respiratory tract of chickens, oropharyngeal swabs were collected from day 0 to day 7 pi to assess the shedding of infectious virus by plaque assay^19^. Infectious virus was detected consistently in all directly inoculated WT birds (10 of 10 birds) from day 1 pi until day 4 pi with daily mean titres above 3 × 10^4^ PFU/ml, after which titres reduced and virus was cleared by day 6 pi (Fig. 2b). 7 of 10 co-housed WT sentinel birds acquired infection by the direct exposure route from the directly inoculated birds and shed virus from day 3 to day 6 post exposure (pe) (Fig. 2b).

In contrast to the WT birds, oropharyngeal shedding of infectious virus was not detected in 9 of 10 directly inoculated ANP32A^N129I-D130N^ birds. One bird (#5692) showed delayed shedding from day 4 pi to day 6 pi with low viral titres of 250 PFU/ml, 200 PFU/ml, and 50 PFU/ml respectively (Fig. 2c). None of the co-housed ANP32A^N129I-D130N^ sentinel birds were infected (Fig. 2c), suggesting overall that the ANP32A^N129I-D130N^ genotype confers resistance to naturally shed doses of IAV virus.

To serologically confirm virus infection, blood samples were collected from all remaining birds on day 14 pi and analysed using the haemagglutination-inhibition (HI) assay to detect antibodies to the H9N2 IAV. All directly inoculated WT birds (6 of 6 birds) seroconverted with HI titres ranging from 128 to 2048 HI units (Supplementary Fig. 10a). All WT sentinel birds (6 of 6 birds) seroconverted and had HI titres equal or >128 HI units, confirming extensive virus transmission in the WT isolator. In contrast, the directly inoculated ANP32A^N129I-D130N^ birds (5 of 6 birds) for which shedding was not observed did not seroconvert. Furthermore, the antibody titre in the single positive directly inoculated ANP32A^N129I-D130N^ bird (#5692) was 64 HI units which corroborates the low level of oropharyngeal virus shedding observed in this bird. None of the ANP32A^N129I-D130N^ sentinel birds (6 of 6 birds) seroconverted (Supplementary Fig. 10b).

### ANP32A^N129I-D130N^ chickens display resilience to high-dose IAV infection

**Fig. 3:**
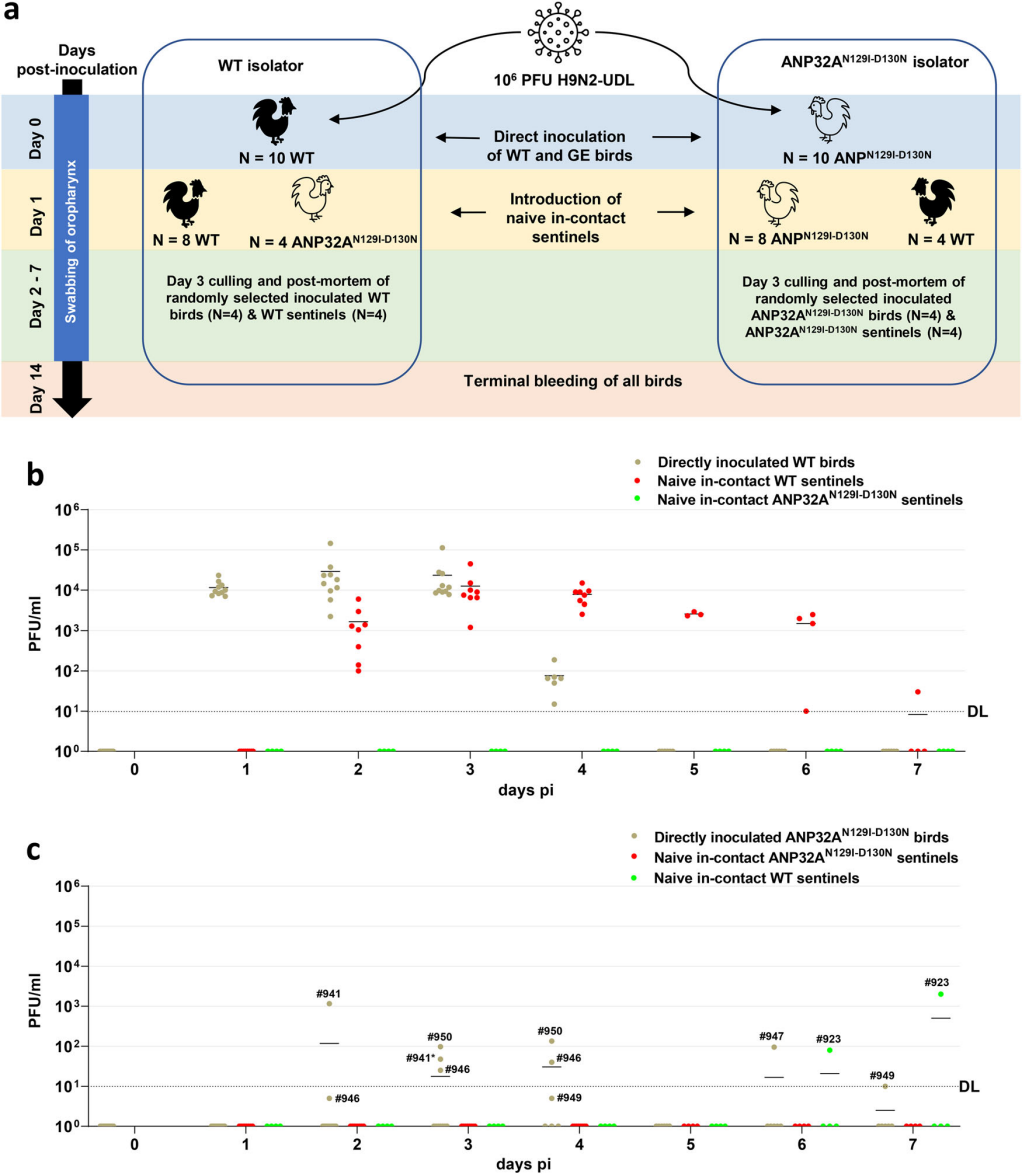
Assessment of high-dose IAV infection in ANP32A^N129I-D130N^ chickens. **a** Schematic of high-dose in vivo challenge of 2-week-old chickens with H9N2-UDL influenza A virus (A/chicken/Pakistan/UDL01/08). Chickens were housed in negative pressure poultry isolators. Prior to challenge all birds were bled from the wing vein to obtain pre-infection sera. Groups of ten WT (black) chickens or ten ANP32A^N129I-D130N^ (white) chickens were intranasally inoculated with 1 × 10^6^ PFU of H9N2-UDL virus per bird. Uninoculated naive sentinel chickens were introduced into the isolators 24 h post challenge (day 1 pi) to assess for transmission from the directly inoculated birds. Oropharyngeal cavities of each bird were swabbed daily from the day of inoculation (day 0) until day 7 post inoculation. Infectious virus titre in swabs was measured by plaque assay (**b**, **c**). **c** Bird ID number for plaque-positive directly inoculated ANP32A^N129I-D130N^ birds are indicated. Bird #941 was one of four directly inoculated ANP32A^N129I-D130N^ birds culled on day 3 pi for post-mortem examination. DL detection limit of 10 PFU/ml for plaque assay.

We next assessed susceptibility and transmission following challenge with a higher dose of IAV. 10 WT and 10 ANP32A^N129I-D130N^ 2-week-old chickens were intranasally inoculated with 10^6^ PFU of H9N2-UDL virus per bird (Fig. 3a), 1000x the dose used in the first experiment. Sentinel chickens of both genotypes were introduced into each isolator 24 h post-inoculation to assess onward virus transmission (Fig. 3a). 4 ANP32A^N129I-D130N^ and 8 WT sentinel birds were introduced into the WT isolator, while 4 WT and 8 ANP32A^N129I-D130N^ birds were introduced into the ANP32A^N129I-D130N^ isolator. 4 directly inoculated birds from each isolator and 4 sentinel birds of the same genotype were killed on day 3 pi or pe respectively for post-mortem examination. Despite the higher dose used for inoculation, no clinical signs or post-mortem pathological lesions were observed in any of the birds.

All WT birds were robustly infected and transmitted virus to all WT sentinels (Fig. 3b). Infectious virus was detected in swabs from directly inoculated WT chickens from day 1 pi until day 4 pi, peaking on day 2 pi, with a mean titre of 2.9 × 10^4^ PFU/ml (Fig. 3b). All WT sentinel birds acquired infection and shed high titres of infectious virus (mean peak titre of 1.3 × 10^4^ PFU/ml on day 2 pe)) from day 1 pe until day 5 or 6 pe (Fig. 3b). In contrast, none of the 4 ANP32A^N129I-D130N^ birds housed in the WT isolator became infected suggesting they were resistant to infection by a naturally transmitted dose (Fig. 3b).

Low-level and sporadic oropharyngeal shedding of infectious virus was observed in 5 of the 10 directly inoculated ANP32A^N129I-D130N^ birds between day 2 pi and day 7 pi (Fig. 3c). 3 of the 10 directly inoculated ANP32A^N129I-D130N^ birds (#941, #946 and #950) shed virus on day 2 and day 3 pi after which 4 birds, including bird #941, were randomly culled (Fig. 3c). Oropharyngeal shedding was subsequently observed in 2 other GE birds (#947 and #949) between day 4 and day 7 pi (Fig. 3c). The daily shed virus titres in infected directly inoculated ANP32A^N129I-D130N^ birds were below 150 PFU/ml and generally more than 2 logs lower than that observed in WT birds except in a single bird (#941) which had a titre of 1.2 × 10^3^ PFU/ml on day 2 pi. The median duration of infectious virus shedding by the 5 infected directly inoculated ANP32A^N129I-D130N^ birds was 2 days compared to 4 days for the WT birds (Supplementary Fig. 11). The area-under-curve (AUC) of shed infectious virus was significantly reduced (*p* = 0.0018, two-tailed T-test) for directly inoculated ANP32A^N129I-D130N^ birds (185.2 PFU/ml ± 264.7 PFU/ml SEM) compared to WT birds (5.9 × 10^4^ PFU/ml ± 3.7 × 10^4^ PFU/ml SEM) (Supplementary Fig. 12).

Sporadic and low-level virus shedding from directly inoculated ANP32A^N129I-D130N^ birds resulted in virus transmission to a single (1 of 4 birds) WT sentinel bird (bird #923), in which oropharyngeal virus shedding was detected on day 5 pe and day 6 pe (80 PFU/ml and 2000 PFU/ml, respectively) (Fig. 3c). None of 8 ANP32A^N129I-D130N^ sentinel birds exposed to directly inoculated ANP32A^N129I-D130N^ birds were infected, demonstrating lack of transmission between ANP32A^N129I-D130N^ birds (Fig. 3c).

Serology confirmed all infections that had been detected virologically. All directly inoculated WT birds and WT sentinel birds housed in the WT isolator seroconverted to the H9N2-UDL virus with HI assay titres ranging from 128 to 4096 HI units (Supplementary Fig. 13). In contrast, the HI assay titres for 3 of the 4 ANP32A^N129I-D130N^ sentinels in the WT isolator was below the assay detection limit of 5 HI units, while the fourth ANP32A^N129I-D130N^ sentinel had a low antibody titre of 32 HI units, however, infectious virus was not isolated from this bird. HI titres in the directly inoculated ANP32A^N129I-D130N^ birds (ranging from 4 to 256 HI units) were significantly lower than in directly inoculated WT birds (ranging from 256 to 4096 HI units), reflecting a lower level of virus replication in these birds (Supplementary Fig. 13). Antibodies were not detected in sera from any of the 4 ANP32A^N129I-D130N^ sentinels nor 3 of 4 WT sentinels in the ANP32A^N129I-D130N^ isolator. The single infected WT sentinel bird (bird #923) in the ANP32A^N129I-D130N^ isolator had a HI titre of 256 HI units.

Overall, these results demonstrate that following inoculation with high titres of the H9N2-UDL virus, the ANP32A^N129I-D130N^ genotype suppresses viral infection and significantly limits onward viral transmission to naive in-contact birds.

### Escape viruses contain mutations in their polymerase genes enabling support of virus replication by the edited ANP32A protein

To determine whether any adaptive mutations had occurred during viral infection in ANP32A^N129I-D130N^ chickens, oropharyngeal swabs were inoculated into WT chicken eggs to amplify sufficient viral material for sequencing. We sequenced viruses isolated from the 6 directly inoculated and infected ANP32A^N129I-D130N^ chickens (birds #5692, #941, #946, #947, #949 and #950) and the single WT sentinel chicken (bird #923) that acquired infection from the infected GE birds. Comparative sequence analysis showed the presence of different constellations of non-synonymous changes in the polymerase genes and the NS gene that were not present in the isolates from wild-type birds and were not detected in the virus inoculum (Table 1). In all birds with breakthrough infections, mutations PA-E349K, PA-T639I or PB2-M631L were detected, sometimes in combination. In addition, various NS gene mutations and PB1 mutations were detected in isolates from later time points pi from 3 birds, always in combination with PA and/or PB2 mutations.

**Table 1:** Amino acid substitutions at consensus level in viral isolates recovered from swabs of the oropharyngeal cavity of ANP32A^N129I-D130N^ chickens and single infected WT sentinel chicken

Inoculation route	Genotype	Bird #	Day	PB2	PB1	PA	NS1/NEP
Low dose - direct inoculation	WT	505	D3	–	–	–	–
Low dose - direct inoculation	ANP32A^N129I-D130N^	5692	D3	S489P	–	T639I	–
Low dose - direct inoculation	ANP32A^N129I-D130N^	5692	D6	G74S (43%) S489P (fixed)	I517L (17%)	**E349K (20%)** T639I (75%)	L52M (34%)
High dose - direct inoculation	ANP32A^N129I-D130N^	941	D2	**M631L (65%)**	–	T639I (24%)	–
High dose - direct inoculation	ANP32A^N129I-D130N^	941	D3	**M631L (87%)**	–	**E349K (72%)**	–
High dose - direct inoculation	ANP32A^N129I-D130N^	946	D2	–	–	–	–
High dose - direct inoculation	ANP32A^N129I-D130N^	946	D3	–	–	–	–
High dose - direct inoculation	ANP32A^N129I-D130N^	946	D4	–	–	**E349K (87%)**	G168V / D11Y (87%)
High dose - direct inoculation	ANP32A^N129I-D130N^	947	D6	**M631L**	–	–	–
High dose - direct inoculation	ANP32A^N129I-D130N^	949	D2	–	–	Q556R (96%)	–
High dose - direct inoculation	ANP32A^N129I-D130N^	949	D3	**M631L**	–	Q556R (10%)	G45R
High dose - direct inoculation	ANP32A^N129I-D130N^	949	D4	**M631L (74%)**	–	Q556R (23%)	G45R (74%)
High dose - direct inoculation	ANP32A^N129I-D130N^	950	D2	–	–	**E349K (35%)**	–
High dose - direct inoculation	ANP32A^N129I-D130N^	950	D3	**M631L (15%)**	–	**E349K (70%)**	–
High dose - direct inoculation	ANP32A^N129I-D130N^	950	D4	I570L (30%) **M631L (36%)**	K578T (15%)	**E349K (64%)** S409I (64%)	G211E / D54N (35%)
High dose - naïve contact	WT	923	D6	–	–	**E349K (poly)**	–
High dose - naïve contact	WT	923	D7	**M631L**	–	L345F (poly)	–

Substitutions in bold were found in almost all breakthrough infections.

**Fig. 4:**
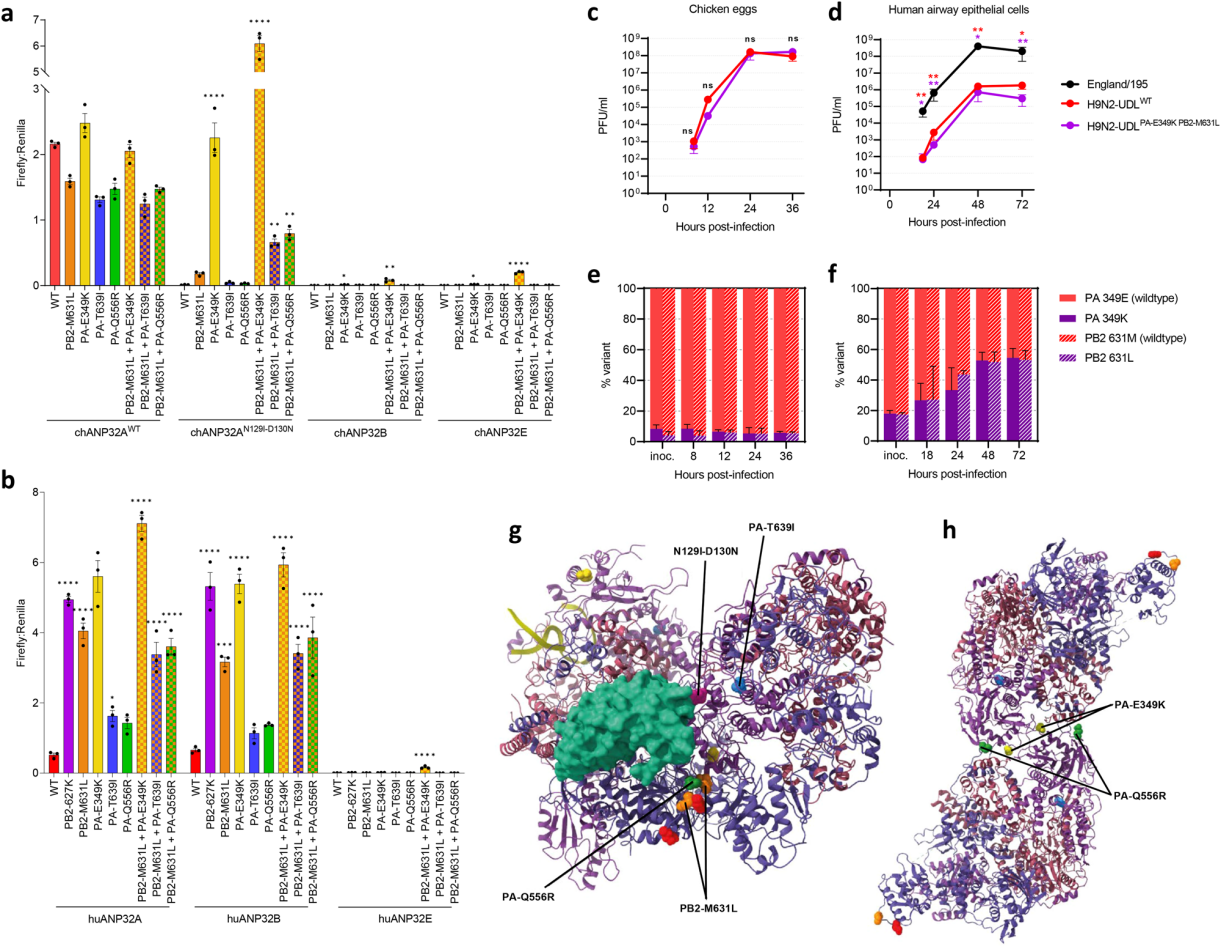
Assessment of mutations identified in polymerase genes of viruses isolated from infected ANP32A^N129I-D130N^ chickens. **a**, **b** Influenza A virus (H9N2-UDL) polymerase harbouring single or combinations of PA and PB2 mutations detected in virus isolated from ANP32A^N129I-D130N^ chickens was reconstituted together with NP by plasmid transfection in eHAP1 human cells that lack ANP32 expression and complemented with chicken (**a**) or human (**b**) ANP32-FLAG proteins. Polymerase activity was measured at 24 h post-transfection by Firefly luciferase signal generated from a minireplicon and a Renilla luciferase transfection control. Data shown are Firefly activity normalised to Renilla plotted as mean ± SEM derived from three (*n* = 3) independent experiments, each consisting of three technical replicates. Error bars represent SEM. Data statistically analysed by one-way ANOVA and Dunnett’s multiple comparison test to determine polymerase constellations whose activity varied from wild-type H9N2-UDL polymerase. Statistical annotations are defined as *P ≤ 0.05, **P ≤ 0.01, ***P ≤ 0.001, ****P ≤ 0.0001. **c** WT embryonated eggs were inoculated with 100 PFU of wild-type H9N2-UDL (H9N2-UDL^WT^) virus or the double mutant variant (H9N2-UDL^PA-E349K PB2-M631L^) containing the PA-E349K and PB2-M631L mutations. The inoculated eggs were incubated at 37.5 °C. Allantoic fluids were collected at the indicated timepoints. Data are PFU/ml in allantoic fluids measured by plaque assay and statistically analysed by multiple unpaired two-sample T-test. Statistical annotations are defined as ns not significant. **d** Human airway epithelial cells were infected with human-adapted H1N1 virus (A/England/195/2009) (England/195) or H9N2-UDL^WT^ virus or H9N2-UDL^PA-E349K PB2-M631L^ and incubated at 37.0 °C. Cell culture supernatants were harvested at the indicated timepoints and titrated by plaque assays. Data was statistically analysed by multiple unpaired two-sample T-test to compare growth of England/195 virus with growth of H9N2-UDL^WT^ virus or H9N2-UDL^PA-E349K PB2-M631L^ at each timepoint. Data was analysed by one-way ANOVA and Dunnett’s multiple comparison test. Statistical annotations are defined as *P ≤ *0.05,* **P ≤ *0.01*. **e** WT embryonated eggs were inoculated with 100 PFU of a mixture of H9N2-UDL^WT^ and H9N2-UDL^PA-E349K PB2-M631L^ virus containing <10% of the mutant virus. The inoculated eggs were incubated at 37.5 °C. Allantoic fluids were collected at the indicated timepoints and followed by viral RNA purification. Next generation sequencing was performed on purified viral RNA to determine variant frequency in each egg. **f** Human airway epithelial cells were infected with a mixture of H9N2-UDL^WT^ virus and H9N2-UDL^PA-E349K PB2-M631L^ virus containing <20% of the mutant virus and incubated at 37.0 °C. Cell culture supernatants were harvested at the indicated timepoints and followed by viral RNA purification. Next generation sequencing was performed on purified viral RNA to determine variant frequency at each timepoint. **g**, **h** Location of amino acids mutated in virus isolated from ANP32A^N129I-D130N^ chickens in the asymmetric polymerase dimer in combination with chANP32A (PDB:6XZP) (**g**) or the symmetric polymerase dimer (PDB:6QXB) were plotted using ChimeraX (**h**). **g** Influenza virus asymmetric polymerase dimer (PDB: 6XZP) showing ANP32A in light green, amino acids 129 and 130 highlighted in purple, PB2-627K in red, PB2-M631L in orange, PA-E349K in yellow, PA-Q556R in dark green and PA-T691I in blue. **h** Influenza virus symmetric polymerase (PDB: 6QX8) showing PB2-627K in red, PB2-M631L in orange, PA-E349K in yellow, PA-Q556R in dark green and PA-T691I in blue.

To assess the functional relevance of the dominant PA and PB2 mutations, we performed minigenome replication assays in ANP32A, B and E triple-knockout human cells, complementing polymerase function with expression of either wild-type chicken ANP32A (chANP32A^WT^) or the modified chicken ANP32A (chANP32A^N129I-D130N^) or the usually non-functional chicken ANP32B or E (chANP32B, chANP32E) proteins. Wildtype chicken ANP32A efficiently supported the activity of all the mutant IAV polymerases (Fig. 4a), indicating that these mutations did not diminish the interaction of wild-type ANP32A protein with the viral polymerase. As expected, chicken ANP32A^N129I-D130N^ did not support the activity of the wild-type polymerase. However, chicken ANP32A^N129I-D130N^ supported robust activity of polymerase harbouring the PA-E349K mutation alone, and when this PA mutation was combined with PB2-M631L, the polymerase activity with the edited chicken ANP32A protein was higher than with wild-type chicken ANP32A. The chicken ANP32A^N129I-D130N^ protein also supported significant activity of polymerases with the combinations of PB2-M631L together with PA-T639I or PA-Q556R. Expression of chicken ANP32B or chicken ANP32E also supported very low levels of activity from the PB2 M631L-PA E349K polymerase (Fig. 4a).

Several of the polymerase mutations, PB2-M631L, PA-E349K and PA-Q556R, have previously been reported to enhance polymerase activity and replication of mouse-adapted human and avian IAVs^20–24^. PB2-M631L, PA-E349K and PA-Q556R mutations have been detected in IAVs in bird populations and often in avian viruses that infected humans, suggesting that they are human-adaptive mutations^25^. With this in mind, we tested whether they would also enable support of polymerase function by human ANP32A or ANP32B. This was the case as PB2-M631L and PA-E349K substitutions alone or in combination were almost as potent as the common PB2 mutation, E627K, at activating support of the avian influenza polymerase by the shorter mammalian ANP32 proteins in the minigenome replication assay (Fig. 4b).

To understand the implications of the minigenome replication assay results in the context of whole virus, we generated a recombinant virus that harboured the PA-E349K and PB2-M631L mutations through reverse genetics (RG), and compared its replicative fitness with that of the isogenic RG wild-type H9N2-UDL virus in embryonated chicken eggs and in primary human airway epithelial (HAE) cells grown at air-liquid interface. We infected WT eggs with each virus and observed that viral yields were similar at 12-, 24- and 36-h timepoints (Fig. 4c). In primary cultures of HAE cells, individual growth curves also showed no difference in replication at 24-, 48- and 72-h timepoints (Fig. 4d).

Subsequently, we co-infected WT eggs with RG wild-type and double mutant PA-E349K PB2-M631L viruses to assess the competitive fitness of the mutant virus. A minority input of the double mutant virus (<10%) was used to see if it would outcompete the wild-type virus. The double mutant maintained a frequency of 5-10% throughout, demonstrating that the polymerase mutations provided neither a fitness advantage nor a defect (Fig. 4e), in line with their equal activity supported by wild-type chANP32A in the polymerase assay (Fig. 4a). However, in human airway epithelial cultures, a higher original input of <20% double mutant genomes became enriched to approximately 50% of the virus population by 48 h post-infection demonstrating a subtle replicative advantage (Fig. 4f), also in line with the enhanced support of the mutant polymerase by human ANP32A and B proteins in the polymerase assay (Fig. 4b). It is important to note that polymerase gene mutations alone are not sufficient to adapt an avian influenza virus to humans. A major species barrier for avian influenza also exists at the level of cell binding and entry that is determined by the virus haemagglutinin protein, HA. Adaptations in HA are absolutely required for efficient infection and onwards transmission in humans^1^. Thus, despite the double mutant H9N2-UDL virus demonstrating a minor fitness advantage over wild-type virus in the HAE competition model, its replication was still 2–3 orders of magnitude lower in human airway cells than that of a human-adapted virus, A/England/195/2009(H1N1), at all timepoints in head-to-head growth kinetics (Fig. 4d).

### Most polymerase mutations that arose in ANP32A^N129I-D130N^ edited birds are distal to the interface with ANP32 amino acids 129 and 130 in the polymerase/ANP32 complex structure

To assess the structural context of the mutations identified in viral isolates from breakthrough infections, we mapped the location of the substituted amino acids in the polymerase subunits to the published structure of the asymmetric influenza C virus (ICV) polymerase dimer in complex with chicken ANP32A^12^ (Fig. 4g). Chicken ANP32A residues N129 and D130 sit on the edge of the fifth leucine-rich repeat domain (LRR5) of ANP32A. One of the polymerase substitutions, PA-T639I, is located in a region of PA in the encapsidating polymerase opposite LRR5 but is not a contact residue with amino acids 129 or 130 in the ICV complex. Another of the PA substitutions, at amino acid 556 is also situated in the encapsidating polymerase in the vicinity of the central region of ANP32A essential for supporting polymerase activity^10,26^. The PB2 residue 631 is located close to the prototypic host-range determining residue 627 and within the PB2-627 domain which is thought to interact with the unstructured LCAR of ANP32A^27^. However, neither of these PA substitutions or PB2 M631L on their own enabled use of the edited ANP32A^N129I-D130N^ (Fig. 4a).

In contrast, the PA substitution E349K, which had the largest effect on polymerase activity, is not located in any polymerase region interacting with the host ANP32A protein in the solved asymmetric dimer complex (Fig. 4g)^12^. However, influenza virus polymerase also forms an alternative symmetric dimer required for the replication of the vRNA genome from a cRNA template, a part of the replication cycle functionally associated with ANP32 proteins, although currently no structure of this dimer in complex with the host protein exists^28,29^. Interestingly, three PA amino acids associated with the breakthrough viruses, 345, 349 and 556, are located on the interface of this symmetric dimer (Fig. 4h)^29,30^. How mutations that affect the symmetric dimer might compensate for suboptimal ANP32 proteins to support replication is not currently understood. Since the polymerase in infected cells exists as at least two different conformations, mutations that destabilise one might enable formation of the other even under suboptimal conditions^23^.

### The PA-349K PB2-M631L double mutant H9N2-UDL escape virus can replicate in chicken embryos lacking ANP32A

**Fig. 5:**
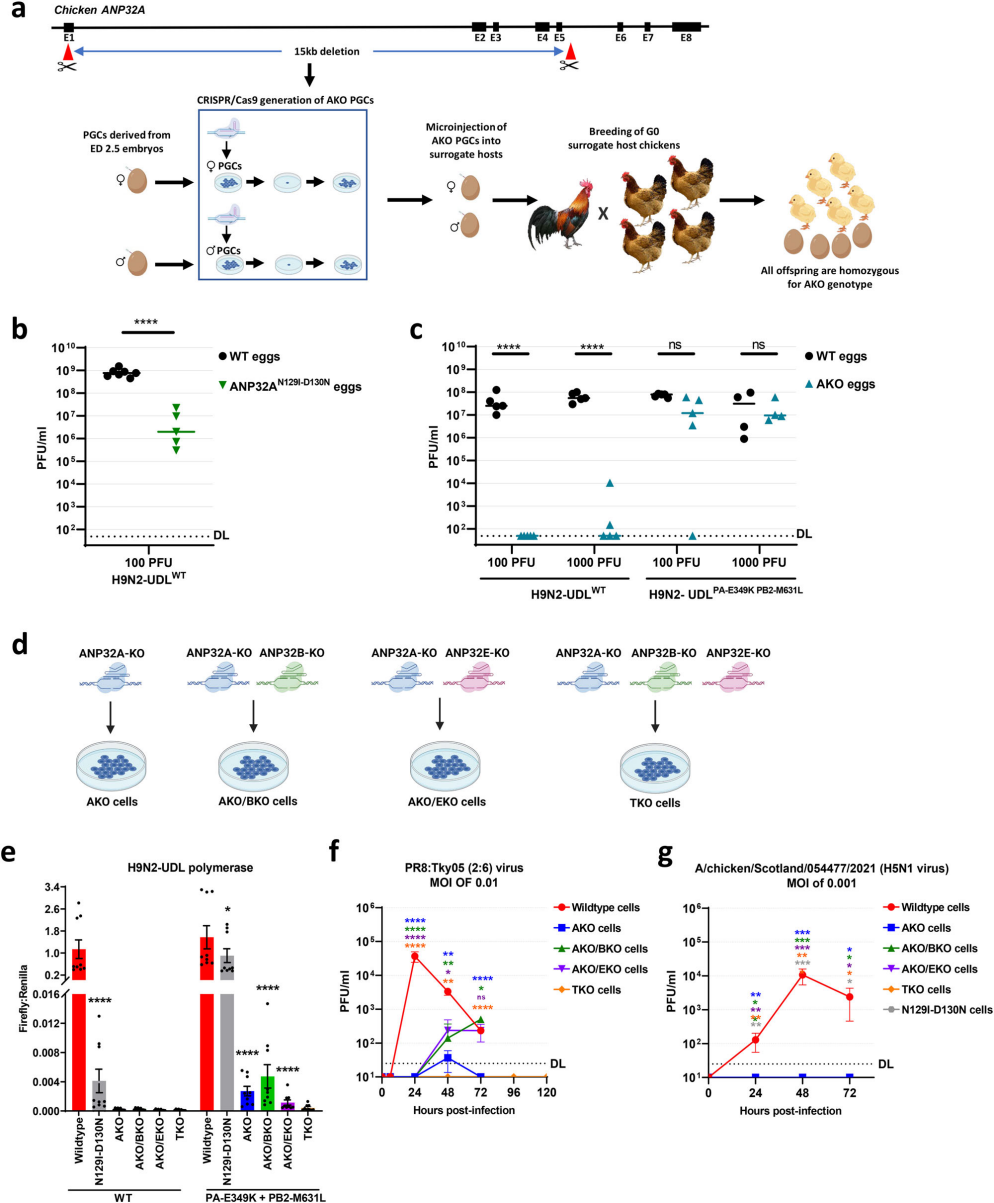
Deletion of ANP32 A, B, and E eliminates viral polymerase activity and viral proliferation. **a** ANP32A deletion strategy: two gRNAs were used to generate a 15-kb deletion in ANP32A in male and female PGCs. Clonal lines of ANP32A-knockout (AKO) PGCs were isolated, propagated and injected into iCaspase9 host embryos which were incubated to hatch. After hatching, the surrogate hosts were raised to sexual maturity and directly mated. All offspring from eggs laid by the surrogate hosts were biallelic for ANP32A deletion (see Supplementary Fig. 14). **b**, **c** WT or ANP32A^N129I-D130N^ or AKO 11-day-old embryonated eggs were inoculated with wild-type H9N2-UDL (H9N2-UDL^WT^) virus or the double mutant variant (H9N2-UDL^PA-E349K PB2-M631L^) containing the PA-E349K and PB2-M631L mutations. The inoculated eggs were incubated at 37.5 °C. Allantoic fluids were collected 48 h later and PFU/ml measured by plaque assay. DL detection limit of plaque assay (10 PFU/ml). Data were statistically analysed by unpaired two-tailed T-test of transformed data (Y = (Log(Y)). Statistical annotations are defined as ns not significant, **P ≤ 0.01, ****P ≤ 0.0001. **d** PGCs were edited to delete ANP32A, ANP32B, or ANP32E or combinations of the deletion (see Supplementary Fig. 17). PGCs were subsequently differentiated into fibroblast –like cells and used to assay polymerase activity and viral replication. **e** Wildtype (WT) H9N2-UDL polymerase or the mutant isoform harbouring PA-E349K and PB2-M631L mutations was reconstituted together with NP by plasmid transfection into chicken PGC-derived fibroblast-like cells. Polymerase activity was measured at 48 h post-transfection by detection of *Firefly* luciferase signal generated from a minireplicon normalised to a *Renilla* luciferase transfection control. Data shown are Firefly activity normalised to Renilla plotted as mean ± SEM derived from three (*n* = 3) independent experiments, each consisting of three technical replicates. Data was statistically analysed by one-way ANOVA, and Dunnett’s multiple comparison test to compare polymerase activity in wild-type cells with activity in other cell lines. Error bars are SEM. ns= not significant, *P ≤ 0.05, ****P ≤ 0.0001. Statistical annotations are defined as ns not significant, *P ≤ 0.05, ****P ≤ 0.0001. *N129I-D130N* cells refer to cells with the homozygous ANP32A^N129I-D130N^ genotype. **f**, **g** PGC-derived fibroblast-like cells were infected with a recombinant virus harbouring the HA and NA genes of the H1N1 PR8 virus, and the polymerase and other internal genes from the highly pathogenic H5N1 avian influenza virus A/turkey/Turkey/2005 (Tky05) (**f**) or a highly pathogenic H5N1 clade 2.3.4.4b virus (A/chicken/Scotland/054477/2021) (**g**). Cell culture supernatants were harvested at the indicated timepoints and titrated by plaque assays. Data was statistically analysed by one-way ANOVA and Dunnett’s multiple comparison test to compare virus growth in wild-type cells with virus growth in other cell types at each timepoint. Statistical annotations are defined as ns=not significant, *P ≤ 0.05, **P ≤ 0.01, ***P ≤ 0.001, ****P ≤ 0.0001.

Apparently, H9N2-UDL virus adapted in vivo to utilise the edited ANP32A^N129I-D130N^ protein. We asked whether complete removal of ANP32A would eliminate viral escape. We generated male and female surrogate host chickens producing gametes derived from injected PGCs containing a large loss-of-function deletion in ANP32A (AKO) (Fig. 5a, Supplementary Fig. 14)^16^. Mating of these surrogate chickens generated homozygous ANP32A-knockout (AKO) eggs and chicks in a single generation. We monitored the development of the AKO chick embryos and observed no developmental defects. We hatched a small cohort of sixteen AKO chicks (generation G1 comprising 9 females and 7 males) and did not observe any differences in external appearance, behaviour, internal anatomy, or lay rate (6.22 eggs per hen/week AKO hens; 6.71 eggsper hen/week WT hens) in comparison to wild-type (WT) controls. However, AKO chicks weighed slightly less than wild-type chicks, possibly due to a difference in initial egg size in the WT and AKO eggs (Supplementary Fig. 15).

We assessed the robustness of the AKO edit by infecting 11-day-old embryonated chicken eggs which are highly permissive to influenza infection. First, we inoculated ANP32A^N129I-D130N^ GE eggs with 100 PFU of wild-type H9N2-UDL (H9N2-UDL^WT^) virus and observed a low level of viral growth in all GE eggs (Fig. 5b). Sequencing of the virus again revealed polymerase mutations (PA-G634E, PA-K635E, PA-K635Q, PB2-G74S, PB1-F185L) in viruses isolated from the ANP32A^N129I-D130N^ GE embryonated eggs. This further confirmed that the N129I-D130N substitution in chicken ANP32A does not completely abrogate virus replication and leads to IAV escape and evolution. In contrast, infection of AKO eggs with 100 PFU of H9N2-UDL^WT^ virus led to no viral replication. Following inoculation with 1000 PFU of H9N2-UDL^WT^, 2/5 AKO eggs supported a low level of replication (Fig. 5c). In contrast, the H9N2-UDL escape double mutant virus replicated in AKO eggs following inoculation at both low (100 PFU) or high (1000 PFU) doses (Fig. 5c). This was also the case for the single E349K mutant virus and an independent sample of double mutant virus derived from a plaque pick from the original chicken isolate (Supplementary Fig. 16). Together these data indicate that even the entire deletion of chicken ANP32A is not sufficient to abrogate IAV mutant infections of chicken.

### IAV WT and mutants do not replicate in chicken cells lacking ANP32A, B, and E

We and others previously reported that neither chicken ANP32B nor chicken ANP32E supports IAV polymerase even for viruses with mammalian adapting mutations^10, 11,13^. However, we reasoned that the E349K-M631L double mutant virus that replicated in AKO eggs (Fig. 5c) may have adapted to use another member of the ANP32 protein family. Indeed, the minireplicon assay had revealed significant but very low activity supported by chANP32B or chANP32E (Fig. 4a). To investigate this possibility, we targeted chicken PGCs to generate cell lines containing concurrent loss-of-expression mutations in ANP32A and ANP32B (AKO/BKO cell line) or in ANP32A and ANP32E (AKO/EKO cell line) or in all three ANP32 proteins (TKO cell line) (Fig. 5d and Supplementary Fig. 17). Minireplicon assays indicated that the activity of the H9N2-UDL double mutant polymerase was significantly reduced but detectable in chicken cells lacking only ANP32A (AKO cells), and also in chicken cells expressing only ANP32E (AKO/BKO cells) (Fig. 5e). Importantly, polymerase activity was completely absent in TKO cells lacking all three ANP32 proteins.

Since other viral proteins such as NS1 and NEP are not expressed in minireplicon assays but have effects on polymerase activity and can compensate for defective replication, we tested whether the TKO cells remained resilient to virus replication even when these other viral products were present^31–33^. We first attempted to perform replication assays in TKO cells using the wild type and double mutant H9N2-UDL virus, however, H9N2 viral replication in in vitro cell lines was extremely low. We therefore infected the edited cells with a PR8 recombinant virus harbouring the polymerase and other internal genes from the highly pathogenic H5N1 avian influenza virus A/turkey/Turkey/1/2005 (Tky05). Virus replication was evident by 48 h post infection in wild type and all edited cells except the TKO line which yielded no infectious virus even after 120 h incubation (Fig. 5f). Finally, we infected the set of edited cell lines with a representative of the contemporary highly pathogenic H5N1 clade 2.3.4.4b viruses, and confirmed complete absence of replication in the TKO cells (Fig. 5g). Taken together, our result implies that single edits or deletions of single ANP32 proteins is not sufficient to generate influenza resistant birds but that edits of all three members of the ANP32 family will be needed.

## Discussion

Breeding for resistance and resilience to disease has significant potential in farmed poultry^34,35^. The production of transgenic chickens that expressed an RNA decoy that inhibits the IAV polymerase and prevented onward viral transmission to neighbouring birds was the first demonstration that genetic engineering could be used to introduce resistance to infectious diseases in chicken^36^. With recent advances in the development of genome editing technology, novel resistance/resilience alleles can now be introduced into chicken populations by editing host genes essential for pathogenic infections to specifically abrogate their pro-viral functions^37^.

Here we introduced a specific gene edit to the host protein ANP32A that had been shown to abrogate its support for the influenza polymerase in cell culture^10,13^. GE birds carrying this edit showed no adverse health or productivity effects and were resistant to IAV infection by a natural transmission route following exposure to other infected birds. Thus, our data show the promise of this strategy for mitigating the incursion of avian influenza into farmed poultry from wild bird sources. Even following a direct inoculation with 10^3^ infectious virus particles, only a single bird was infected and the viral titres shed were low and transient, and no onwards transmission occurred. However, following direct inoculation with a higher dose, breakthrough infection occurred in the GE birds. Influenza virus is notorious for its ability to evolve, and we detected a series of different amino acid substitutions in the viral polymerase genes of viruses isolated from the GE chickens that had enabled adaptation of the enzyme to co-opt support from the edited ANP32A protein, and also to utilise otherwise suboptimal ANP32 family members. These mutations unexpectedly allowed the usually host-restricted avian influenza polymerase to use the shorter human ANP32A and B and thus partially adapted the viral polymerase for replication in mammals. Although unintended, this consequence clearly indicates the importance of a robust genome editing strategy and subsequent appraisal that includes challenge with multiple avian influenza genotypes at non-physiological exposure levels to rule out the opportunity for adaptive viral evolution.

We further generated chickens that entirely lacked expression of ANP32A, but the wild-type virus still replicated at low levels in some of the eggs, and the mutant virus was only marginally restricted. Finally, we edited all three members of the ANP32 family to generate chicken cells lacking their expression, and found no virus polymerase activity, even of the mutant polymerase, and no breakthrough infection in these cells. This combination of knockouts is expected to be deleterious to the animals’ health, but illustrates a proof of principle that multiple edits in host genes could be combined to confer sterile resistance. Indeed, editing of the three ANP32 genes will be futile if increased resistance to Avian Influenza is accompanied by any loss in fitness of the birds; for example effects on development, weight gain or fecunidity, and/or increased susceptibility to other avian pathogens.

Future assessment of GE animals, after the research phase of their development and prior to their distribution, should take into account whether appropriate investigatory steps have been carried out to evaluate if genome-edited livestock might drive pathogen evolution. This is especially relevant for pathogens with zoonotic potential as was shown here. We suggest that a suitable strategy for generating avian influenza resistant chickens will require multiple edits that destroy the pro-viral potential of ANP32A, B and E to eliminate the likelihood that escape mutants can arise.

## Methods

### Animals

Fertile eggs were obtained from commercial Hy-line layer flocks bred at the National Avian Research Facility, Midlothian, United Kingdom. All chicken lines were bred and maintained under UK Home Office License. All experiments and procedures were performed in accordance with relevant UK Home Office regulations. Experimental protocols and studies were reviewed by the Roslin Institute Animal Welfare and Ethical Review Board (AWERB) Committee, GM and Biological Safety Committee and performed under Home Office Licence (PP9565661). All animal challenge work was approved and regulated by the UK government Home Office under the project license (P68D44CF4) and reviewed by the Pirbright Animal Welfare and Ethics Review Board (AWERB). All personnel involved in the procedures were licensed by the UK Home Office. All procedures were performed in accordance with these guidelines and the study is reported in line with the ARRIVE guidelines.

### PGC derivation and culture

PGC lines were derived from individual fertile eggs, cultured in FAOT medium and expanded to 400,000 cells in 5 weeks before performing gene editing experiments^38^. Fertile eggs bred from Hy-line layer lines were incubated for 2.5 days and then 1 μl of embryonic blood was taken from the dorsal aorta of HH stage 16 HH embryos and placed into FAOT medium. FAOT medium contains custom-made Avian Knockout DMEM (Life Technologies #041-96570 M) 1× B-27 supplement (Life Technologies #17504044), 2.0 mM GlutaMax (Life Technologies #35050-038), 1× non-essential amino acids (Life Technologies #11140050), 1× EmbryoMax nucleosides (Merck Millipore #ES-008-D), 0.1 mM β-mercaptoethanol (Life Technologies #31350010), 0.2% ovalbumin (Sigma-Aldrich A5503), 1.2 mM sodium pyruvate (Life Technologies #11360070), 0.15 mM CaCl2, 0.01% sodium heparin, 4 ng/ml h-FGF2 (R&D Systems), 50 ng/ml ovotransferrin (Sigma-Aldrich C7786) and 25 ng/ml activin A (Peprotech). PGCs were grown at 37 °C in a 5% CO_2_ atmosphere and fed every 48 h.

### CRISPR Plasmids and ssODN donor

PX458-mcherry, PX458-GFP, PX459 V2.0 and HF-PX459 V 2.0 vectors were used for expression of CRISPR/Cas9^14,15,39^. PX458-mcherry was a gift from Joanna Wysocka (Addgene plasmid # 161974). PX458 was a gift from Feng Zhang (Addgene plasmid # 48138). PX459 V2.0 vector was a gift from Feng Zhang (Addgene plasmid # 62988). gRNA sequences were selected using CHOPCHOP gRNA design web tool (http://chopchop.cbu.uib.no/) which also generated potential off-target sites for the selected gRNA^40^. gRNA oligonucleotides were synthesised by Invitrogen and inserted into PX459 V2.0 and HF-PX459 V2.0 vectors using methods previously described^14^. Short single-stranded oligonucleotide DNA (ssODN) donor was an Ultramer® DNA Oligonucleotide synthesized by Integrated DNA Technologies (IDT). gRNA and ssODN sequences are listed in Supplementary Table 3.

### PGC gene editing and DNA sequencing

To generate ANP32A^N129I-D130N^ cells, PGCs were transiently transfected with 1.5 µg of PX459 V2.0 CRISPR/Cas9 vector and 10 µM of ssODN donor using Lipofectamine 2000 (Life Technologies) to target exon 4 of ANP32A. This was followed by treatment with 0.1 mg/ml puromycin (Sigma-Aldrich #P7255) to enrich for transfected cells^15^. To generate ANP32A^knockout^ cells, chicken PGCs were transiently transfected with 2.0 µg of PX459 V2.0 CRISPR/Cas9 vector using Lipofectamine 2000 transfection reagent to target exon 1 of ANP32A^10^. To generate the AKO genotype containing a 15-kb loss-of-function deletion in ANP32A, chicken PGCs were transiently transfected with 1.5 µg of PX459 V2.0 CRISPR/Cas9 vector to target exon 1 and intron 5 of ANP32A and followed by treatment with 0.1 mg/ml puromycin (Sigma-Aldrich #P7255) to enrich for transfected cells^15^. Single cell cultures of puromycin-selected cells were subsequently established to isolate clonal populations of homozygous gene-edited PGCs for downstream experiments as described previously^15^. Briefly, PGCs were seeded at 1 cell per well in 110 µl FAOT medium in 96-well plates using a FACS Aria III machine (BD Biosciences) and subsequently cultured for 2 to 3 weeks when cell density will reach 30 to 50%. The PGC cultures were then transferred to 48-well plates and subsequently into 24-well plates for further expansion for downstream experiments. Alternatively to generate the AKO genotype, PGCs were transiently transfected with 1.5 µg each of PX458-GFP and PX458-mcherry CRISPR/Cas9 vectors to target exon 1 and intron 5 of ANP32A and followed by fluorescence-activated cell sorting (FACS) 48 h later to establish single cell cultures to isolate clonal populations of homozygous gene-edited PGCs. To generate the BKO genotype containing a 134-bp loss-of-function deletion in ANP32B, PGCs were transiently transfected with 1.5 µg each of PX458-GFP and PX458-mcherry CRISPR/Cas9 vectors to target the promoter region and exon 1 of ANP32B, followed by fluorescence-activated cell sorting (FACS) 48 h later to establish single cell cultures to isolate clonal populations of homozygous gene-edited PGCs. To generate the EKO genotype containing a 160-bp loss-of-function deletion in ANP32E, PGCs were transiently transfected with 1.5 µg each of PX458-GFP and PX458-mcherry CRISPR/Cas9 vectors to target exon 2 and intron 2 of ANP32E, followed by fluorescence-activated cell sorting (FACS) 48 h later to establish single cell cultures to isolate clonal populations of homozygous gene-edited PGCs. To generate the AKO/BKO (containing concurrent loss-of-function deletions in ANP32A and ANP32B) and AKO/EKO genotypes (containing concurrent loss-of-function deletions in ANP32A and ANP32E), BKO PGCs and EKO PGCs were respectively transiently transfected with 1.5 µg each of PX458-GFP and PX458-mcherry CRISPR/Cas9 vectors to target exon 1 and intron 6 of ANP32A and followed by fluorescence-activated cell sorting (FACS) 48 h later to establish single cell cultures to isolate clonal populations of homozygous gene-edited PGCs. To generate the TKO genotype (containing concurrent loss-of-function deletions in ANP32A, ANP32B and ANP32E), AKO/BKO PGCs were transiently transfected with 1.5 µg each of PX458-GFP and PX458-mcherry CRISPR/Cas9 vectors to target exon 2 and intron 2 of ANP32E, followed by fluorescence-activated cell sorting (FACS) 48 h later to establish single cell cultures to isolate clonal populations of homozygous gene-edited PGCs.

To screen for ANP32A^N129I-D130N^ cells, single-cell clones were analysed by PCR amplification of genomic DNA and Sanger sequencing using primers (5′–AGAGGAAGGGAGCAAAAGTCA–3′, 5′–ATGCTTGTCTTCCTCCTTCCA–3′). To screen for ANP32A^knockout^ cells containing targeting of exon 1 only, single cell clones were analysed by PCR amplification of genomic DNA and then cloning of the PCR products into pGEM-T Easy vector (Promega), followed by Sanger sequencing using T7 promoter forward primer. To screen for the AKO genotype, single-cell clones were analysed by PCR amplification of genomic DNA and Sanger sequencing using primers (5′–TCAAAGTCCCTTATTACCGCG–3′, 5′–CCTTTCACTCCCCATCTTTCA–3′) that bind to areas outside the deleted 15-kb region and amplify a PCR product of approximately 220 bp only if the deletion is successful but will fail to yield a product if there is no deletion. To screen for the BKO genotype, single-cell clones were analysed by PCR amplification of genomic DNA and Sanger sequencing using primers (5′–GGTGCCATTTTGTCGAGGG–3′, 5′–CTCTCCAGGCTTCTTGTTGC–3′) overlapping the deleted region. To screen for the EKO genotype, single-cell clones were analysed by PCR amplification of genomic DNA and Sanger sequencing using primers (5′–ATGTCATGGAGGCGCAGT–3′, 5′–CCCCAAATCAGTAAAAGCCCC–3′) overlapping the deleted region. PCR primers used for amplification of selected off-target sites are detailed in Supplementary Table 4. PCR gel electrophoresis data was collected using the NuGenius Gel documentation system (Syngene). Sequencing data was viewed and analysed using SeqMan Pro 17 and MegAlign Pro 17 (Lasergene 17, DNASTAR) or SnapGene Version 6.2.1(Dotmatics).

### PGC transcriptome analysis

In all, 200,000 PGCs were expanded in FAOT culture medium to 2 × 10^6^ cells. PGCs were subsequently pelletised by centrifugation, resuspended in phosphate-buffered saline (PBS) and then pelletised again. RNA was purified from the washed cell pellet using the Qiagen RNeasy Mini kit (Qiagen 74104) according to the manufacturer’s instructions. RNA concentration was measured using the NanoDrop® Spectrophotometer (Thermo Scientific ND-1000). RNA quality was assessed using the Agilent RNA 6000 Nano Kit (Agilent Technologies 5067-1511) and the 2100 Bioanalyzer (Agilent Technologies G2939BA). All the RNA samples used in RNA sequencing (RNA-seq) had an RNA integrity number (RIN) ranging from 8.0 to 10.0.

cDNA libraries were constructed by the Beijing Genomics Institute (BGI, Hong Kong, China). Briefly, poly-A containing mRNA molecules were purified using poly-T oligo-attached magnetic beads (New England Biolabs) that were subsequently fragmented into shorter mRNA fragments using divalent cations under an elevated temperature (Ambion® RNA fragmentation reagents kit, Thermo Fisher Scientific). Reverse transcriptase and a random primer (Invitrogen) were then used to synthesise First-strand cDNA from the cleaved RNA fragments. Second strand cDNA synthesis proceeded with a DNA Polymerase I (New England Biolabs) and RNase H (Invitrogen), and the RNA template was removed. An ‘A’ base was added to the resulting cDNA fragments, that was subsequently ligated to an adapter. Finally, the products were enriched by PCR before purification with the MinElute PCR Purification Kit (Qiagen). This purified cDNA library was used in RNA-Seq analysis.

RNA-seq reads were generated by BGI using the Illumina Novaseq 6000 system. Following sequencing, initial analysis was conducted by BGI using SOAPnuke software (developed by BGI) to filter the raw sequence reads using the following parameters: - n 0.01 -l 20 -q 0.4 -A 0.25 --cutAdaptor -Q 2 -G --polyX 50 --minLen 150 -i. An entire read was removed if 25% of its sequence matched the adaptor sequence. To filter low-quality data, an entire read was removed if more than 50% of its bases had a quality value lower than 20. Also, an entire read was deleted if the frequency of unknown bases was greater than 5%. The remaining filtered reads (over 78 million per sample), defined as ‘clean reads’ were stored in FASTQ format. FASTQ files were imported into the CLC Genomics Workbench v20.0.4 (Qiagen) for quality-control processing and analysis. RNA-seq data are deposited in the GEO and SRA archives at NCBI (Accession number GSE182397).

RNA-seq read mapping and DEG analysis was performed as previously described^41,42^. Briefly, RNA-seq reads were subjected to quality trimming before mapping to the ENSEMBL galGal5-annotated assembly (GRCg6a; 15-12-2020) for alignment and quantitative analysis of expression using the ‘RNA-Seq Analysis’ tool of the CLC Genomics Workbench (CLC Bio v2.18). For quantitative analysis, trimmed mean of M-values normalization (TMM) of the trimmed data set was performed by filtering out genes showing zero or NaN expression values. The fold change and False Discovery Rates (Bonferroni) were calculated using the RNA-Seq Analysis tool while differential expression within the RNA-Seq data was analysed using the Differential Expression for RNA-Seq tool of the CLC Genomic Workbench (CLC Bio v2.2). HEAT Maps were generated from TMM-normalised samples using the ‘Create HEAT Map for RNA-Seq’ tool in the same suite, using Euclidean Distance and Complete Cluster Linkage. Principal component analysis (PCA) plots were generated from the TMM-normalised samples using the ‘PCA for RNA-Seq’ tool in the same suite.

### Generation of GE chickens

Male and female GE PGCs were micro-injected separately into stage 15-16^+^ HH (ED 2.5) surrogate iCaspase9 host embryos as described previously^16^. Briefly, 1.0 µl of 25 mM B/B compound (in DMSO) (Takara Bio) was added to a 50 µl cell suspension (5000 PGCs/µl) and maintained at room temperature. ANP32A^N129I-D130N^: one male (FR5M#7) or three female (FR3F#3, FR3F#40 and FR6F#10) clonal GE PGC lines. 1.0 µl of the B/B compound-PGC mixture was injected into the dorsal aorta of embryos in windowed eggs. Egg shells were sealed with medical Leukosilk tape (BSN Medical) and then incubated until hatch. Surrogate males and female chickens (G0) were mated in pens to produce homozygous GE offspring. To screen for homozygous ANP32A^N129I-D130N^ embryos or chicks, chorioallantoic membrane (CAM) (for embryos) or CAM and blood (from hatchlings) were analysed by PCR amplification of genomic DNA and Sanger sequencing using primers (5′–ACTCCTTTTGTCACGAGAAGC–3′, 5′–TTCCTCCTCATCGTCTAAGCC–3′). To screen for homozygous AKO embryos, CAM were analysed by PCR amplification of genomic DNA and Sanger sequencing using primers (5′– TCAAAGTCCCTTATTACCGCG–3′, 5′–CCTTTCACTCCCCATCTTTCA–3′) that bind to areas outside the deleted 15-kb region and amplify a PCR product of approximately 220 bp only if the deletion is successful but fail to yield a product if there is no deletion. GE chickens received routine vaccinations and blood samples were sent to Sci-Tech Labs (Cawood Scientific), Dublin, Ireland to perform ELISA tests to assess response to vaccines.

### Western blot analysis

To analyse ANP32A expression in gene-edited clonal lines (Supplementary Fig. 3 and Supplementary Fig. 17k), at least 150,000 cells were lysed in 50 µl of 1X RIPA lysis buffer (sc-24948, Santa Cruz Biotechnology) containing protease and phosphatase inhibitor (Halt, Thermo Scientific 78440) according to the manufacturer’s instruction. To analyse ANP32A expression in AKO embryos (Supplementary Fig. 14g), approximately 2 mg of embryonic tissue was lysed in 100 µl of 1X RIPA lysis buffer (sc-24948, Santa Cruz Biotechnology) containing protease and phosphatase inhibitor (Halt, Thermo Scientific 78440) according to the manufacturers’ instruction. Denaturing electrophoresis and western blotting were performed using the NuPAGE electrophoresis system (Invitrogen) following the manufacturer’s protocol. Immunoblotting was performed using the following primary antibodies; rabbit anti-ANP32A (Sigma-Aldrich AV40203; 1/1000 dilution) and mouse anti-ɣ-tubulin (Sigma-Aldrich TS6557; 1/1000 dilution). The following secondary antibodies were used: goat anti-mouse IRDye 800CW (LI-COR 925-32211; 1/10,000 dilution) and goat anti-rabbit IRDye 680RD (LI-COR 925-68070; 1/10,000 dilution). Protein bands were visualised through fluorescence using the Odyssey Imaging System (LI-COR) according to the manufacturer’s instruction.

### PGC differentiation into adherent fibroblast-like cells

150,000 PGCs were incubated in 500 µl of high calcium FAOT medium containing 1.8 mM CaCl_2_ in fibronectin-coated or gelatin-coated wells in 24-well plates for 48 h. Subsequently, the FAOT medium was replaced with PGC fibroblast cell culture medium and then refreshed every 48 h by taking out 300 µl and adding back 300 µl of PGC fibroblast medium. Adherent fibroblast-like cells were visible with 48 h of culture in PGC fibroblast medium. Cell culture medium was refreshed every 48 h and cells were split 1:3 once they are reached 85-90% confluency in 24-well plates. PGC fibroblast-like cell culture medium contains 10% ES-grade foetal bovine serum (Life Technologies #16141061), 1% chicken serum (Biosera #CH-515), 0.1% 100x NEAA (Life Technologies #11140050), 0.1% sodium pyruvate (Life Technologies #11360070), 0.1% 100x GlutaMax (Life Technologies #35050-038), and 50 ng/ml ovotransferrin (Sigma-Aldrich C7786) in Knockout DMEM (Life Technologies #10829018). PGC fibroblast cultures were maintained at 37 °C in a 5% CO_2_ atmosphere.

### Cells and cell culture

Madin-Darby canine kidney (MDCK) cells (ATCC) were maintained in cell culture medium containing Dulbecco’s modified Eagle’s medium (Life Technologies) supplemented with 10% foetal bovine serum (FBS) (Life Technologies) and 1% penicillin-streptomycin (Life Technologies) at 37 °C in a 5% CO2 atmosphere. ANP32A-ANP32B-ANP32E-triple-knockout human eHAP1 cells (Horizon Discovery) lacking ANP32 expression were generated by Dr Ecco Staller at the University of Oxford^9^ and maintained in Iscove’s modified Dulbecco’s medium (Thermo Fisher) supplemented with 10% FBS, 1% nonessential amino acids (Life Technologies), and 1% penicillin/streptomycin.

### Minigenome replication assay

Influenza polymerase activity was assessed by using the chicken polI minigenome system as described previously^8,10,43^. pCAGGS expression plasmids encoding each polymerase component and NP for H5N1 50–92, H5N1 ty/05 and H9N2-UDL are described previously^8,43^. To measure influenza polymerase activity, PGC fibroblasts were transfected using Lipofectamine LTX (Invitrogen) according to manufacturers’ instructions in 24-well plates with pCAGGS plasmids encoding the PB1 (100 ng), PB2 (100 ng), PA (100 ng) and NP (100 ng) proteins, together with 100 ng chicken-specific minigenome reporter, either Empty pCAGGS or pCAGGS expressing chicken ANP32A (100 ng) and, as an internal control, 100 ng Renilla luciferase expression plasmid (pCAGGS-Renilla). Cells were incubated at 37 °C, and 48 h after transfection, cells were lysed with 120 ml of passive lysis buffer (Promega). Firefly and Renilla luciferase bioluminescence were measured using a Dual-luciferase system (Promega) with an EG&G Berthold LB 96 Microplate Luminometer or a Cytation 3 plate reader (Agilent-BioTek).

To assess polymerase activity in ANP32A-ANP32B-ANP32E-triple-knockout eHAP1 cells, cells were transfected using Lipofectamine 3000 (Invitrogen) in 24-wells with pCAGGS expression plasmids encoding H9N2-UDL PB1 (0.04 µg), PB2 (0.04 µg), PA (0.02 µg), NP (0.08 µg), chicken or human-specific Firefly minigenome reporter (0.08 µg) and *Renilla* luciferase expression plasmid (0.04 µg) together with chicken ANP32-FLAG or human ANP32-FLAG or empty expression plasmid (0.04 µg) and incubated at 37 °C for 24 h. Cells were subsequently lysed in 50 µl passive lysis buffer (Promega) for 30 min with gentle shaking at room temperature. Firefly and *Renilla* bioluminescence were measured using the dual-luciferase system (Promega) with a FLUOstar Omega plate reader (BMG Labtech).

### In vivo challenge of chickens with influenza virus and transmission to naïve sentinels

Mixed sex commercial Hy-line layer chickens (WT and ANP32A^N129I-D130N^) were hatched at the National Avian Research Facility, Midlothian, United Kingdom and transported at 1 day of age to The Pirbright Institute, Surrey, UK. Pre-infection sera were obtained from all chickens, and all were negative for reactive influenza antibodies against A/chicken/Pakistan/UDL01/08 (H9N2-UDL) by haemagglutinin inhibition assay (HI). Chickens were housed in two groups according to genotype, WT or ANP32A^N129I-D130N^. At 7 days of age, chickens to be directly inoculated were housed in negative pressured BioFlex® B50 Rigid Body Poultry isolators (Bell Isolation Systems). At 2 weeks of age, chickens in isolators were inoculated intranasally with 100 μl (50 μl per nare) of H9N2-UDL virus, either at a low dose of virus (1 × 10^3^ PFU) or high dose (1 × 10^6^ PFU). 24 h after direct inoculation, naïve sentinel birds were introduced into the isolators to assess transmission. Numbers of birds involved in each experiment are detailed in Figs. 2 and 3. 14 days post-inoculation, birds were humanely euthanised by intravenous administration of sodium pentobarbital, blood was collected by cardiac puncture and sera collected for analysis of virus-specific antibodies by HI. Infection of birds was assessed by swabbing both the oropharyngeal and cloacal cavities daily with sterile polyester tipped swabs (Fisher Scientific, UK) which were transferred into viral transport media, vortexed briefly, clarified and stored at −80 °C prior to virus detection^44^.

### Plaque assay

Infectious virus titration was by plaque assay on MDCK cells. MDCKs were inoculated with 10-fold serially diluted samples and overlaid with 2% agarose (Oxoid) in supplemented DMEM (1× MEM, 0.21% BSA V, 1 mM L-Glutamate, 0.15% Sodium Bicarbonate, 10 mM Hepes, 1× Penicillin/Streptomycin (all Gibco) and 0.01% Dextran DEAE (Sigma-Aldrich, Inc.), with 2 µg/ml TPCK trypsin (SIGMA). MDCKs were then incubated at 37 °C for 72 h. Plaques were developed using crystal violet stain containing methanol. The limit of virus detection in the plaque assays was 5 pfu/ml.

### Haemagglutination inhibition assay

Haemagglutinin inhibition (HI) assays were carried out using the challenge virus H9N2-UDL. HI assays were performed according to standard procedures^45^. Samples with titres below 2 HI units were considered negative.

### Sequencing of viruses isolated from chicken oropharyngeal swabs

To sequence viruses recovered from directly inoculated and sentinel chickens, 100 µl of clarified oropharyngeal swab sample was inoculated into embryonic day (ED) 10 chicken eggs (Valo BioMedia GmbH) and incubated at 37 °C with turning for 72 h before allantoic fluid was harvested. Viral RNA was extracted from allantoic fluid using RNA extracted from each concentration, using the QIAmp viral RNA minikit (Qiagen) according to manufacturer instructions and virus cDNA produced using SuperScript IV (Invitogen) RT-PCR using the universal primer Optil-R1: GTT ACG CGC CAG TAG AAA CAA GG according to the manufacturer’s instructions. PCR amplification of all eight influenza A virus segments was achieved by PCR with a mixture of three universal primers (Optil-F1: GTT ACG CGC CAG CAA AAG CAG G, Optil-F2: GTT ACG CGC CAG CGA AAG CAG G and Optil-R1: GTT ACG CGC CAG TAG AAA CAA GG) and Q5® High-fidelity DNA polymerase (NEB). 1 ng of dsDNA was used to prepare sequencing libraries using the Nextera XT DNA kit (Illumina). Pooled libraries were sequenced on a 2x300cycle MiSeq Reagent Kit v2 (Illumina, USA) and analysed using Geneious Prime 2019 software (Biomatters Inc.).

### Human airway epithelial cell infection

Triplicate wells of primary human airway epithelial (HAE) cells (purchased from Epithelix Sarl, Inc) at an air–liquid interface were washed apically with 200 µl serum-free DMEM (Gibco) to remove mucus before being infected with 0.01 PFU/cell of virus for 1 hr. Inoculum was removed and the apical surface washed twice. At timepoints post-infection 200 µl serum-free DMEM was added to the apical surface and removed after 10 mins at 37 °C to take harvests for plaque assay or variant quantification for competition assay.

### Competition assay between UDL WT and mutant viruses

Chicken eggs (*n* = 4) or human airway epithelial cell inserts (*n* = 3) were co-infected with a mixture of viruses and harvests taken at timepoints post-infection. Viral RNA was extracted using MagMAX™ Viral/Pathogen kit on KingFisher™ Flex Purification System (Thermo), cDNA generated using Superscript III (Thermo) and Uni-12 primer. A pair of ~200 bp amplicons across position 349 of the PA gene and position 631 of the PB2 gene were generated by PCR for each sample and were designed to include one of four 4 bp terminal barcodes (CACA, GTTG, AGGA or TCTC) using the primers in Supplementary Table 5. The pair of PA and PB2 amplicons for each sample were combined. Pools of samples were made using samples representing each of the four unique barcodes and a second barcode was added to the pooled amplicons using NEBNext® Ultra™ II DNA Library Prep Kit for Illumina (NEB). Pooled samples were pooled together and sequenced with Illumina MiSeq v2 150 PE micro kit (Illumina). Sequencing reads were demultiplexed and variant proportions at each locus quantified.

### Statistical analysis and graphical illustration

Statistical analysis of biological replicates was performed by unpaired two-tailed T-test, One-way ANOVA with Dunnett’s multiple comparison test, or Mann–Whitney test using GraphPad Prism 9. Sample sizes were not predetermined using any statistical methods. Some illustrations in Figs. 1 and 5 were generated using BioRender.com.

### Reporting summary

Further information on research design is available in the Nature Portfolio Reporting Summary linked to this article.

### Supplementary information

Supplementary Information File

Reporting Summary

Peer Review File

### Source data

Source Data

## Data Availability

The data supporting the findings of this study are available within the article and its Supplementary Information. The source data for the main figures and extended data figures are provided as Source Data files. Illumina RNA sequencing data for PGC transcriptome analysis are deposited in the GEO and SRA archives at NCBI (Accession number GSE182397). Viral sequencing data were deposited at http://www.ebi.ac.uk/ena (project number PRJEB65964). The authors declare that all unique materials used are readily available from the authors upon MTA agreement. Source data are provided with this paper.

## References

[1] Long, J. S., Mistry, B., Haslam, S. M. & Barclay, W. S. Host and viral determinants of influenza A virus species specificity. *Nat. Rev. Microbiol.* 1 (2018). 10.1038/s41579-018-0115-z 30487536

[2] Fasanmi OG (2018). National surveillance and control costs for highly pathogenic avian influenza H5N1 in poultry: A benefit-cost assessment for a developing economy, Nigeria. Res. Vet. Sci..

[3] Thompson JM, Seitzinger AH (2019). Economic evaluation of low pathogenic avian influenza in northeastern US live bird markets. J. Appl. Poult. Res..

[4] Wang D, Zhu W, Yang L, Shu Y (2021). The epidemiology, virology, and pathogenicity of human infections with avian influenza viruses. Cold Spring Harb. Perspect. Med..

[5] Wille, M. & Barr, I. G. Resurgence of avian influenza virus. *Science* **376**, 459–460 (2022). 10.1126/science.abo1232 35471045

[6] Peters AR, Guyonnet V (2020). Are current avian influenza vaccines a solution for smallholder poultry farmers?. Gates Open Res..

[7] Wandzik JM, Kouba T, Cusack S (2020). Structure and function of influenza polymerase. Cold Spring Harb. Perspect. Med..

[8] Long JS (2016). Species difference in ANP32A underlies influenza A virus polymerase host restriction. Nature.

[9] Staller, E. et al. ANP32 proteins are essential for influenza virus replication in human cells. *J. Virol.* **93**, e00217-19 (2019). 10.1128/JVI.00217-19 PMC6694824 31217244

[10] Long, J. S. et al. Species specific differences in use of ANP32 proteins by influenza A virus. *Elife* **8**, e45066 (2019). 10.7554/eLife.45066 PMC6548507 31159925

[11] Park YH (2020). Host-specific restriction of avian influenza virus caused by differential dynamics of ANP32 family members. J. Infect. Dis..

[12] Carrique L (2020). Host ANP32A mediates the assembly of the influenza virus replicase. Nature.

[13] Zhang H (2019). Fundamental contribution and host range determination of ANP32A and ANP32B in influenza A virus polymerase activity. J. Virol..

[14] Ran FA (2013). Genome engineering using the CRISPR-Cas9 system. Nat. Protoc..

[15] Idoko-Akoh A, Taylor L, Sang HM, McGrew MJ (2018). High fidelity CRISPR/Cas9 increases precise monoallelic and biallelic editing events in primordial germ cells. Sci. Rep..

[16] Ballantyne M (2021). Direct allele introgression into pure chicken breeds using Sire Dam Surrogate (SDS) mating. Nat. Commun..

[17] Cornelis FMF (2018). ANP32A regulates ATM expression and prevents oxidative stress in cartilage, brain, and bone. Sci. Transl. Med..

[18] Monteagudo S (2022). ANP32A represses Wnt signaling across tissues thereby protecting against osteoarthritis and heart disease. Osteoarthritis Cartilage.

[19] Peacock TP, James J, Sealy JE, Iqbal M (2019). A global perspective on H9N2 avian influenza virus. Viruses.

[20] Zhao Y (2016). Adaptive amino acid substitutions enhance the virulence of a novel human H7N9 influenza virus in mice. Vet. Microbiol..

[21] Zhang X (2017). Enhanced pathogenicity and neurotropism of mouse-adapted H10N7 influenza virus are mediated by novel PB2 and NA mutations. J. Gen. Virol..

[22] Choi WS (2017). Rapid acquisition of polymorphic virulence markers during adaptation of highly pathogenic avian influenza H5N8 virus in the mouse. Sci. Rep..

[23] Chen KY, Afonso EDS, Enouf V, Isel C, Naffakh N (2019). Influenza virus polymerase subunits co-evolve to ensure proper levels of dimerization of the heterotrimer. PLoS Pathog..

[24] Brown EG, Liu H, Chang Kit L, Baird S, Nesrallah M (2001). Pattern of mutation in the genome of influenza A virus on adaptation to increased virulence in the mouse lung: Identification of functional themes. Proc. Natl Acad. Sci. USA.

[25] Hatcher EL (2017). Virus Variation Resource – improved response to emergent viral outbreaks. Nucleic Acids Res..

[26] Park YH (2021). Asp149 and Asp152 in chicken and human ANP32A play an essential role in the interaction with influenza viral polymerase. FASEB J..

[27] Camacho-Zarco AR (2020). Molecular basis of host-adaptation interactions between influenza virus polymerase PB2 subunit and ANP32A. Nat. Commun..

[28] Sugiyama K, Kawaguchi A, Okuwaki M, Nagata K (2015). PP32 and APRIL are host cell-derived regulators of influenza virus RNA synthesis from cRNA. Elife.

[29] Fan H (2019). Structures of influenza A virus RNA polymerase offer insight into viral genome replication. Nature.

[30] Chang S (2015). Cryo-EM structure of influenza virus RNA polymerase complex at 4.3 Å resolution. Mol. Cell.

[31] Chen G, Liu CH, Zhou L, Krug RM (2014). Cellular DDX21 RNA helicase inhibits influenza A virus replication but is counteracted by the viral NS1 protein. Cell Host Microbe.

[32] Sun, L. et al. The SUMO-interacting Motif in NS2 promotes adaptation of avian influenza virus to mammals. *bioRxiv*10.1101/2022.12.11.519849 (2022). 10.1126/sciadv.adg5175 PMC10337910 37436988

[33] Mänz B, Brunotte L, Reuther P, Schwemmle M (2012). Adaptive mutations in NEP compensate for defective H5N1 RNA replication in cultured human cells. Nat. Commun..

[34] Tait-Burkard C (2018). Livestock 2.0 - Genome editing for fitter, healthier, and more productive farmed animals. Genome Biol..

[35] Knap PW, Doeschl-Wilson A (2020). Why breed disease-resilient livestock, and how?. Genet. Sel. Evol..

[36] Lyall J (2011). Suppression of avian influenza transmission in genetically modified chickens. Science.

[37] Schusser, B. & Doran, T. Advances in genetic engineering of the avian genome. *Avian Immunol.*10.1016/B978-0-12-818708-1.00022-1 (2022).

[38] Whyte J (2015). FGF, insulin, and SMAD signaling cooperate for avian primordial germ cell self-renewal. Stem Cell Rep..

[39] Gu B (2020). Opposing effects of cohesin and transcription on CTCF organization revealed by super-resolution imaging. Mol. Cell.

[40] Labun K, Montague TG, Gagnon JA, Thyme SB, Valen E (2016). CHOPCHOP v2: a web tool for the next generation of CRISPR genome engineering. Nucleic Acids Res..

[41] Giotis ES (2016). Chicken interferome: avian interferon-stimulated genes identified by microarray and RNA-seq of primary chick embryo fibroblasts treated with a chicken type I interferon (IFN-α). Vet. Res..

[42] Giotis ES, Montillet G, Pain B, Skinner MA (2019). Chicken Embryonic-stem Cells Are Permissive To Poxvirus Recombinant Vaccine Vectors. Genes.

[43] Long JS (2013). The effect of the PB2 mutation 627K on highly pathogenic H5N1 avian influenza virus is dependent on the virus lineage. J. Virol..

[44] World Health Organisation. Collecting, preserving and shipping specimens for the diagnosis of avian influenza A(H5N1) virus infection: guide for field operations. ‘October 2006’.

[45] Pedersen JC (2008). Hemagglutination-inhibition test for avian influenza virus subtype identification and the detection and quantitation of serum antibodies to the avian influenza virus. Methods Mol. Biol..

