# Supplementary material for: Creating resistance to avian influenza infection through genome editing of the ANP32 gene family
Source: Nat Commun. 2023 Oct 10;14:6136. doi: 10.1038/s41467-023-41476-3 (PMC10564915; doi:10.1038/s41467-023-41476-3)
Supplement: Supplementary file 1 — Supplementary Information File [file 41467_2023_41476_MOESM1_ESM.pdf]

SUPPLEMENTARY INFORMATION

Creating resistance to avian influenza infection through genome editing of the ANP32 gene family.

Alewo Idoko-Akoh, Daniel H. Goldhill, Carol M. Sheppard, Dagmara Bialy, Jessica L. Quantrill, Ksenia Sukhova, Jonathan C. Brown, Samuel Richardson, Ciara Campbell, Lorna Taylor, Adrian Sherman, Salik Nazki, Jason S. Long, Michael A. Skinner, Holly Shelton, Helen M. Sang, Wendy S. Barclay, Mike J. McGrew

Supplementary Table 1. Injection and hatching of iCaspase9 surrogate hosts.

Injection set	Sex of edited PGCs	No. of donor PGCs injected per host embryo	Surrogate host genotype	ED 14 survival rate (% injected embryos)	No. & sex of hatchlings (% hatch rate)
ANP-1	M	4,000	iCaspase9	17/19 (89%)	10: 5F + 5M (59%)
ANP-2	F	4,000	iCaspase9	22/23 (96%)	12: 6F + 6M (52%)
ANP-3	F	4,000	iCaspase9	12/14 (86%)	6: 1F + 5M (50%)
ANP-4	F	3,000	iCaspase9	16/22 (73%)	7: 5F + 2M (44%)

Supplementary Table 2. Hatching of G_1 homozygous ANP32A$^{N129I\text{-}D130N}$ edited chicks for challenge experiments using surrogate sire/surrogate dam mating.

Surrogate host mating groups	No. of eggs laid per hen per week	Eggs set	Fertility$ (% eggs set)	Hatchability (% fertile)
ANP1-12_1104♂ × ANP2-2_1110♀ ANP2-13_1117♀ ANP2-22_1122♀ ANP3-6_1127♀	3.1	24	19 (79)%	12 (63%)
ANP4-7_1158♂ × ANP4-9_1159♀ ANP4-14_1162♀ ANP4-15_1163♀ ANP4-21_1164♀	6.2	75	62 (83)%	51 (82%)
Total		99	81 (82)%	63 (78%)

Data is shown for two independent hatching egg sets from each pen.
*Lay rate; eggs were counted over a 60-day period when hens were between 7-11 months of age and divided by the number of fertile hens present in pen. The maximum possible lay rate is 7.0 eggs per hen per week.
$Fertility was assessed at incubation day 14.

Supplementary Table 3. Sequences of gRNA and ssODN repair templates.

	Sequence 5' → 3 '
Female PGC ssODN donor sequence	AAAAAGTTAGAAAACCTGAAGAGTTTAGATCTTTTCAATTGCGAGGTAACaAACTTGAtTaATTATAGAGAAAACGTATTCAAGCTCCTCCCACAACTCACATACCTCGATGGCTA
Male PGC ssODN donor sequence	AAAAAGTTAGAAAACCTGAAGAGTTTAGATCTTTTCAATTGCGAGGTAACgAACTTGAtTaATTATAGAGAAAACGTATTCAAGCTCCTCCCACAACTCACATACCTCGATGGCTA
ANP32A exon 4 gRNA sequence	TTCTCTATAATCATTCAAGT
ANP32A exon 1 gRNA1 sequence	CGGCCATGGACATGAAGAAA
ANP32A exon 1 gRNA2 sequence	TCCACTTAGAGCTGCGGAAC
ANP32A intron 5 gRNA sequence	CATTCCCCTCGCTCCTTCAA
ANP32B gRNA1 (promoter region)	TTCCTCTATATAAGCGGCGG
ANP32B gRNA2 (exon 1)	GATGAAAAAGCGGCTCACGC
ANP32E gRNA1 (exon 2)	GCCACCCTCCCTCGCCAGTG
ANP32E gRNA2 (intron 2)	CAGATTCGGCGGAGCCGCCG

Supplementary Table 4. Sequences of PCR primers for off-target sites for ANP32A exon 4 gRNA.

Off-target sites	Sequence 5′ → 3 ′
Chromosome 1: 91872693	LEFT PRIMER: CCACAATGCAGCTCAGAGAAA<br>RIGHT PRIMER: GACTGAAAACCTATGTCAAAGCT
Chromosome 1: 153558607	LEFT PRIMER: CAGTTAAAAGCTTGAGACTGCA<br>RIGHT PRIMER: ACACCTGTATCCTCCTTCACC
Chromosome 4: 25712226	LEFT PRIMER: GCAGGCATAGATACCCATTCC<br>RIGHT PRIMER: AAGCCTTTTACACAGCAGGTC
Chromosome 6: 4920697	LEFT PRIMER: CGGAATCAATTTGGGGATGCT<br>RIGHT PRIMER: TTTGGGAGCTACAGTTGACCT

Supplementary Table 5. Primers used for generating PA and PB2 amplicons for sequencing of competition assay harvests.

Primer	Sequence	Primer use
PA_E349K_961_F	**XXXX**GGCTGGAAAGATCCCAACATTG	Barcoded forward primer for PA amplicon
PA_E349K_1131_R	CTTCCTCACCTAGTGCCCACTTCAAC	Reverse primer for PA amplicon
PB2_M631L_1805_F	**XXXX**AGATGCGTGATGTATTGGGAACA	Barcoded forward primer for PA amplicon
PB2_M631L_2001_R	CTTCAACTGTAAGCCTTTTGGTTGCC	Reverse primer for PB2 amplicon

XXXX represents **CACA, GTTG, AGGA or TCTC** barcodes.

Supplementary Figures

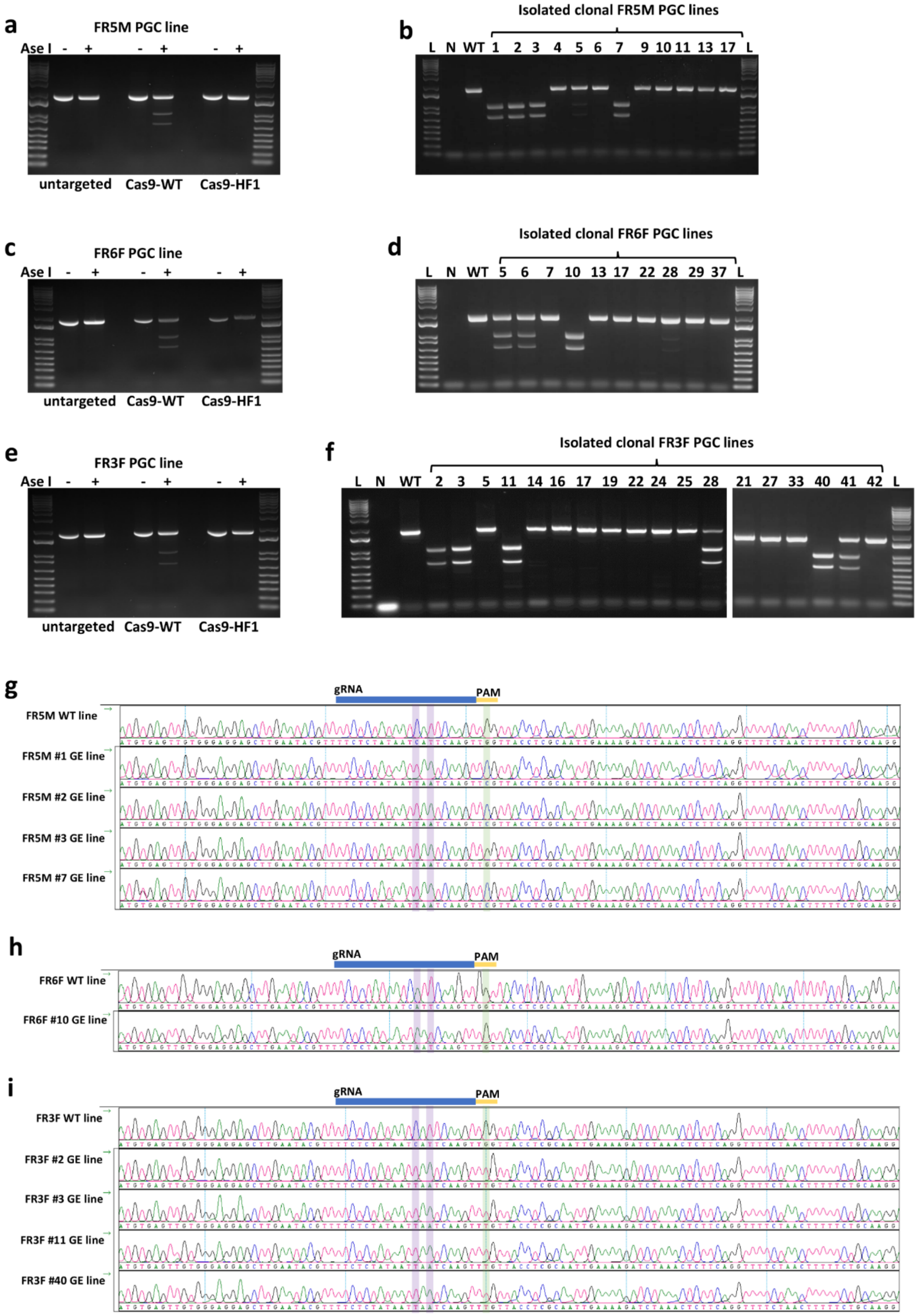

Supplementary Fig. 1. Genome editing of chicken PGCs.

Three independent PGC lines (FR5M male line **(a,b)**, FR6F female line **(c,d),** and FR3F female line **(e,f)**) were co-transfected with a ssODN repair template and a PX459 plasmid expressing gRNA, puromycin resistance gene and either wild-type Cas9 or high-fidelity Cas9-HF1. Transfected PGCs were puromycin-selected and assessed through *Ase* I restriction digest of PCR products to identify editing events. PGC pools targeted with Cas9-HF1 did not show any cleaved PCR products. PGC pools targeted with wild-type Cas9 showed cleaved PCR products indicating the presence of edited cells.

b,d,f, Single cell cultures from targeted PGC pools were assessed by *Ase* I restriction digest to identify edited clones. Complete cleavage of the 2-kb PCR product indicates a biallelic edit whereas partial cleavage indicates a monoallelic edit.

g,h,i, Sanger sequencing of PCR products from clonal PGC lines confirmed that they contained the expected biallelic sequence changes (highlighted by purple and green pane). *For **a, c,** and **e**, (+) refers to Ase I treated PCR product while (–) refers to untreated PCR product.* '*GE' refers to the ANP32A*$^{N129I\text{-}D130N}$ *genotype. 'WT' refers to wildtype genotype*

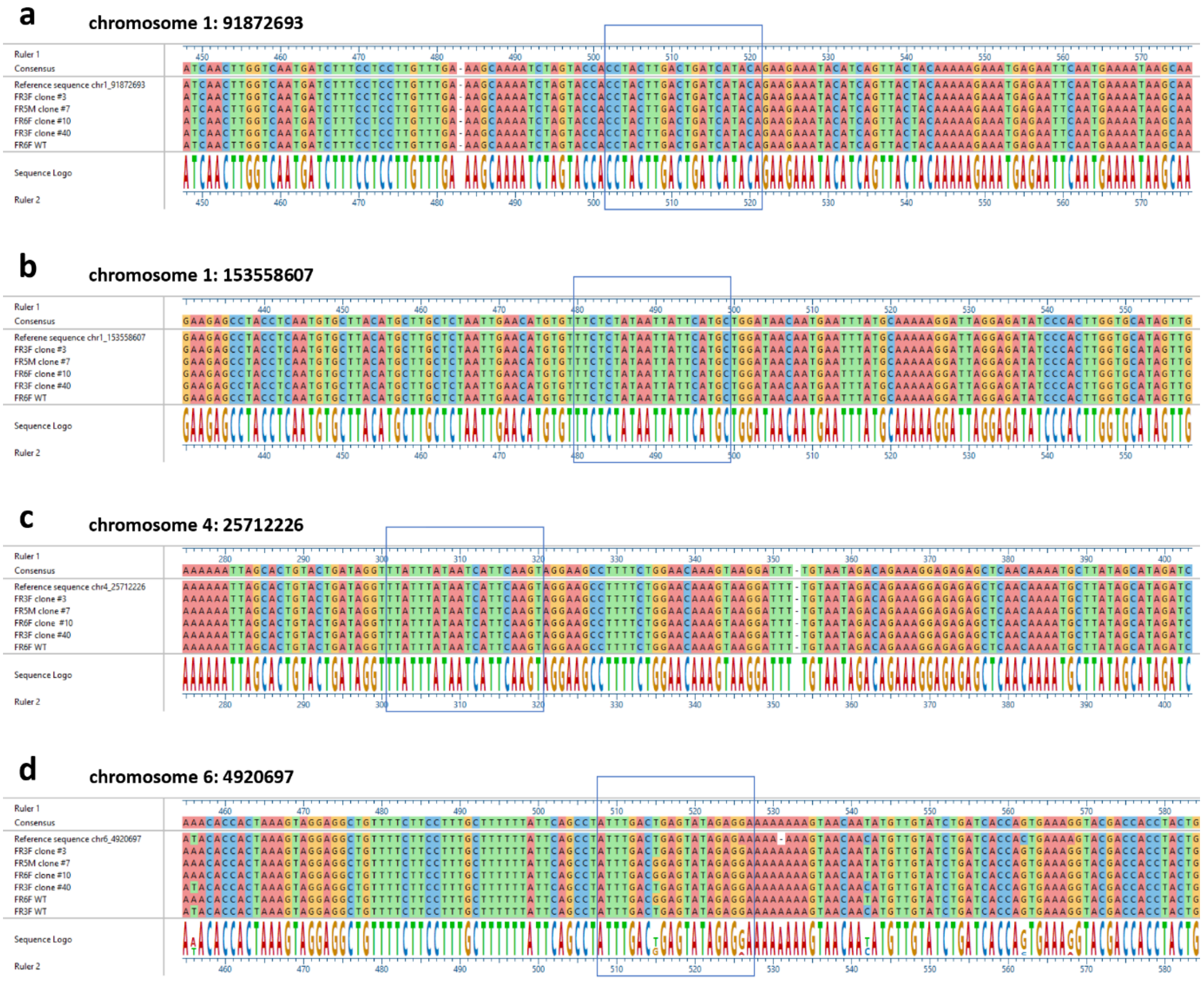

Supplementary Fig. 2. Analysis of predicted off-target sites in ANP32A$^{N129I-D130N}$ and wild-type (WT) PGCs.

FR3F clone #3, FR5M clone #7, FR6F clone #10 and FR3F clone #40 are clonal ANP32A$^{N129I-D130N}$ PGC lines. Potential off-target sites for the gRNA targeting exon 4 were predicted by the CHOPCHOP gRNA design web tool (http://chopchop.cbu.uib.no/). PCR amplification of the off-target sites in ANP32A$^{N129I-D130N}$ and WT PGCs was performed, followed by Sanger sequencing. Off-target sites similar to the gRNA sequence (5’-TTCTCTATAATCATTCAAGT-3’) are highlighted by the blue boxes. See **Supplementary Table 4** for PCR primers used for off-target analysis.

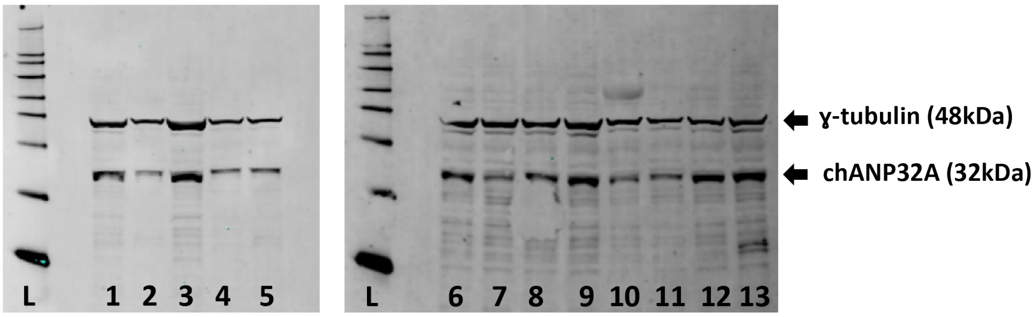

L - Molecular weight marker
1 - FR3F WT line
2 - FR3F #2 GE clonal line
3 - FR3F #3 GE clonal line
4 - FR3F #11 GE clonal line
5 - FR3F #40 GE clonal line
6 - FR5M WT line
7 - FR5M #1 GE clonal line
8 - FR5M #2 GE clonal line
9 - FR5M #3 GE clonal line
10 - FR5M #7 GE clonal line
11 - FR5M WT clonal line
12 - FR6F WT line
13 - FR6F #10 GE clonal line

Supplementary Fig. 3. ANP32A expression levels in wildtype (WT) and genome-edited (GE) cultured chicken PGC lines.

Western blot analysis was performed to assess ANP32A expression in clonal PGC lines confirmed as biallelically edited. ɣ-tubulin expression was used as a loading control. *GE indicates the ANP32A$^{N129I\text{-}D130N}$ homozygous genotype*.

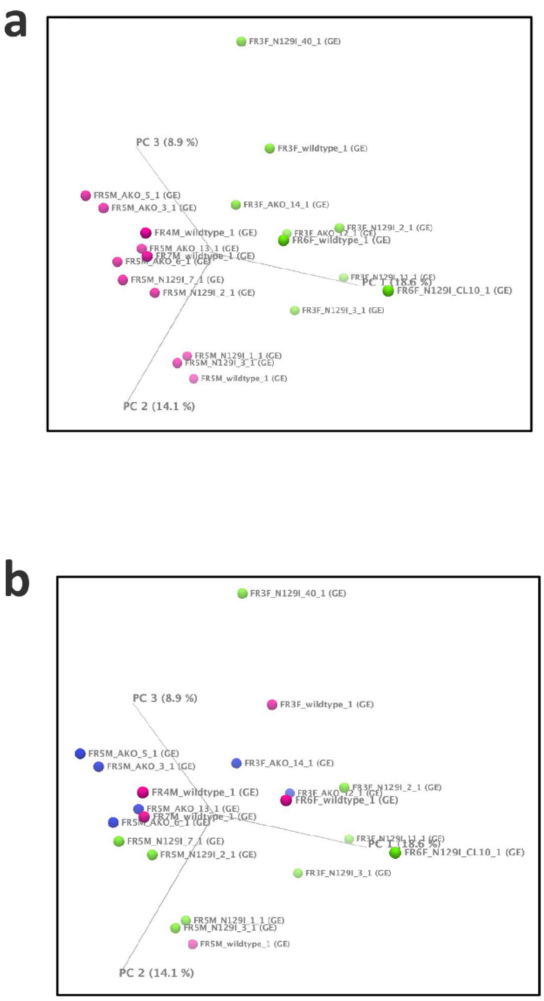

Supplementary Fig. 4. Three-dimensional principal component analysis (PCA) maps comparing wildtype and genome-edited (GE) cultured chicken (PGCs).

Comparison of total RNA expression profile is shown by: **a,** sample names with sex (male: red, green: female). **b,** sample names with genotype (wildtype: red, green: mutant; blue: knockout). *'Mutant' or 'N129I' refers to the ANP32A$^{N129I\text{-}D130N}$genotype. 'Knockout' or 'AKO' refers to the ANP32A^{knockout} genotype*.

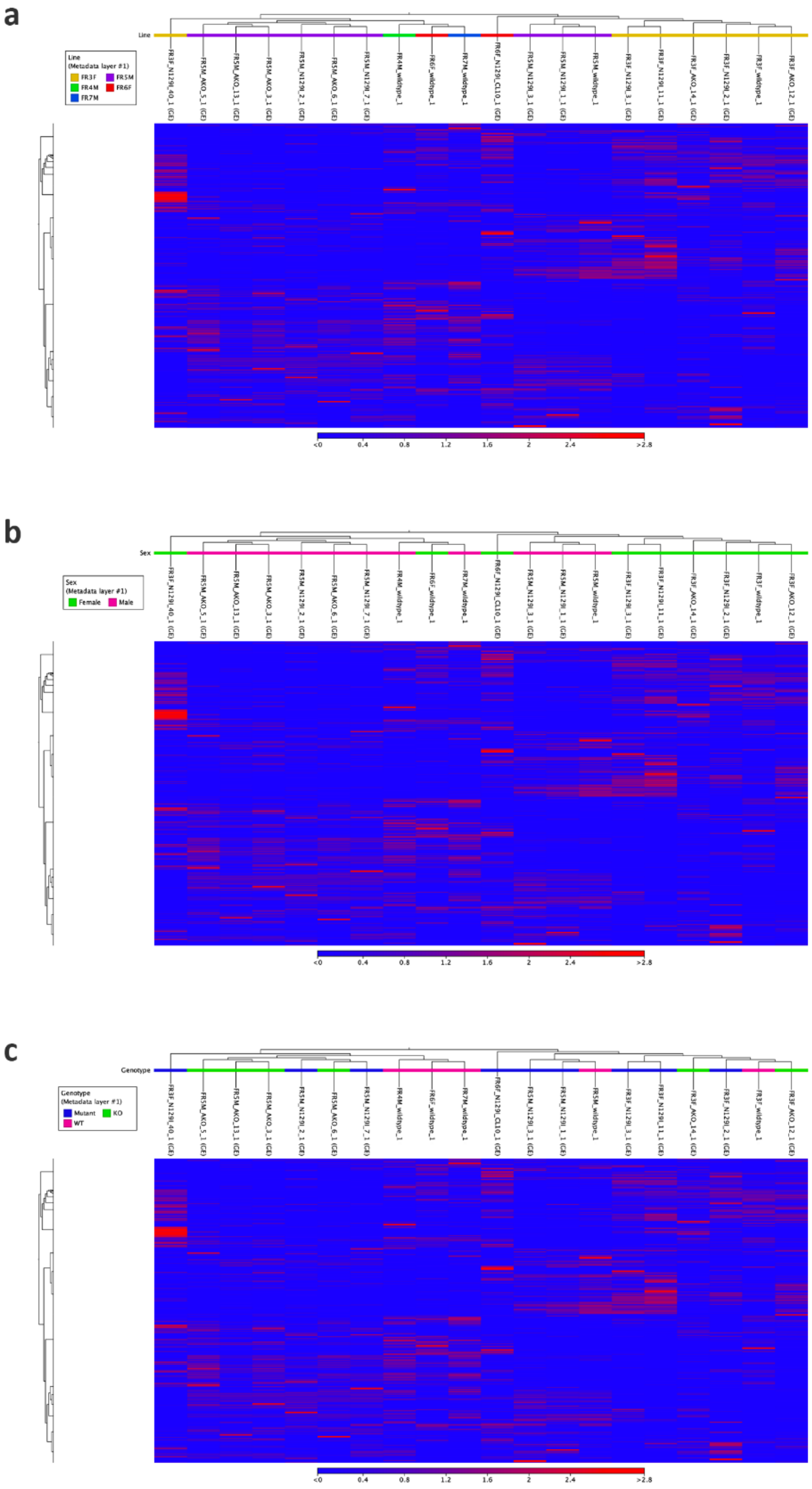

Supplementary Fig. 5. Heatmaps showing unhierarchical clustering of total RNA expression pattern in wildtype and genome-edited (GE) cultured chicken (PGCs).

Keys above the map show the nature of each of the named samples by line **(a)**, sex **(b)** and genotype **(c)**. The Log-CPM (logarithm of counts per million reads mapped) expression values

are visualized using the gradient colour scheme key below each map. *'Mutant' or 'N129I' refers to the ANP32A$^{N129I\text{-}D130N}$ genotype. 'KO' or 'AKO' refers to the ANP32A^{knockout} genotype.*

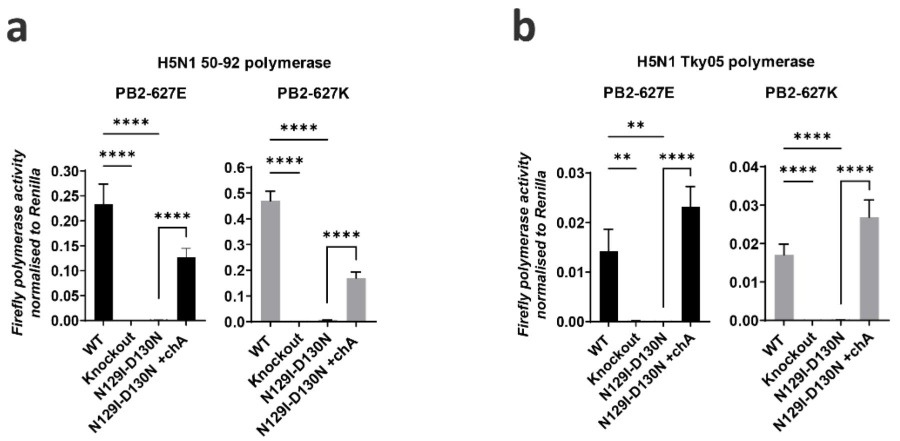

Supplementary Fig. 6. Activity of reconstituted IAV polymerase is restricted in ANP32A$^{N129I\text{-}D130N}$ edited chicken cells.

a-b, PGC-derived fibroblasts were transfected with empty vector (mock) or plasmids encoding avian IAV polymerase (PB2/627E - black bars) or human-adapted isoforms (PB2/627K - grey bars), Firefly minigenome reporter and Renilla reporter control plasmids and then incubated at 37°C for 48 hours. Wild-type chicken ANP32A (chA) was co-expressed with minigenome plasmids to rescue polymerase activity in ANP32A$^{N129I\text{-}D130N}$ cells. Data shown are Firefly activity normalised to Renilla, plotted as mean ± SEM; one-way ANOVA, with multiple comparisons to WT using Dunnett's test. Unpaired t-test was used to compare ANP32A$^{N129I\text{-}D130N}$ and ANP32A$^{N129I\text{-}D130N}$ +chA data. n=3 biological replicates. *WT* refers to wildtype cells. *Knockout* refers to *ANP32A^{knockout}* cells. *N129I-D130N* refers to ANP32A$^{N129I\text{-}D130N}$ cells. **=P≤0.01; ****=P≤0.0001. Error bars = ± standard error of the mean

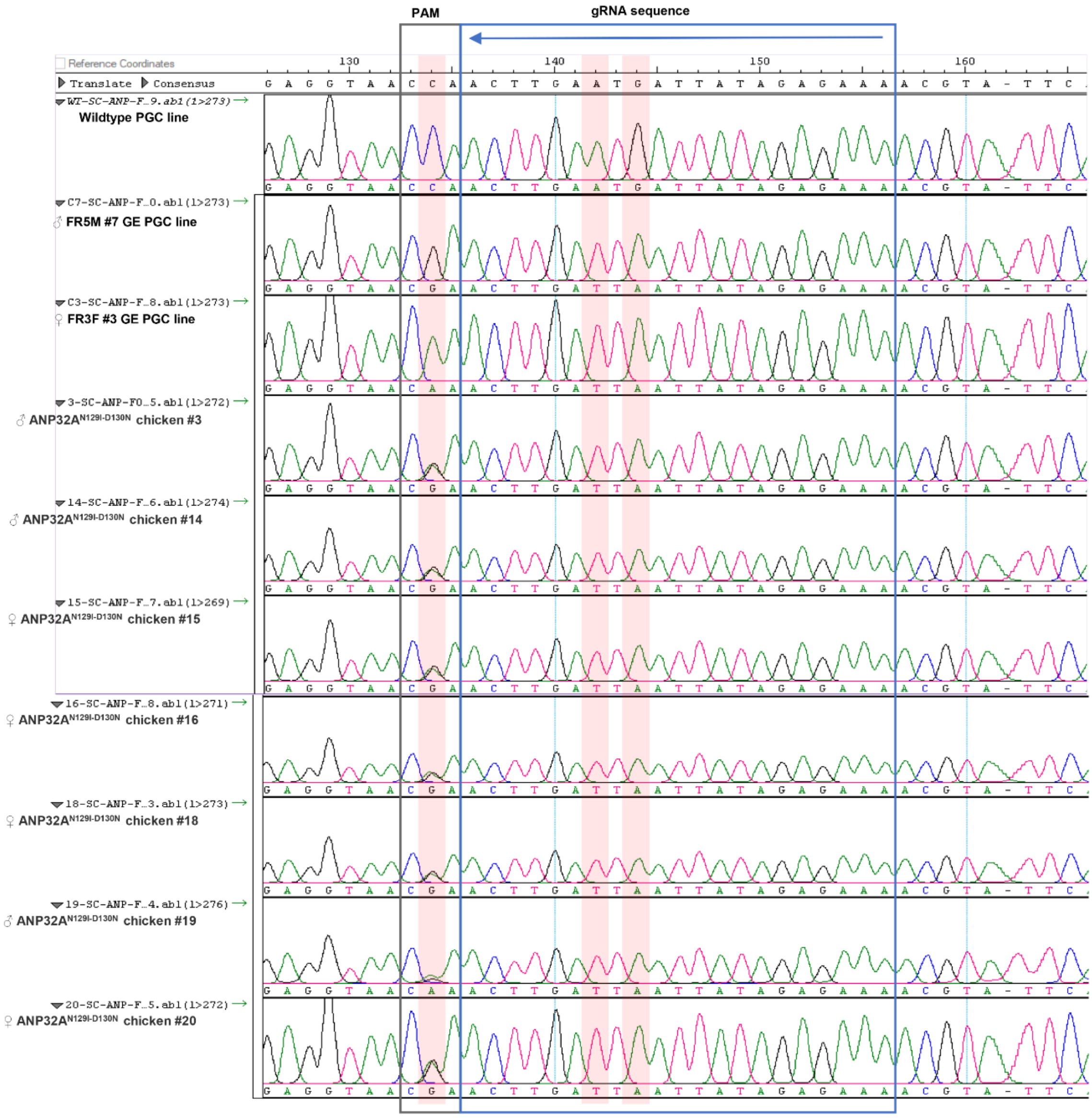

Supplementary Fig. 7. Representative Sanger sequencing chromatogram confirming introduction of sequence changes in GE (ANP32A$^{N129I-D130N}$) chickens (red panes).

The double peak in the PAM site confirms that the ANP32A$^{N129I-D130N}$ offspring is derived from one male and one female GE PGC.

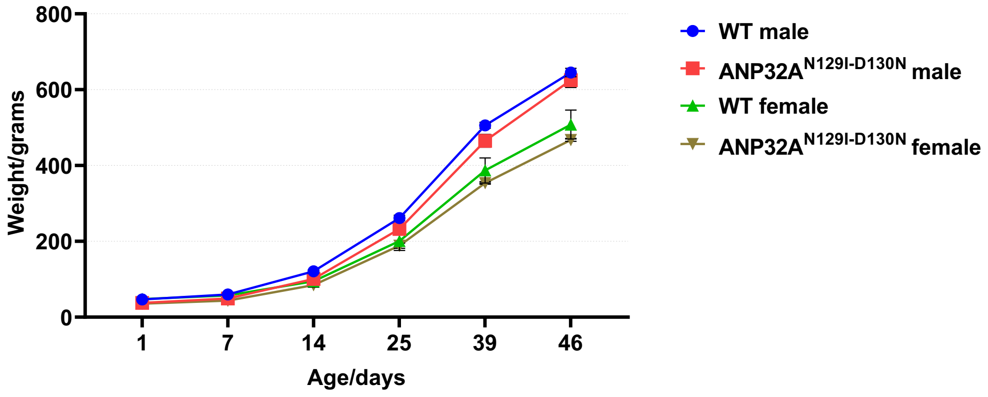

Age (days)	WT male vs ANP32A$^{N129I-D130N}$ male T-test P values	WT Female vs ANP32A$^{N129I-D130N}$ female T-test P values
1	0.002297 (**=P≤0.01)	0.019384 (*=P≤0.05)
7	0.094510 (ns)	0.087006 (ns)
14	0.037308 (*=P≤0.05)	0.450812 (ns)
25	0.064185 (ns)	0.530822 (ns)
39	0.047303 (*=P≤0.05)	0.187087 (ns)
46	0.388526 (ns)	0.166082 (ns)

Supplementary Fig. 8. Comparison of mean bodyweight (BW) between WT and ANP32A$^{N129I-D130N}$ chickens.

WT chicks: n= 2 females, 5 males; ANP32A$^{N129I-D130N}$ chicks: n= 2 males, 4 females. Error bars are ± standard error of the mean. Table shows p-values from unpaired T-test comparisons of mean bodyweights at various ages.

	Sample Label:	AE		CAV		EDS		IBD		IBV		ILT		NDV	
Sample Number		Result	Titre Gp	Result	Titre Gp	Result	Titre Gp	Result	Titre Gp	Result	Titre Gp	Result	Titre Gp	Result	Titre Gp
1	0865-ANP	[pos]	10	[pos]	3	[pos]	7	[pos]	7	[pos]	8	[pos]	1	[pos]	10
2	0866-ANP	[pos]	10	[pos]	3	[pos]	7	[pos]	5	[pos]	7	[neg]	0	[pos]	10
3	0867-ANP	[pos]	8	[neg]	0	[pos]	7	[pos]	7	[pos]	10	[neg]	0	[pos]	10
4	0868-ANP	[neg]	0	[pos]	2	[pos]	6	[pos]	8	[pos]	7	[neg]	0	[pos]	10
5	0869-ANP	[pos]	2	[neg]	0	[pos]	6	[pos]	4	[pos]	7	[pos]	2	[pos]	10
6	0870-ANP	[neg]	0	[neg]	0	[neg]	0	[pos]	6	[pos]	2	[pos]	3	[pos]	6
7	0871-ANP	[pos]	4	[neg]	0	[pos]	5	[pos]	8	[pos]	6	[pos]	3	[pos]	10
8	0872-WT	[pos]	7	[pos]	3	[pos]	7	[pos]	9	[pos]	8	[pos]	1	[pos]	10
9	0874-WT	[pos]	5	[pos]	2	[pos]	7	[pos]	8	[pos]	6	[neg]	0	[pos]	10
10	0875-WT	[neg]	0	[neg]	0	[pos]	7	[pos]	4	[pos]	6	[pos]	2	[pos]	10
11	0878-WT	[pos]	10	[pos]	8	[pos]	7	[pos]	8	[pos]	13	[pos]	5	[pos]	11
12	0880-WT	[pos]	10	[pos]	2	[pos]	7	[pos]	7	[pos]	9	[neg]	0	[pos]	11
13	0882-WT	[pos]	10	[pos]	2	[pos]	4	[pos]	6	[pos]	8	[pos]	2	[pos]	9

Supplementary Fig. 9. Assessment of seroconversion to vaccination in **ANP32A$^{N129I\text{-}D130N}$ chickens**.

Enzyme-linked immunosorbent assay (ELISA) was performed on serum samples obtained from the WT and ANP32A$^{N129I\text{-}D130N}$ chickens at 36-weeks of age to assess their response to routine vaccination. WT and ANP32A$^{N129I\text{-}D130N}$ chickens displayed similar serological profile. AE – Avian Encephalomyelitis; CAV – Chicken Anaemia Virus; EDS – Egg Drop Syndrome; IBD – Infectious Bursal Disease; IBV – Infectious Bronchitis Virus; ILT – Infectious Laryngotracheitis; NDV – Newcastle Disease Virus. pos – Positive; neg – Negative; Gp – Group. Sample Number 1 – 7 are ANP32A$^{N129I\text{-}D130N}$ chickens. Sample Number 8 – 13 are WT chickens.

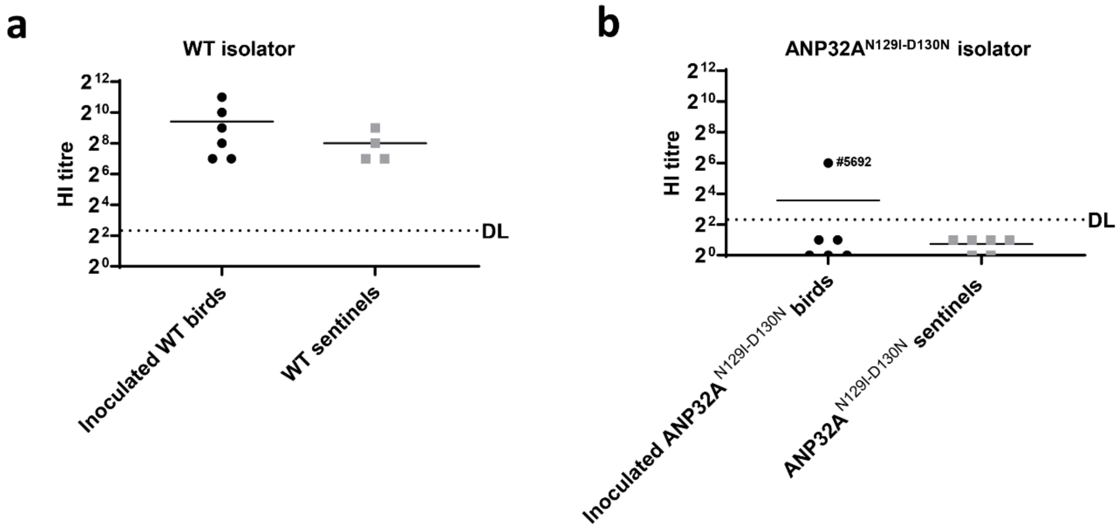

Supplementary Fig. 10. Detection of IAV antibodies in serum samples from birds infected with low-dose H9N2-UDL virus.

Haemagglutination-inhibition (HI) assays were performed on sera from all birds on day 14 post-inoculation to determine the presence of haemagglutinin-neutralising antibodies to H9N2-UDL virus mean and standard deviation indicated. *DL – detection limit of 5 HI units for HI assay. HI assays were not performed for two WT sentinels due to degradation of the collected blood samples.*

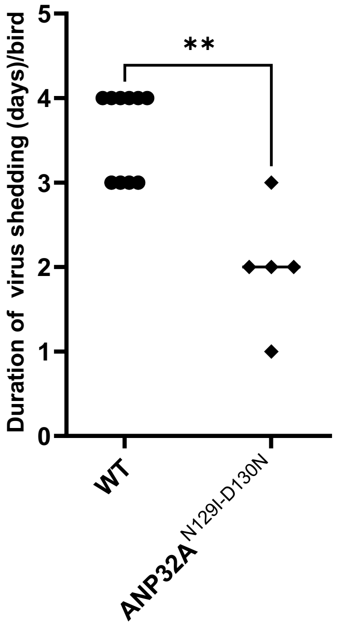

Supplementary Fig. 11. Duration of virus shedding from high-dose challenged chickens.

Each data point represents a directly inoculated bird showing the number of days that infectious virus was detectable in its oropharyngeal cavity. Only the 5 ANP32A$^{N129I\text{-}D130N}$ birds that were infected are represented in this analysis. Comparison of WT and ANP32A$^{N129I\text{-}D130N}$ data was performed using Mann-Whitney test. ** =P≤0.01.

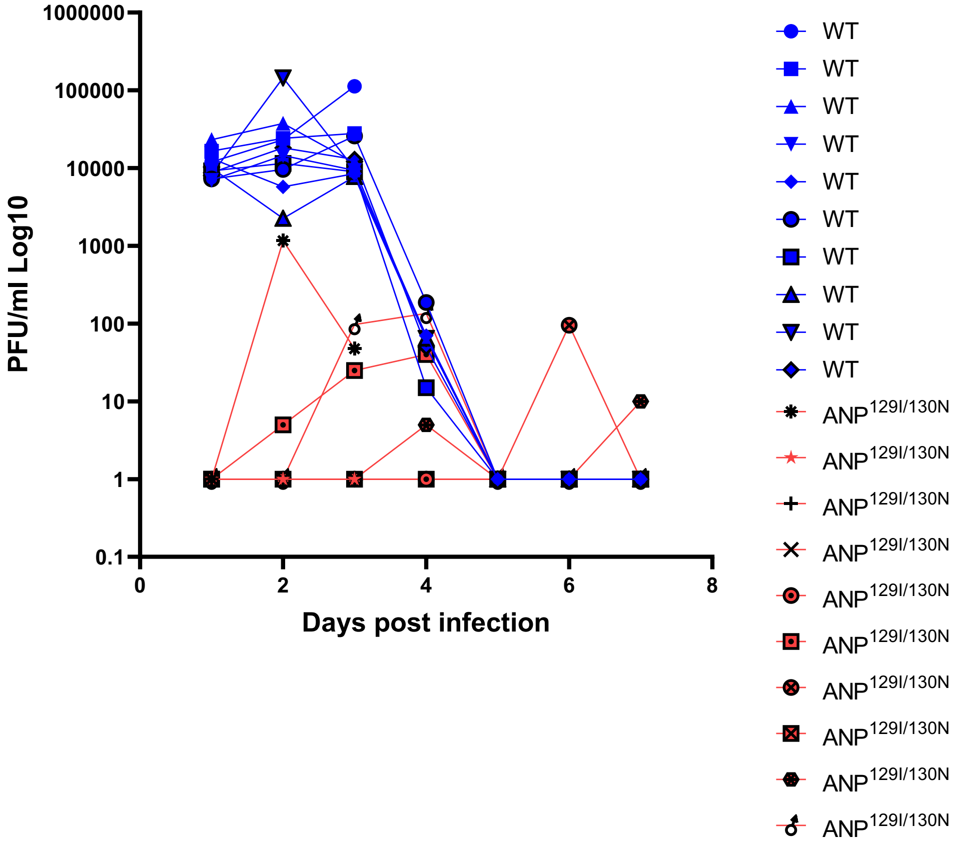

Supplementary Fig. 12. Infectious virus titre in individual birds inoculated with high-dose avian H9N2-UDL virus.

WT refers to wildtype chickens. *ANP$^{129I/130N}$* refers to ANP32A$^{N129I\text{-}D130N}$ chickens.

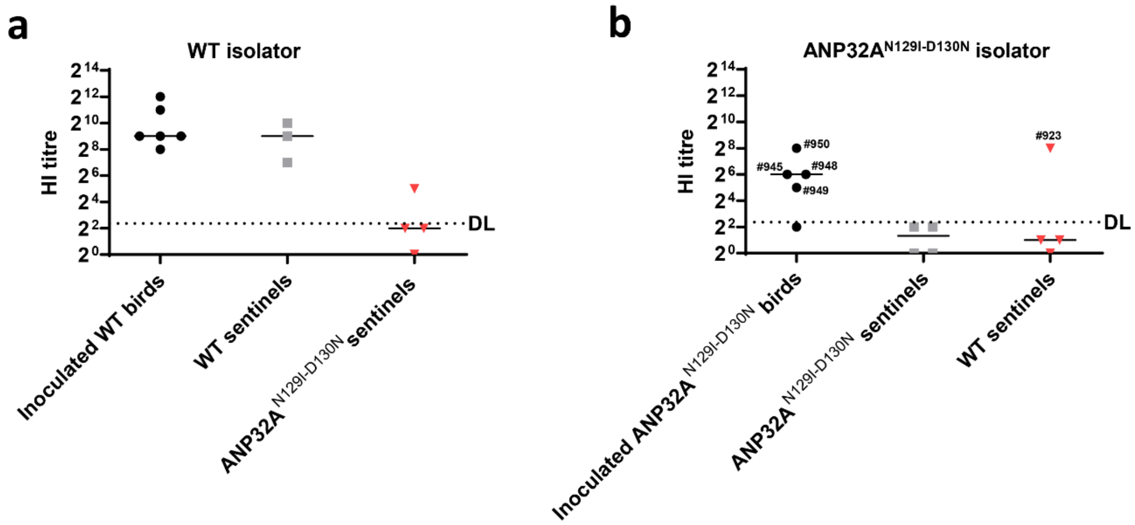

Supplementary Fig. 13. Detection of IAV antibodies in serum samples from birds infected with high-dose H9N2-UDL virus.

Haemagglutination-inhibition (HI) assays were performed on sera from all birds on day 14 post-inoculation to determine the presence of haemagglutinin-neutralising antibodies to the H9N2-UDL virus, mean and standard deviation indicated. *DL – detection limit of 5 HI units for HI assay. HI assays were not performed for one WT sentinel in the WT isolator and one directly inoculated ANP32A$^{N129I\text{-}D130N}$ bird in the ANP32A$^{N129I\text{-}D130N}$ isolator due to degradation of the collected blood samples.*

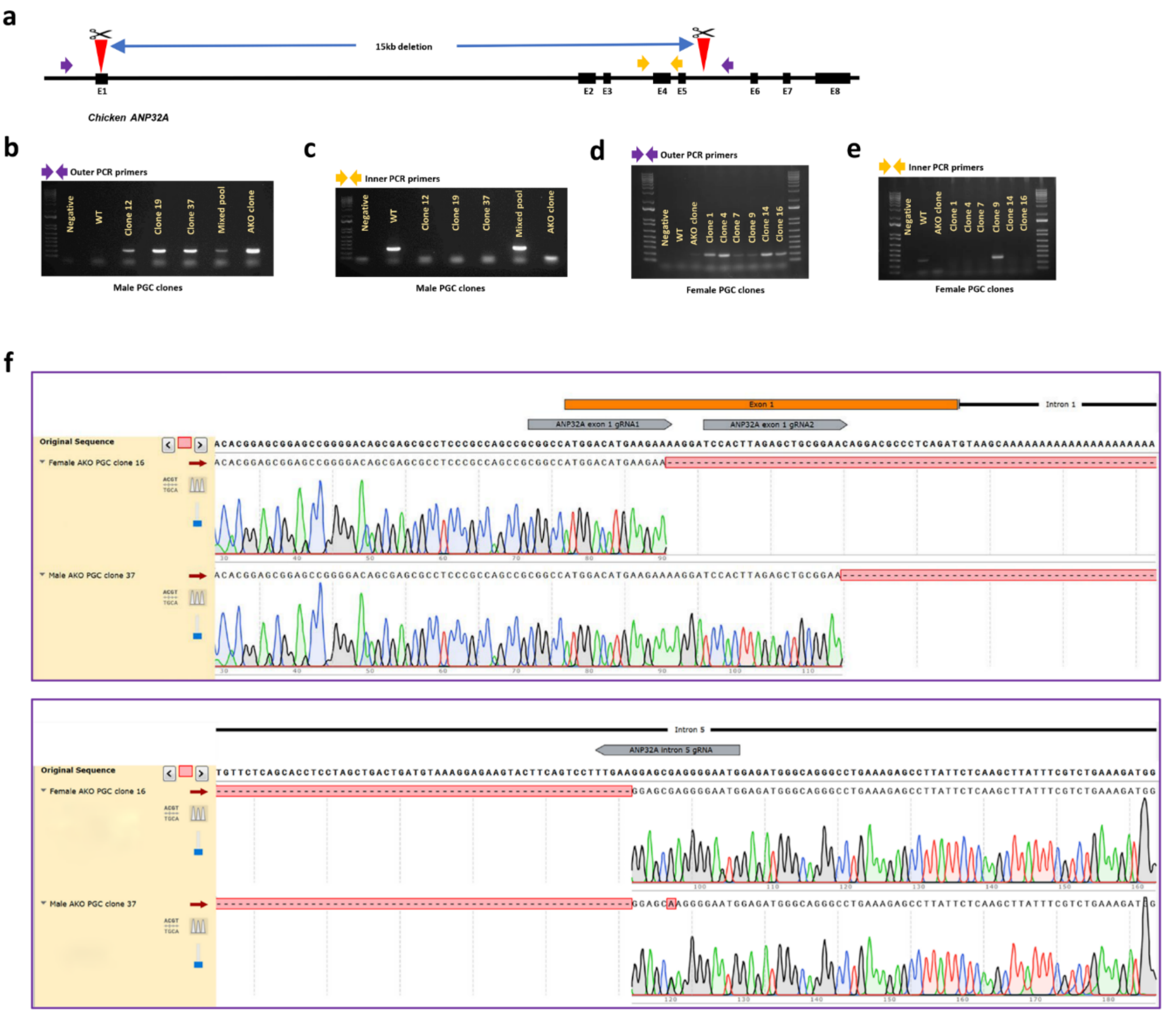

Supplementary Fig. 14. Generation of ANP32A-knockout (AKO) chickens.

A male and female PGC line were co-transfected with PX459 plasmid expressing gRNAs targeting exon 1 and intron 5 of ANP32A (**a**), puromycin resistance gene and Cas9. Female PGCs were targeted with ANP32A exon 1 gRNA1 and Intron 5 gRNA (**Supplementary Table 3**). Male PGCs were targeted with ANP32A exon 1 gRNA2 and Intron 5 gRNA (**Supplementary Table 3**) to generate a post-deletion product that is 17bp larger than in the targeted female PGCs. Transfected PGCs were puromycin-selected and then single cell cultures were established from targeted PGC pools to establish clonal populations of male and female AKO PGCs which were used to generate AKO chickens.

b,d, Deletion of the 15kb region in ANP32A in isolated PGC clones was assessed by PCR amplification using primers binding outside the deleted region. PCR amplification of wildtype genomic DNA fails due to the extremely large size of the predicted amplicon.

c,e, Gene deletion in the isolated PGC clones was further confirmed by PCR amplification using primers binding inside the deleted region.

f, Sanger sequencing of PCR products from AKO PGCs confirmed CRISPR-mediated deletions in ANP32A.

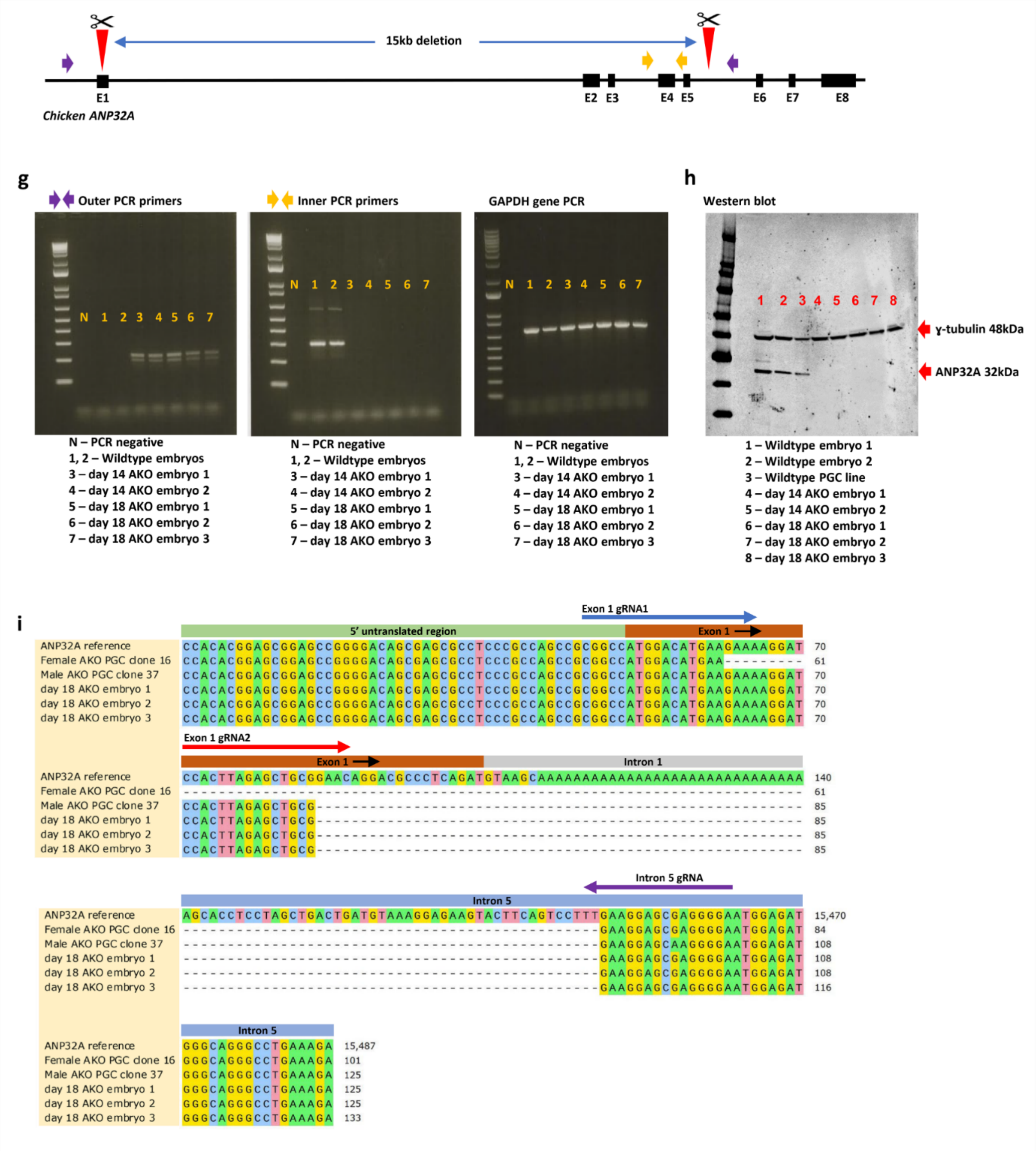

Supplementary Fig. 14 continued. Generation of ANP32A-knockout (AKO) chickens.

g, Confirmation of the deletion of the 15kb region in ANP32A in AKO embryos was assessed by PCR amplification of purified genomic DNA using primers binding outside the deleted region. Two PCR bands of slightly different sizes are observed in AKO embryos indicative of allelic contribution from sire and dam. Gene deletion was further confirmed by PCR amplification using primers binding inside the deleted region. PCR amplification of the GAPDH gene confirmed the integrity of embryo DNA isolated for PCR analysis.

h, Western blot analysis was performed to confirm loss of ANP32A expression in AKO embryos. Detection of ɣ-tubulin expression was used as a loading control.

i, Sanger sequencing of PCR products from AKO embryos confirmed CRISPR-mediated deletions in ANP32A.

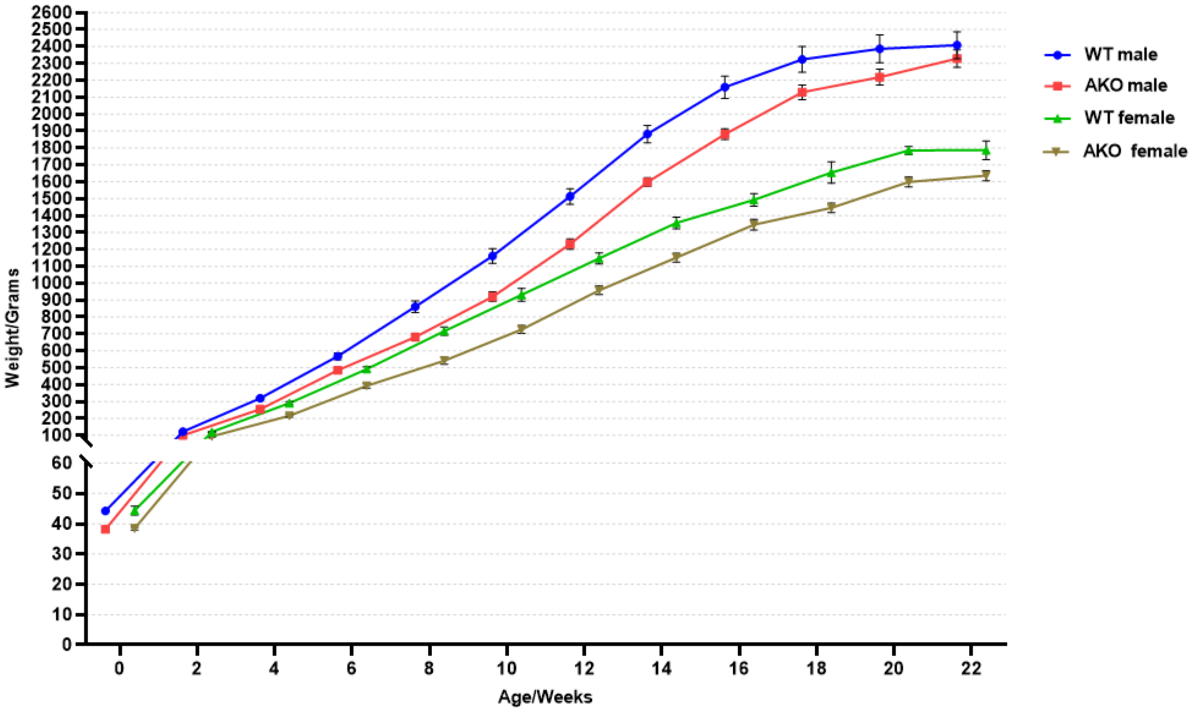

Age (weeks)	WT male vs AKO male T-test p-values	WT Female vs AKO female T-test p-values
0	0.000016 (****=P≤0.0001)	0.000541 (***=P≤0.001)
2	0.001006 (***=P≤0.001)	0.000146 (****=P≤0.0001)
4	0.000093 (****=P≤0.0001)	0.000074 (****=P≤0.0001)
6	0.002457 (**=P≤0.01)	0.000511 (***=P≤0.001)
8	0.000893 (***=P≤0.001)	0.000102 (***=P≤0.001)
10	0.000607 (***=P≤0.001)	0.000286 (***=P≤0.001)
12	0.000299 (***=P≤0.001)	0.000490 (***=P≤0.001)
14	0.000416 (***=P≤0.001)	0.000327 (***=P≤0.001)
16	0.003034 (**=P≤0.01)	0.010380 (**=P≤0.01)
18	0.052352 (*=P≤0.05)	0.004857 (**=P≤0.01)
20	0.113519 (ns)	0.000452 (***=P≤0.001)
22	0.437474 (ns)	0.020921 (*=P≤0.05)

Supplementary Fig. 15. Comparison of mean bodyweight (BW) between WT and AKO chickens.

WT birds: n= 6 females, 8 males; AKO birds: n= 9 females, 7 males. Error bars are ± standard error of the mean. Table shows p-values from unpaired T-test comparisons of mean bodyweights at various ages.

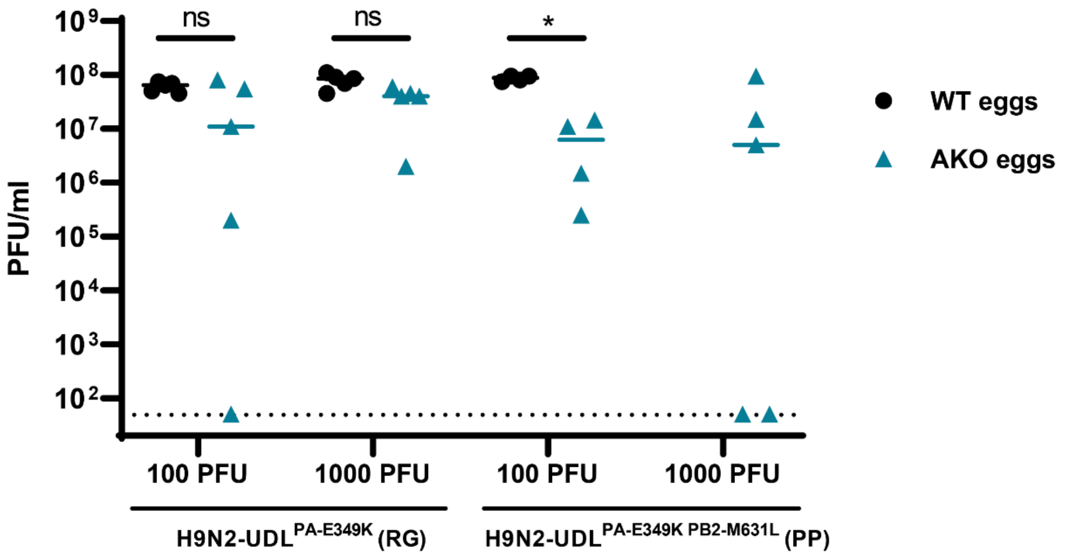

Supplementary Fig. 16.

WT or AKO 11-day-old embryonated eggs were inoculated with mutant H9N2-UDL virus containing a single PA-E349K substitution isolated through reverse genetics (RG) or the double mutant variant containing the PA-E349K and PB2-M631L substitutions isolated through plaque purification (PP). The inoculated eggs were incubated at 37.5°C. Allantoic fluids were collected 48 hours later and PFU/ml measured by plaque assay. DL – detection limit of plaque assay (10 PFU/ml). Data were analysed by unpaired T-test of transformed data (Y=Log(Y)). ns=not significant; ns= not significant; *=P≤0.05.

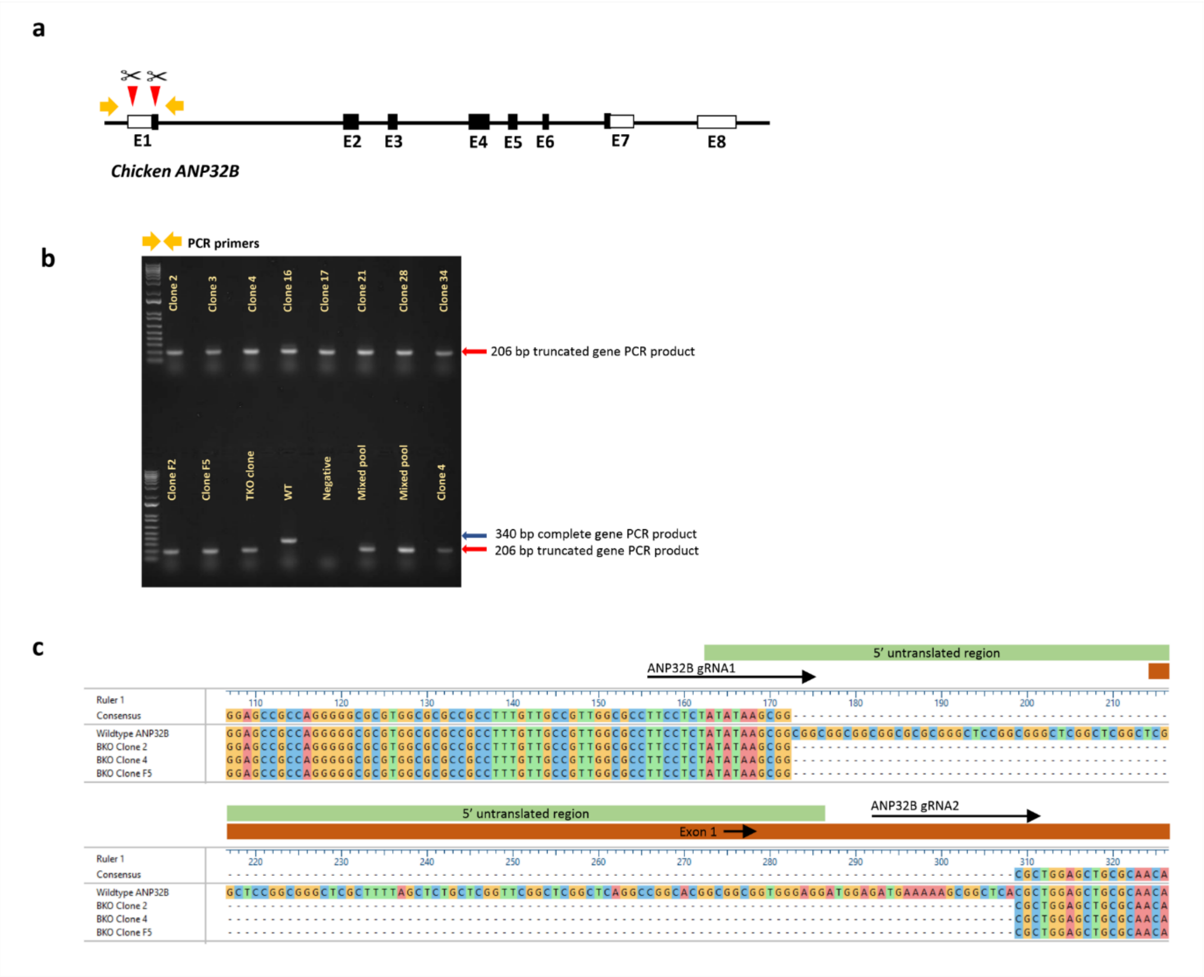

Supplementary Fig. 17. Generation of ANP32 knockout cells.

a,b,c, ANP32B-knockout (BKO) PGCs were generated by using two gRNAs to target the promoter region and exon 1 of ANP32B to create a 134-bp loss-of-function deletion. Clonal populations of targeted PGCs were isolated and screened by PCR and Sanger sequencing to confirm deletions.

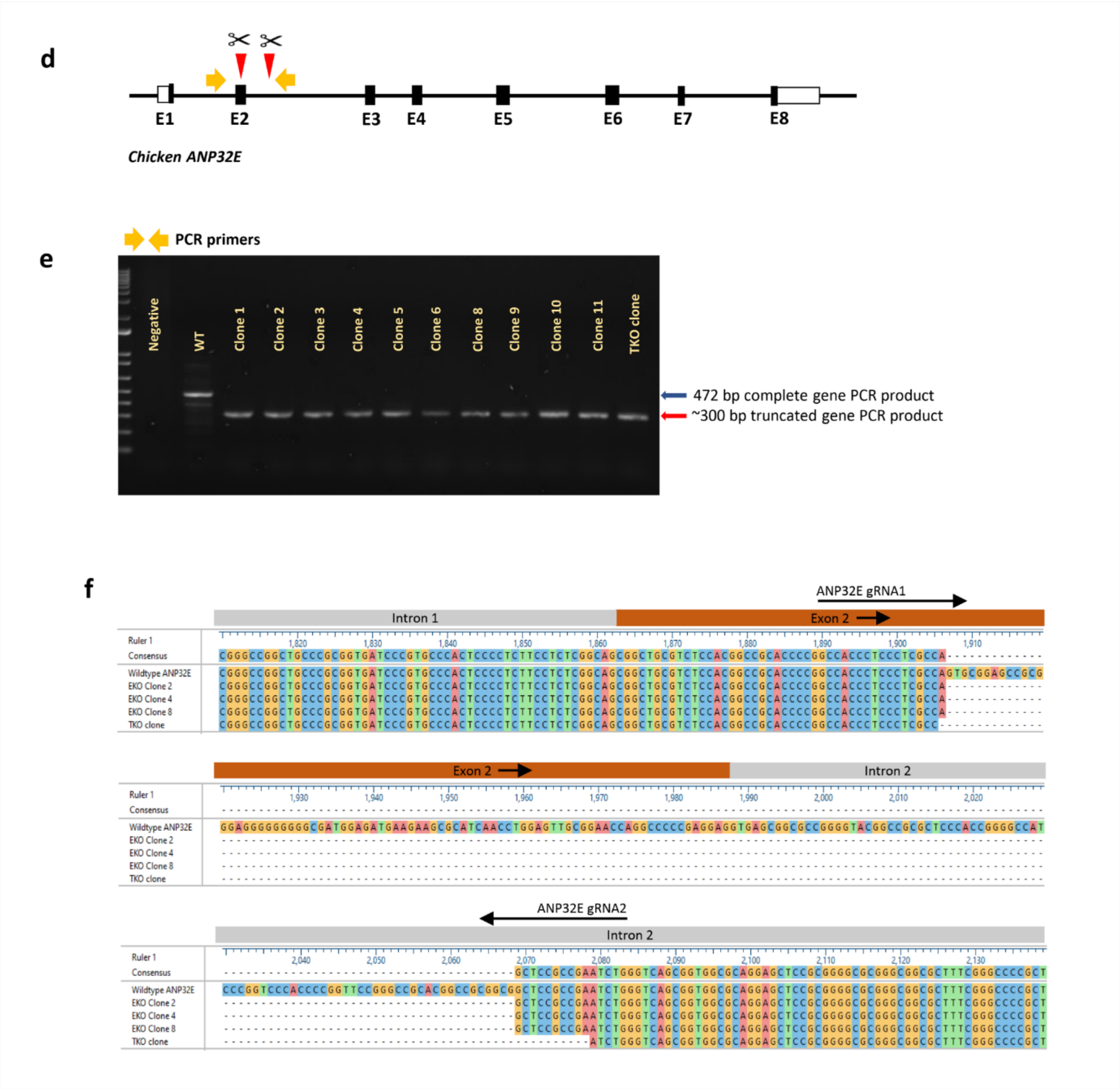

Supplementary Fig. 17 continued. Generation of ANP32 knockout cells.

d,e,f, ANP32E-knockout (EKO) PGCs were generated by using two gRNAs to target exon 2 and intron 2 of ANP32E to create a 160-bp loss-of-function deletion. Clonal populations of targeted PGCs were isolated and screened by PCR and Sanger sequencing to confirm deletions in homozygous gene-edited PGCs.

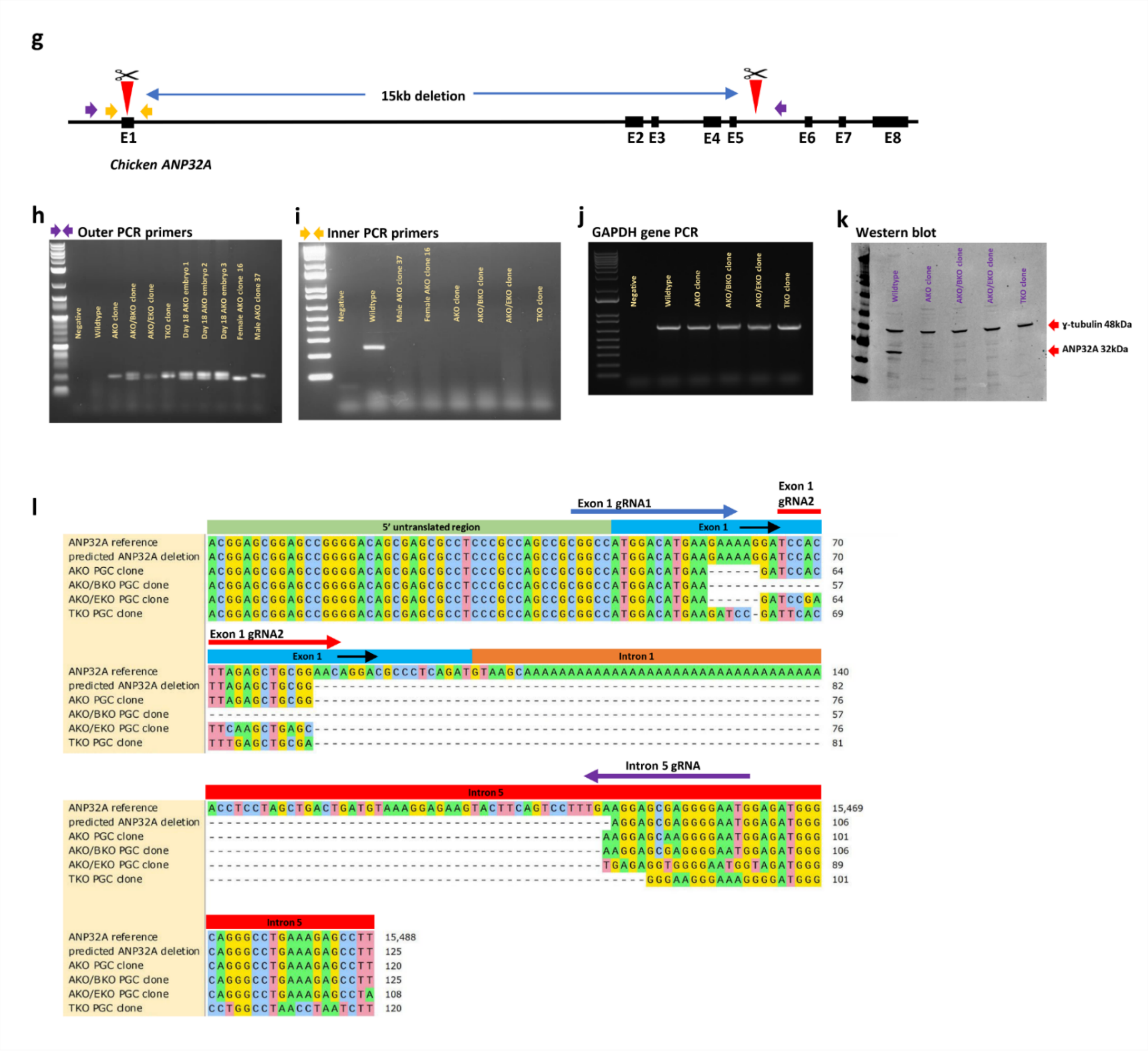

Supplementary Fig. 17 continued. Generation of ANP32 knockout cells.

g, To generate the AKO/BKO (containing concurrent loss-of-function deletions in ANP32A and ANP32B) and AKO/EKO genotypes (containing concurrent loss-of-function deletions in ANP32A and ANP32E), BKO PGCs and EKO PGCs were targeted using CRISPR/Cas9 vectors to create a 15-kb deletion using gRNAs targeting exon 1 and intron 5 of ANP32A. To generate the TKO genotype (containing concurrent loss-of-function deletions in ANP32A, ANP32B and ANP32E), AKO/BKO PGCs were transfected with CRISPR/Cas9 vectors to target exon 2 and intron 2 of ANP32E. Single cell cultures were established from targeted PGCs to isolate clonal populations of homozygous gene-edited PGCs.

h, Deletion of the 15kb region in ANP32A was assessed by PCR amplification using primers binding outside the deleted region.

i, Gene deletion was further confirmed by PCR amplification using primers binding inside the deleted region.

j, PCR amplification of the GAPDH gene confirmed the integrity of DNA isolated for PCR analysis.

k, Western blot analysis was performed to confirm loss of ANP32A expression in the isolated ANP32-knockout cells. ɣ-tubulin expression detection was used as a loading control.

l, Sanger sequencing of PCR products from ANP32 knockout cells confirmed CRISPR-mediated deletions in ANP32A.
