# Supplementary material for: Creating resistance to avian influenza infection through genome editing of the ANP32 gene family
Source: Nat Commun. 2023 Oct 10;14:6136. doi: 10.1038/s41467-023-41476-3 (PMC10564915; doi:10.1038/s41467-023-41476-3)
Supplement: Supplementary file 2 — Reporting Summary [file 41467_2023_41476_MOESM2_ESM.pdf]

nature research

Corresponding author(s): Mike McGrew

Last updated by author(s): Aug 21, 2023

Reporting Summary

Nature Research wishes to improve the reproducibility of the work that we publish. This form provides structure for consistency and transparency in reporting. For further information on Nature Research policies, see our Editorial Policies and the Editorial Policy Checklist.

Statistics

For all statistical analyses, confirm that the following items are present in the figure legend, table legend, main text, or Methods section.

n/a	Confirmed	
☐	☒	The exact sample size (*n*) for each experimental group/condition, given as a discrete number and unit of measurement
☐	☒	A statement on whether measurements were taken from distinct samples or whether the same sample was measured repeatedly
☐	☒	The statistical test(s) used AND whether they are one- or two-sided<br>*Only common tests should be described solely by name; describe more complex techniques in the Methods section.*
☐	☒	A description of all covariates tested
☐	☒	A description of any assumptions or corrections, such as tests of normality and adjustment for multiple comparisons
☐	☒	A full description of the statistical parameters including central tendency (e.g. means) or other basic estimates (e.g. regression coefficient) AND variation (e.g. standard deviation) or associated estimates of uncertainty (e.g. confidence intervals)
☐	☒	For null hypothesis testing, the test statistic (e.g. *F*, *t*, *r*) with confidence intervals, effect sizes, degrees of freedom and *P* value noted<br>*Give P values as exact values whenever suitable.*
☒	☐	For Bayesian analysis, information on the choice of priors and Markov chain Monte Carlo settings
☒	☐	For hierarchical and complex designs, identification of the appropriate level for tests and full reporting of outcomes
☒	☐	Estimates of effect sizes (e.g. Cohen's *d*, Pearson's *r*), indicating how they were calculated

Our web collection on statistics for biologists contains articles on many of the points above.

Software and code

Policy information about availability of computer code

Data collection	Data collection for polymerase assays was performed using WinGlow sofware (Berthold) for the MicroLumat Plus LB 96V luminometer (Berthold) or a FLUOstar Omega plate reader (BMG Labtech).<br>Data for western blot was collected using Image Studio Ver 5.2 software with LI-COR Odyssey machine.<br>RNA sequencing data for PGC transcriptome analysis was collected using Illumina Novaseq 6000 system by Beijing Genomics Institute.<br>Viral sequencing data was generated from pooled libraries sequenced on a 2x300cycle MiSeq Reagent Kit v2 (Illumina, USA).<br>PCR gel electrophoresis data was collected using the NuGenius Gel documentation system (Syngene).
Data analysis	Statistical analysis and data visualization was performed using Prism 9 v9.2.0 or v9.4.0 (GraphPad).<br>Visualization and alignment of Sanger sequencing chromatograms was performed using SeqMan Pro 17 (Lasergene 17, DNASTAR).<br>Alignment of Sanger sequencing sequences was performed using MegAlign Pro 17 (Lasergene 17, DNASTAR).<br>Viral sequencing data was analysed using Geneious Prime 2019 software (Dotmatics).<br>Location of amino acids in symmetric and assymetric polymerase dimers were plotted using ChimeraX 1.3 (UCSF).<br>For RNA sequencing analysis of PGCs, initial analysis was conducted by BGI using SOAPnuke software (Beijing Genomics Institute).<br>Quality-control processing and analysis of FASTQ files were performed in the CLC Genomics Workbench v20.0.4 (Qiagen).<br>Alignment and quantitative analysis of RNA expression was performed using the 'RNA-Seq Analysis' tool of the CLC Genomics Workbench (CLC Bio v2.18).<br>Differential expression within the RNA-Seq data was analysed using the 'Differential Expression for RNA-Seq' tool of the CLC Genomics Workbench (CLC Bio v2.2).<br>HEAT Maps were generated using the 'Create HEAT Map for RNA-Seq' tool in the CLC Genomics Workbench (CLC Bio v2.2).<br>Principal component analysis (PCA) plots were generated using the 'PCA for RNA-Seq' tool in the CLC Genomics Workbench (CLC Bio v2.2).

For manuscripts utilizing custom algorithms or software that are central to the research but not yet described in published literature, software must be made available to editors and reviewers. We strongly encourage code deposition in a community repository (e.g. GitHub). See the Nature Research guidelines for submitting code & software for further information.

Data

Policy information about availability of data

All manuscripts must include a data availability statement. This statement should provide the following information, where applicable:

- Accession codes, unique identifiers, or web links for publicly available datasets
- A list of figures that have associated raw data
- A description of any restrictions on data availability

The data supporting the findings of this study are available within the article and its Supplementary Information. The source data for the main figures and extended data figures are provided as Source Data files. Illumina RNA sequencing data for PGC transcriptome analysis are deposited in the GEO and SRA archives at NCBI (Accession number GSE182397). The authors declare that all unique materials used are readily available from the authors upon MTA agreement.
The authors declare that all unique materials used are readily available from the authors upon MTA agreement.

Field-specific reporting

Please select the one below that is the best fit for your research. If you are not sure, read the appropriate sections before making your selection.

[x] Life sciences [ ] Behavioural & social sciences [ ] Ecological, evolutionary & environmental sciences

For a reference copy of the document with all sections, see nature.com/documents/nr-reporting-summary-flat.pdf

Life sciences study design

All studies must disclose on these points even when the disclosure is negative.

Sample size	We used the minimum amount of animals allowed for the generation of statistically significant conclusions. We performed a power calculation to determine the group sizes that we required to see a biologically relevant difference in viral shedding (area under the curve) between the WT and GE ANP32a chickens. From our previous work we have information about the average area under the curve of virus shedding using the low pathogenicity H9N2 influenza virus in chickens with a WT ANP32a protein and have a measure of the variability seen (Area under the curve =5.471 log10 plaque forming units (pfu) with a standard deviation of 0.24). From the literature we can see multiple examples of experimental analysis of the infectious dose 50 (ID50) of avian influenza virus for chickens, the median being approximately 103 plaque forming units (1-3). A reduction of viral shedding to this level (the area under the curve estimated as 3.506 log10 pfu) therefore would correspond to reduce the ability of the virus to replicate in the chicken host and also onwards transmission. Therefore we used a t-test power analysis to determine the group size required to give a significance level of 0.05 and a power of 85%. The group size was determine as 8 experimental units per group and therefore if each chicken is an experimental unit 8 chickens as directly inoculated and 8 as in-contact recipients were required. We require 4 additional animals in both directly inoculated and in-contact groups to sacrifice at day 3 post inoculation and exposure to determine viral dissemination profile in the chickens.<br>(1.) Aldous EW, et al. 2010. Avian Pathol 39:265-273.<br>(2.) Pantin-Jackwood MJ, et al. 2017. Vet Res 48:33.<br>(3.) Swayne DE, Slemons RD. 2008. Avian Dis 52:455-460.
Data exclusions	No data was excluded.
Replication	Bird infection experiments were not replicated as n was sufficiently large to generate a statistical significance. Replication numbers are stated in the Figure Legends.
Randomization	Some birds were randomly culled for post mortem analysis on day 3 post-inoculation.
Blinding	Bird infection experiments were blinded such that bird inoculation and collection of oropharyngeal swabs were performed by other researchers while the authors conducted blind analysis of the oropharyngeal swabs.

Reporting for specific materials, systems and methods

We require information from authors about some types of materials, experimental systems and methods used in many studies. Here, indicate whether each material, system or method listed is relevant to your study. If you are not sure if a list item applies to your research, read the appropriate section before selecting a response.

Materials & experimental systems

n/a	Involved in the study	
	x	Antibodies
	x	Eukaryotic cell lines
x		Palaeontology and archaeology
	x	Animals and other organisms
x		Human research participants
x		Clinical data
x		Dual use research of concern

Methods

n/a	Involved in the study	
x		ChIP-seq
x		Flow cytometry
x		MRI-based neuroimaging

Antibodies

Antibodies used	Rabbit anti-ANP32A antibody (Sigma-Aldrich AV40203).<br>Mouse anti-ɣ-tubulin antibody (Sigma-Aldrich TS6557).<br>IRDye 800CW goat anti-mouse secondary antibody (LI-COR 925-32211).<br>IRDye 680RD goat anti-rabbit secondary antibody (LI-COR 925-68070).
Validation	Rabbit anti-ANP32A antibody (Sigma-Aldrich AV40203) was validated for western blot in chicken cells in Long et al., 2019 eLife (https://doi.org/10.7554/eLife.45066) and with the data provided in this manuscript.<br>Mouse anti-ɣ-tubulin antibody (Sigma-Aldrich TS6557) was validated by the manufacturer for western blot detection of chicken ɣ-tubulin.<br>IRDye 800CW goat anti-mouse secondary antibody (LI-COR) was validated by LI-COR for detection of mouse immunoglobulins in western blot assays.<br>IRDye 680RD goat anti-rabbit secondary antibody (LI-COR) was validated by LI-COR for detection of rabbit immunoglobulins in western blot assays.

Eukaryotic cell lines

Policy information about cell lines

Cell line source(s)	Chicken primordial germ cells (PGCs) were derived from fertile eggs obtained from commercial Hy-line layer flocks bred at the National Avian Research Facility, Midlothian, United Kingdom. PGC cell lines consistently tested negative for Mycoplama. Fibroblast-like cells were derived from these PGCs.<br>ANP32A-ANP32B-double-knockout human EHAP1 cells were supplied by Horizon Discovery.<br>Madin-Darby canine kidney cells (ATCC) were obtained commercially.<br>Human airway epithelial cells were purchased from Epithelix Sarl, Inc.
Authentication	All cell lines were commonly identified by microscopy. Chicken PGCs, PGC-derived fibroblast-like cells and human EHAP1 cells were validated by Sanger sequencing and western blot analysis of ANP32A.
Mycoplasma contamination	PGC lines were regulaly tested for mycoplasma. Human airway epithelial cells were tested by the company for mycoplasma.
Commonly misidentified lines<br>(See ICLAC register)	No commonly misindentified cell lines were used in this study.

Animals and other organisms

Policy information about studies involving animals; ARRIVE guidelines recommended for reporting animal research

Laboratory animals	Commercial Hy-line layer flocks bred to produce fertile eggs at the National Avian Research Facility (NARF), Midlothian, United Kingdom were housed under standard conditions (feeding ad libitum)n accordance with the relevant regulations of the UK Home Office, the Roslin Institute Animal Welfare and Ethical Review Board (AWERB) Committee, GM and Biological Safety Committee of the University of Edinburgh and the Pirbright Institute.<br>For bird infections, mixed sex wildtype and genome-edited Hy-line layer chickens were hatched at the NARF and transported at 1 day of age to the Pirbright Institute, Surrey, UK in accordance with relevant regulations of the UK Home Office, the Roslin Institute Animal Welfare and Ethical Review Board (AWERB) Committee, GM and Biological Safety Committee of the University of Edinburgh and the Pirbright Institute.<br>At the Pirbright Institute, chickens were raised to two weeks of age under standard conditions approved by the UK Home Office and the Pirbright Institute.<br>For animal infection experiments, 2-week old chickens were housed in negative pressured BioFlex® B50 Rigid Body Poultry isolators (Bell Isolation Systems) under standard conditions approved by the UK Home Office and the Pirbright Institute.
Wild animals	No wild animals were used in this study.

Field-collected samples	No field collected samples were used.
Ethics oversight	All experimental protocols and studies were performed with the approval of the Roslin Institute Animal Welfare and Ethical Review Board (AWERB) Committee and the Pirbright Animal Welfare and Ethics Review Board (AWERB).

Note that full information on the approval of the study protocol must also be provided in the manuscript.
