# Supplementary material for: Creating resistance to avian influenza infection through genome editing of the ANP32 gene family
Source: Nat Commun. 2023 Oct 10;14:6136. doi: 10.1038/s41467-023-41476-3 (PMC10564915; doi:10.1038/s41467-023-41476-3)
Supplement: Supplementary file 4 — Source Data [file 41467_2023_41476_MOESM4_ESM.zip › SOURCE DATA/SUPPLEMENTARY DATA/Supplementary Figure 2_Sanger-sequencing_of_PCR-amplified_off-target_sites/Guidance.docx]

Off-target sequence ID

Lorna-09-06-2021-785_chr1_91872693

Lorna-09-06-2021-793_chr1_153558607

Lorna-14-06-2021-805_ chr4_25712226

Lorna-14-06-2021-813_ chr6_4920697

1 – FR3F-wild-type PGC

2 – FR6F-wild-type PGC

3 – FR3F-N129I-D130N-clone #3

4 – FR3F-N129I-D130N-clone #3

5 – FR3F-N129I-D130N-clone #3

6 – FR5M-N1291-D130N -clone #7

7 – FR6F-N129I-D130N-clone #10

8 – FR3F-N129I-D130N -clone #40
