# Supplementary material for: Creating resistance to avian influenza infection through genome editing of the ANP32 gene family
Source: Nat Commun. 2023 Oct 10;14:6136. doi: 10.1038/s41467-023-41476-3 (PMC10564915; doi:10.1038/s41467-023-41476-3)
Supplement: Supplementary file 4 — Source Data [file 41467_2023_41476_MOESM4_ESM.zip › SOURCE DATA/SUPPLEMENTARY DATA/Supplementary Figure 3/Supplementary Figure 3_FR3F clones_left-panel.pdf]

Image ID: 0011614_01
Acquire Time: 15-Nov-2019 14:44:59

Acquisition Information

#	Image ID	Acquire Time	Channels	Resolution	Intensities	Quality	Analysis	Image Name	Comment
1	0011614_01	15-Nov-2019 14:44:59	700 800	169um	5.0 5.0	lowest	Manual	0011614_01	

Image Display Values

Channel	Color	Minimum	Maximum	K
700	Gray Scale (Black on White)	1600	12600	0
800	Gray Scale (Black on White)	1240	13000	0

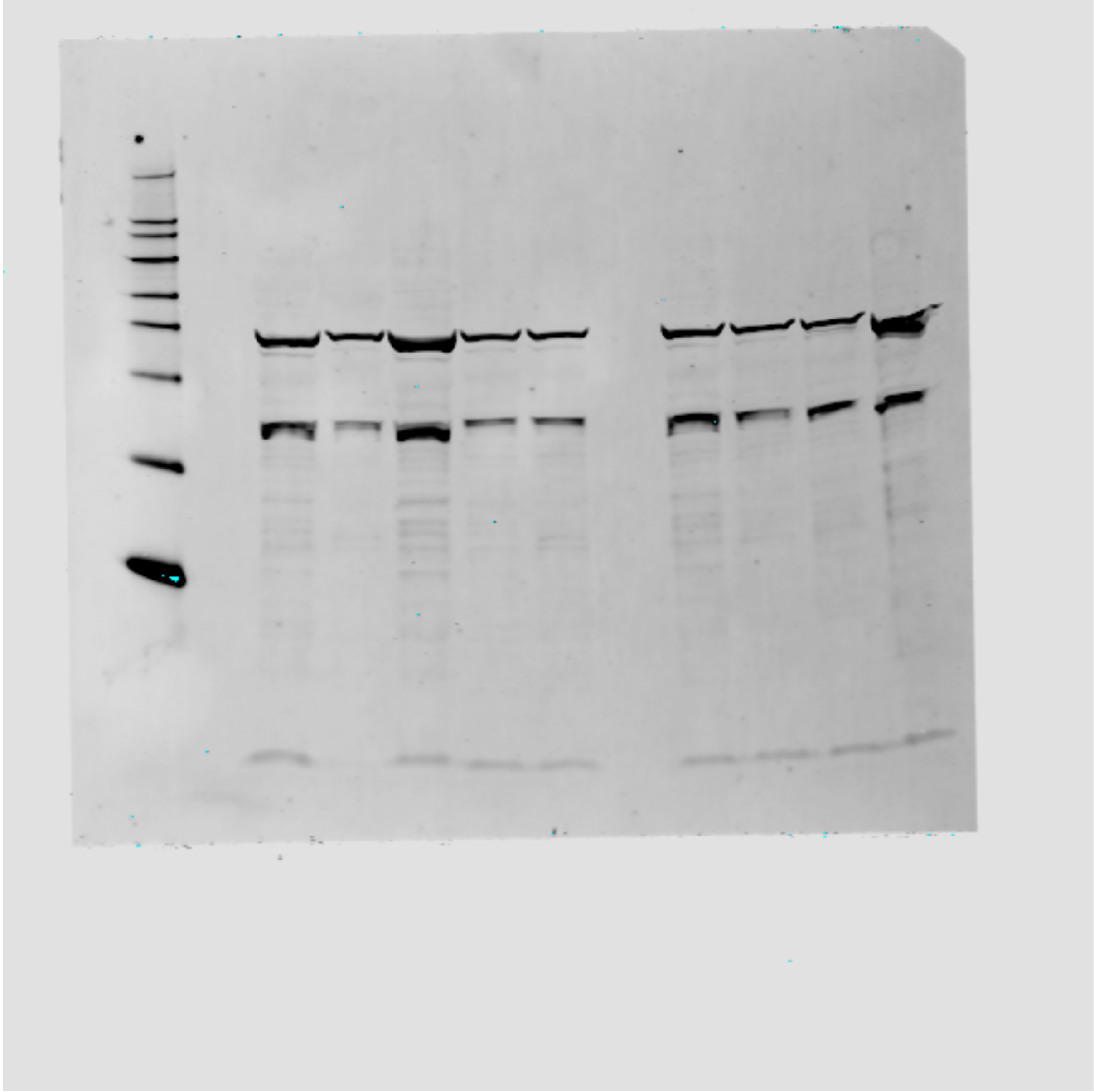

Acquisition Information (continued)

#	Image Modifications
1	
