# Supplementary material for: Creating resistance to avian influenza infection through genome editing of the ANP32 gene family
Source: Nat Commun. 2023 Oct 10;14:6136. doi: 10.1038/s41467-023-41476-3 (PMC10564915; doi:10.1038/s41467-023-41476-3)
Supplement: Supplementary file 4 — Source Data [file 41467_2023_41476_MOESM4_ESM.zip › SOURCE DATA/SUPPLEMENTARY DATA/Supplementary Figure 3/Supplementary Figure 3_FR5M_&_FR6F clones.pdf]

**Image ID: 0011616_02**
**Acquire Time: 15-Nov-2019 15:05:12**



## Acquisition Information

| # | Image ID | Acquire Time | Channels | Resolution | Intensities | Quality | Analysis | Image Name | Comment |
|---|---|---|---|---|---|---|---|---|---|
| 1 | 0011616_02 | 15-Nov-2019 15:05:12 | 700 800 | 169um | 5.0 5.0 | lowest | Manual | 0011616_02 | |

## Image Display Values

| Channel | Color | Minimum | Maximum | K |
|---|---|---|---|---|
| 700 | Gray Scale (Black on White) | 1510 | 6340 | 0 |
| 800 | Gray Scale (Black on White) | 956 | 9560 | 0 |

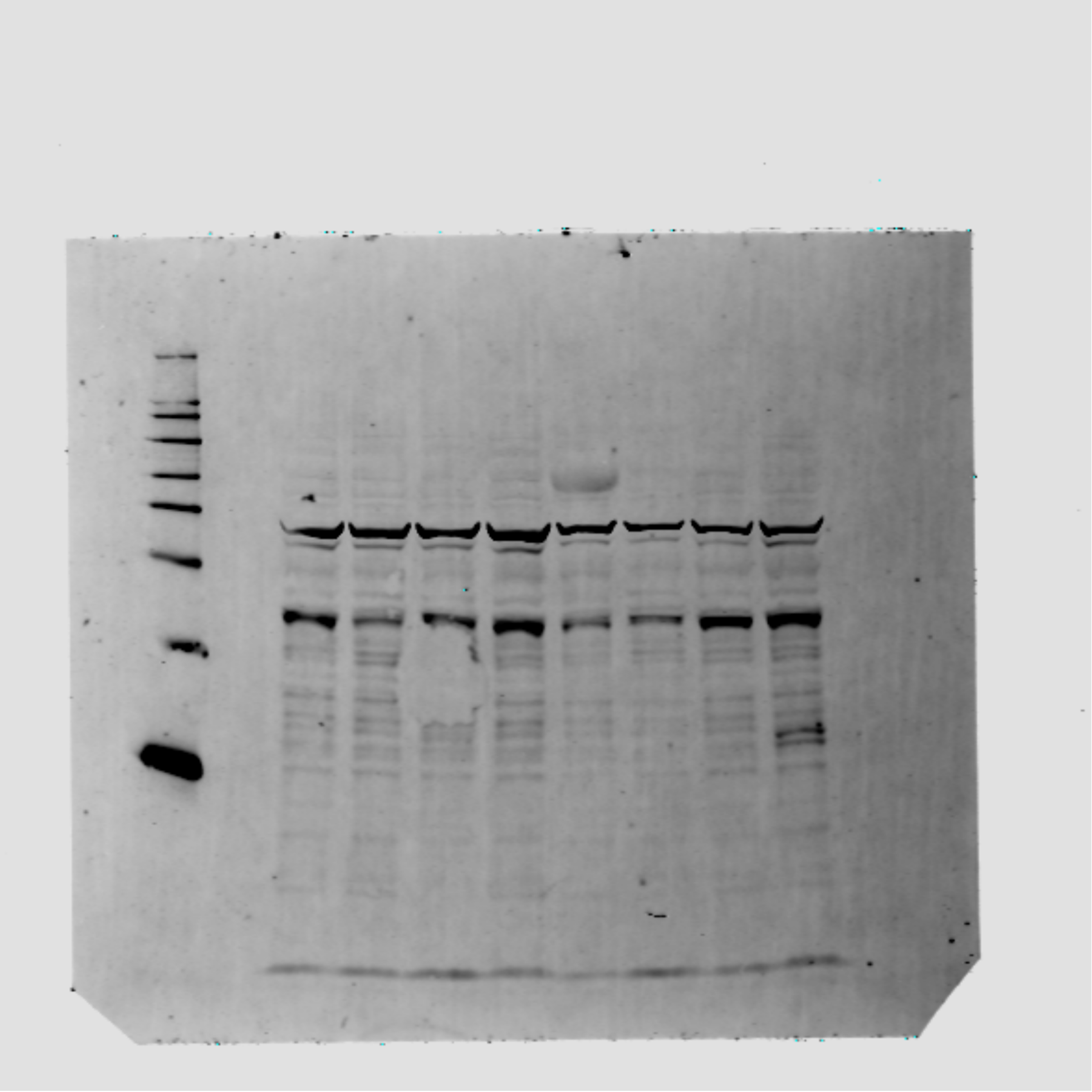

Acquisition Information (continued)

| # | Image Modifications |
| --- | --- |
| 1 | Flip Top to Bottom Image ID: 0011616_01 |
