# Supplementary figures and images for: Creating resistance to avian influenza infection through genome editing of the ANP32 gene family
Source: Nat Commun. 2023 Oct 10;14:6136. doi: 10.1038/s41467-023-41476-3 (PMC10564915; doi:10.1038/s41467-023-41476-3)

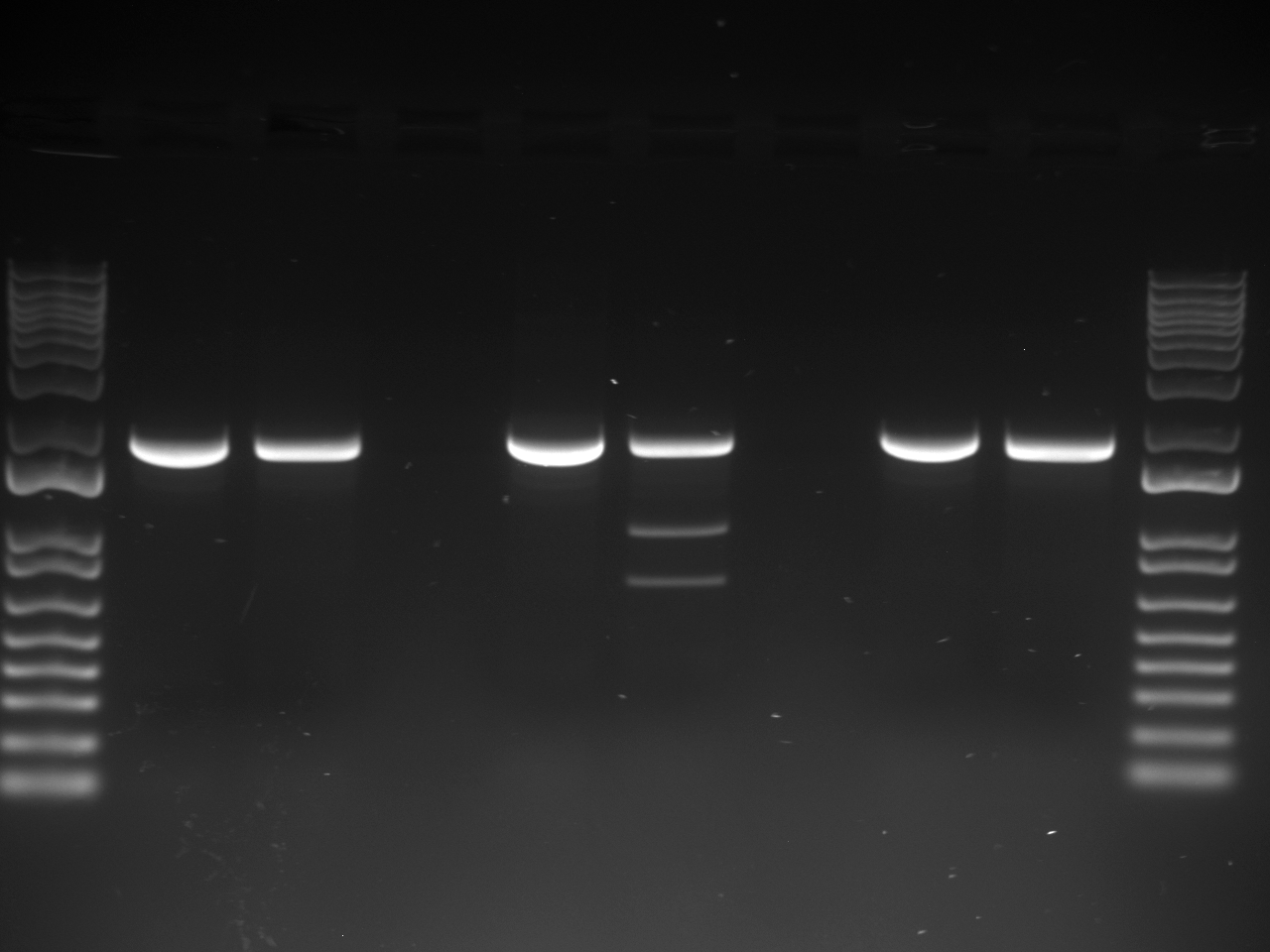

Supplement: Supplementary file 4 — Source Data [file 41467_2023_41476_MOESM4_ESM.zip › SOURCE DATA/SUPPLEMENTARY DATA/Supplementary Figure 1/Supplementary figure 1a_FR5M_mixed pool.tif]

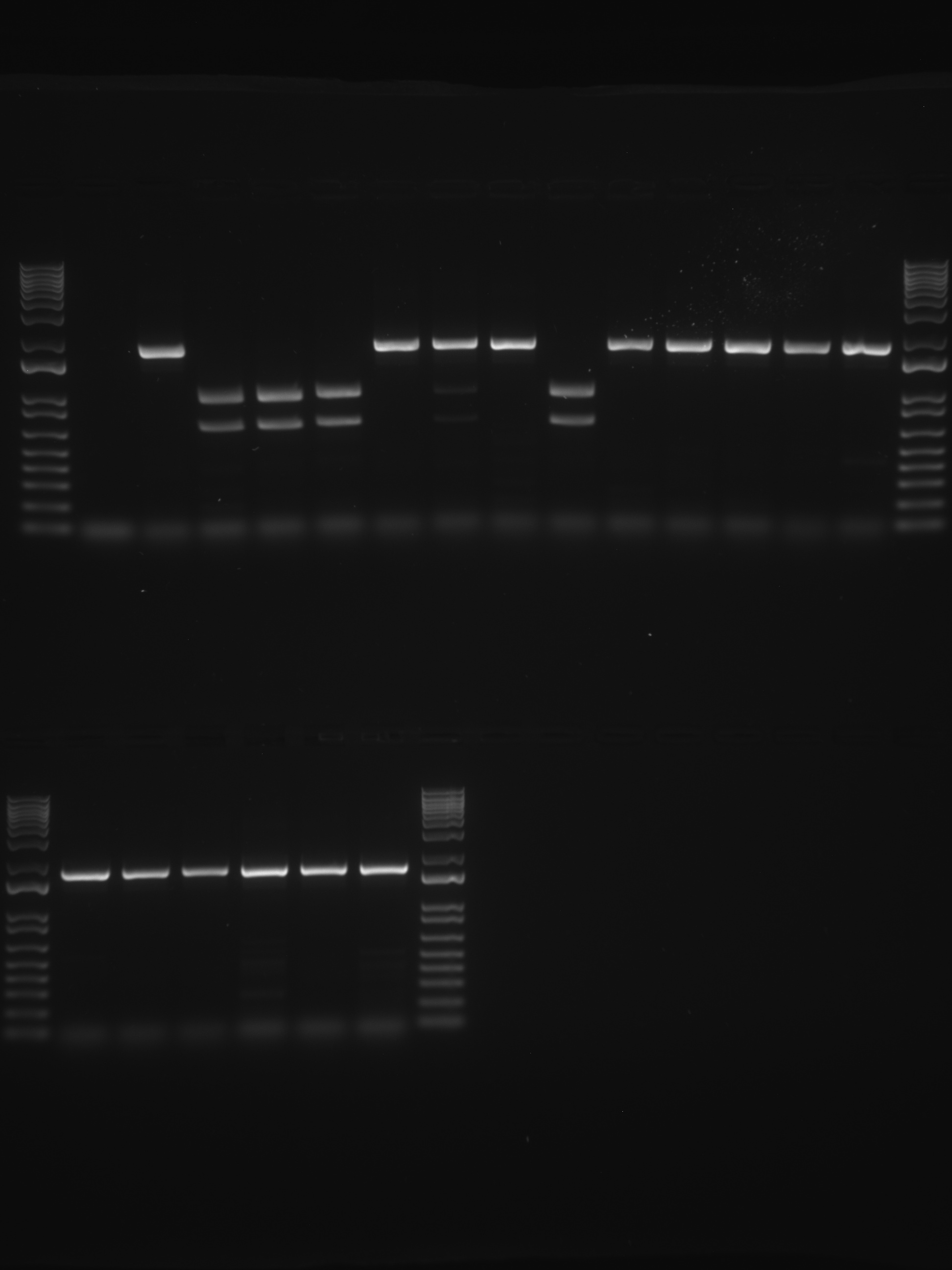

Supplement: Supplementary file 4 — Source Data [file 41467_2023_41476_MOESM4_ESM.zip › SOURCE DATA/SUPPLEMENTARY DATA/Supplementary Figure 1/Supplementary figure 1b_FR5M_top-panel.jpg]

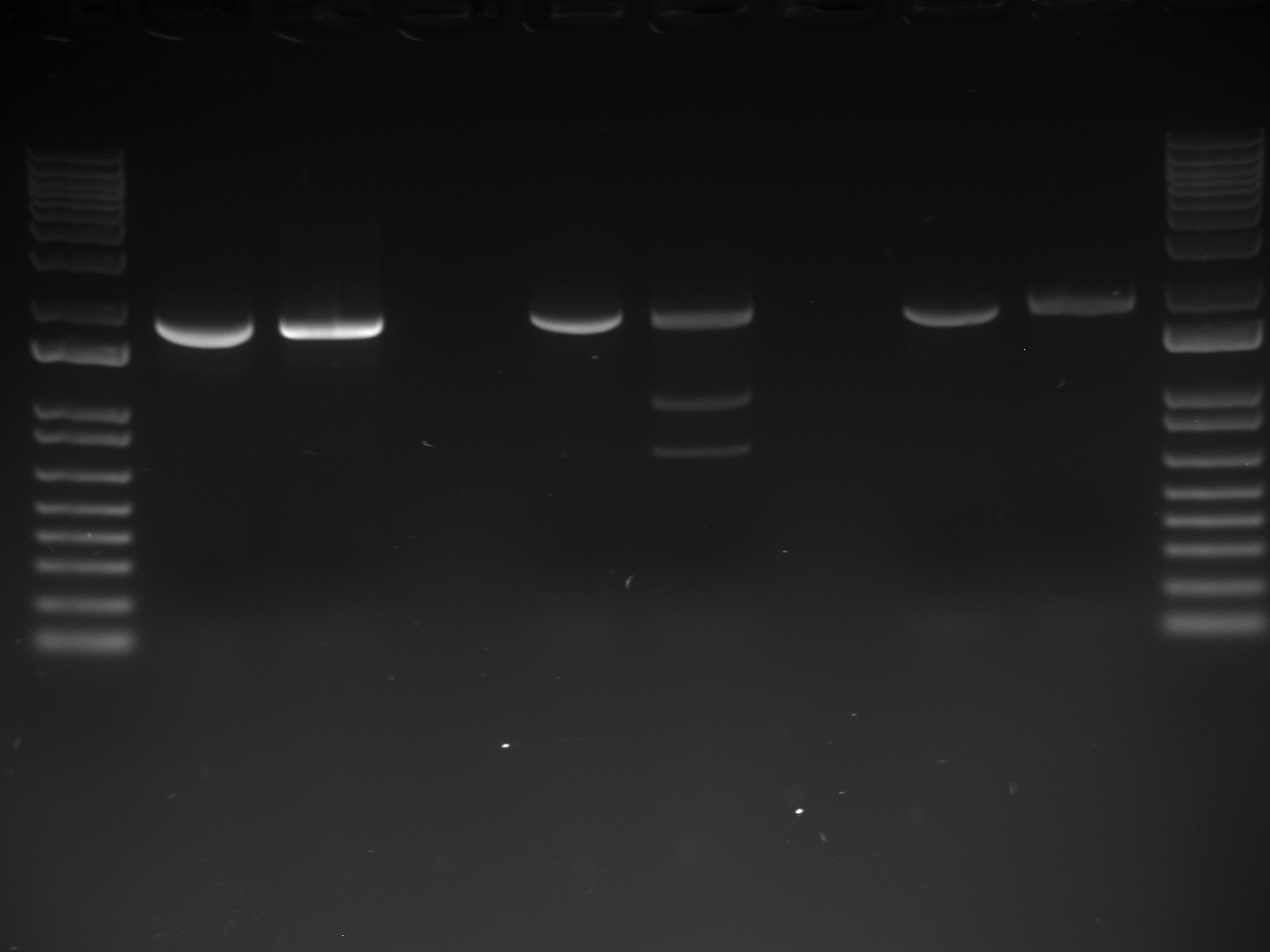

Supplement: Supplementary file 4 — Source Data [file 41467_2023_41476_MOESM4_ESM.zip › SOURCE DATA/SUPPLEMENTARY DATA/Supplementary Figure 1/Supplementary figure 1c_FR6M_mixed pool.tif]

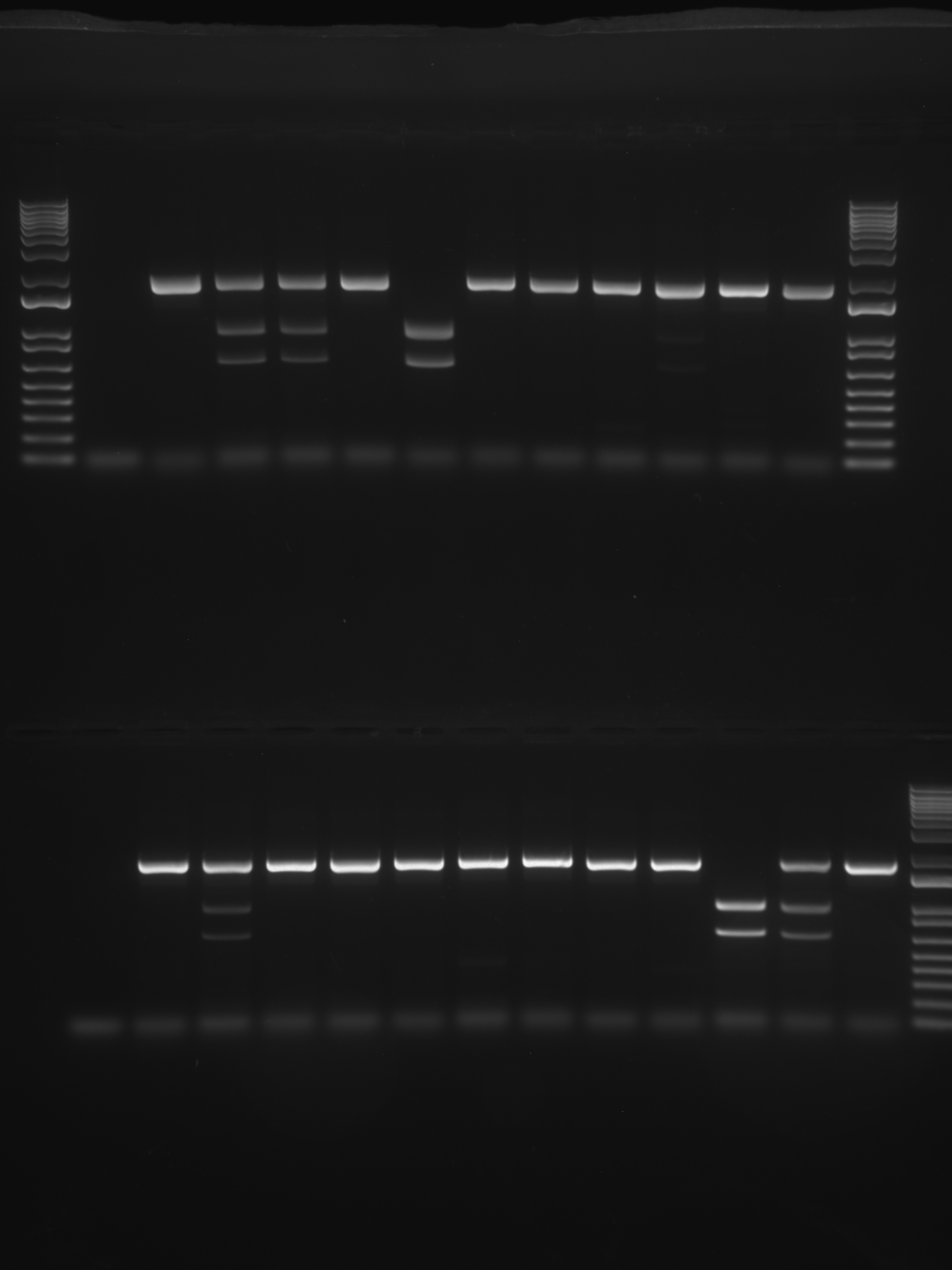

Supplement: Supplementary file 4 — Source Data [file 41467_2023_41476_MOESM4_ESM.zip › SOURCE DATA/SUPPLEMENTARY DATA/Supplementary Figure 1/Supplementary figure 1d_FR6F-top-panel_&_figure 1f_FR3F-bottom-panel-last_six_lanes.jpg]

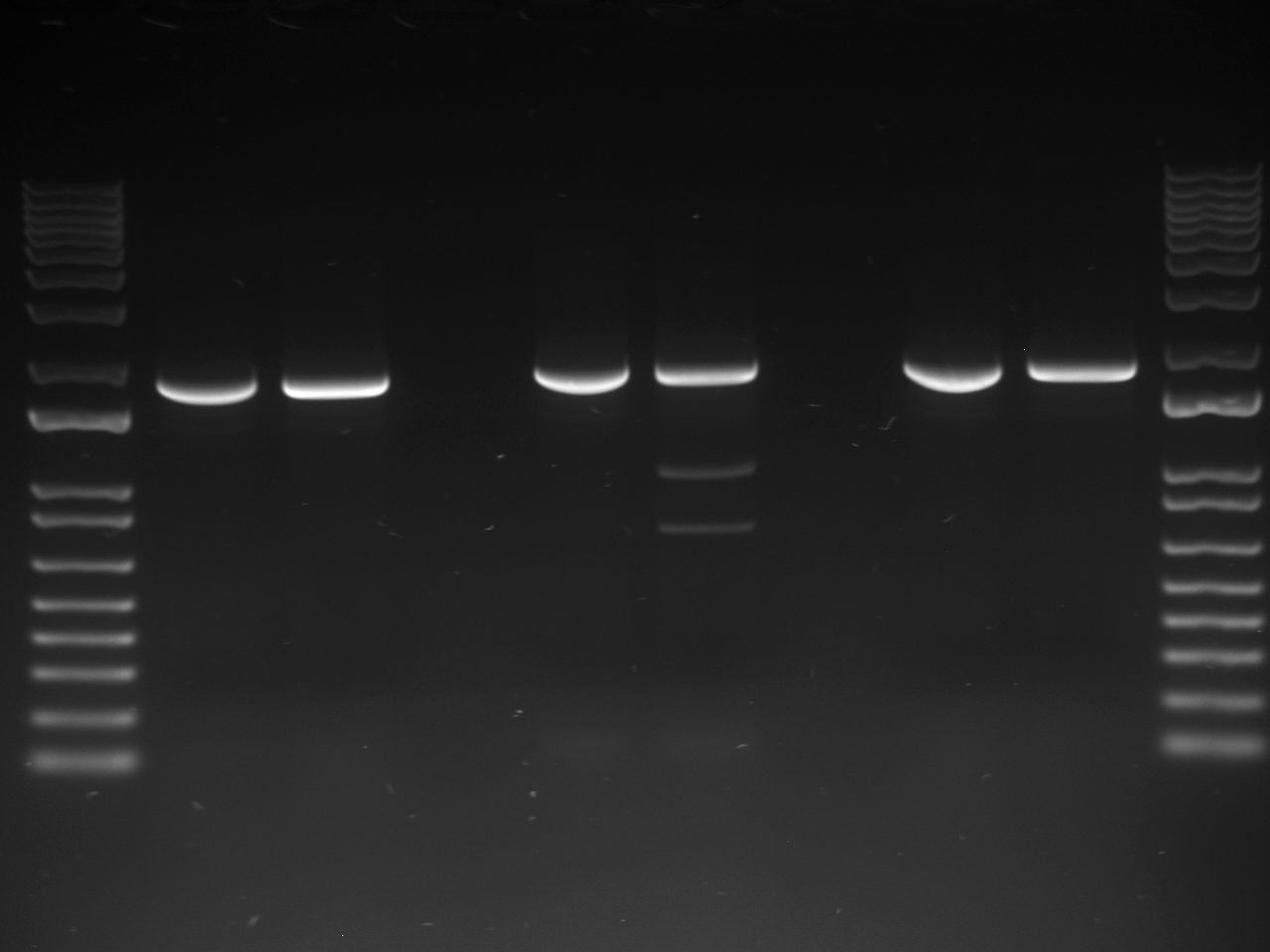

Supplement: Supplementary file 4 — Source Data [file 41467_2023_41476_MOESM4_ESM.zip › SOURCE DATA/SUPPLEMENTARY DATA/Supplementary Figure 1/Supplementary Figure 1e_FR3F_mixed pool.tif]

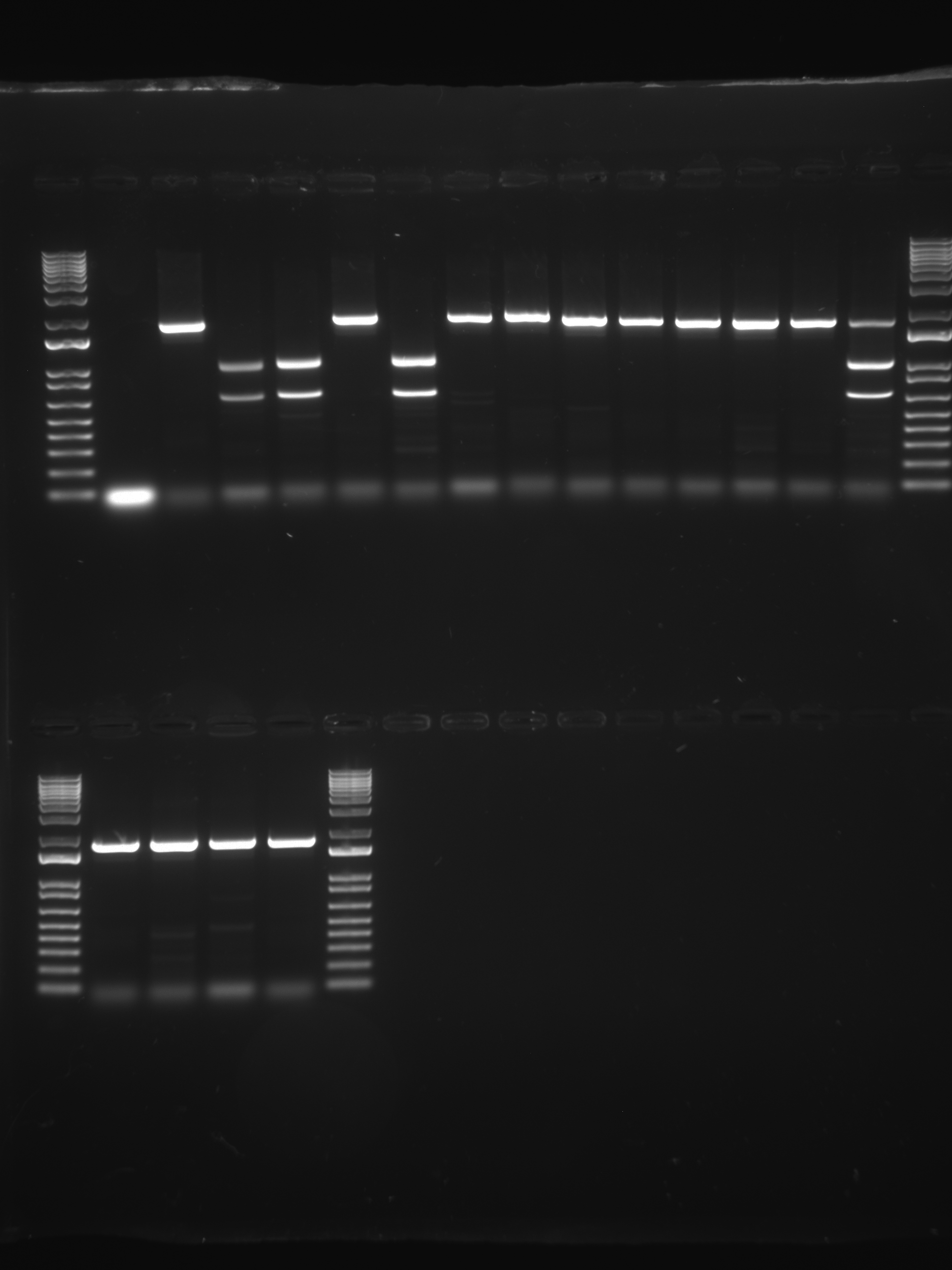

Supplement: Supplementary file 4 — Source Data [file 41467_2023_41476_MOESM4_ESM.zip › SOURCE DATA/SUPPLEMENTARY DATA/Supplementary Figure 1/Supplementyary figure 1f_part 1_FR3F_top-panel.tif]

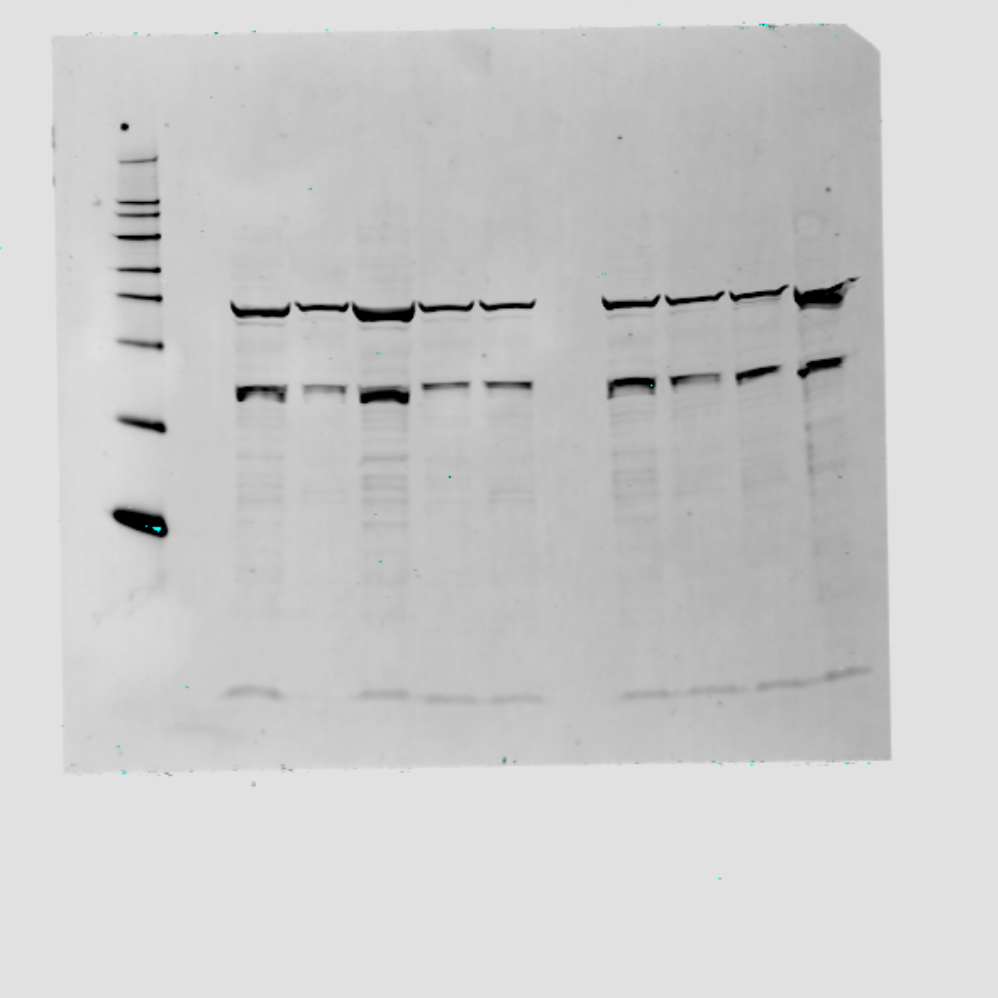

Supplement: Supplementary file 4 — Source Data [file 41467_2023_41476_MOESM4_ESM.zip › SOURCE DATA/SUPPLEMENTARY DATA/Supplementary Figure 3/Supplementary Figure 3_FR3F clones_left-panel_0011614_01.png]

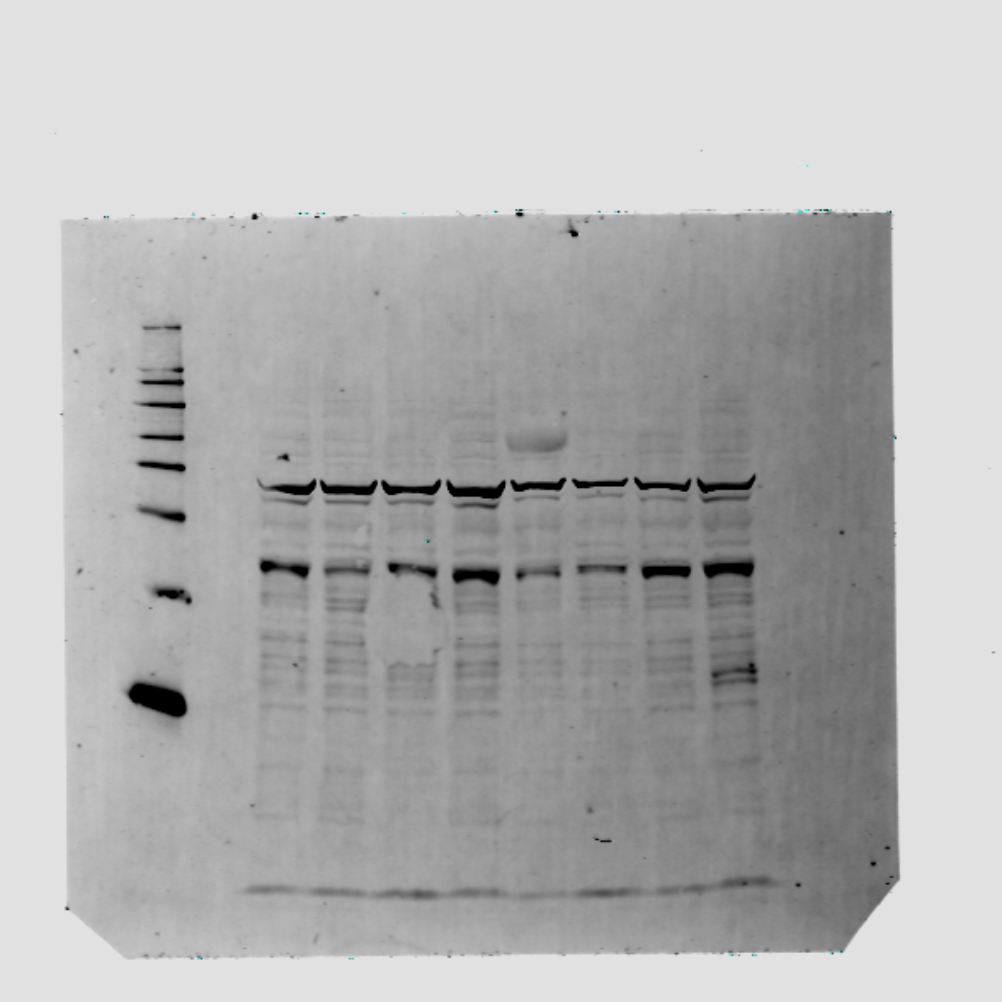

Supplement: Supplementary file 4 — Source Data [file 41467_2023_41476_MOESM4_ESM.zip › SOURCE DATA/SUPPLEMENTARY DATA/Supplementary Figure 3/Supplementary Figure 3_FR3F clones_left-panel_0011616_02.tif]
